# Differential Protein Expression in Berry Skin from Red Grapes with Varying Hybrid Character

**DOI:** 10.3390/ijms23031051

**Published:** 2022-01-19

**Authors:** Valentina Spada, Luigia Di Stasio, Pasquale Ferranti, Francesco Addeo, Gianfranco Mamone, Gianluca Picariello

**Affiliations:** 1 Istituto di Scienze dell’Alimentazione—Consiglio Nazionale delle Ricerche (CNR), Via Roma 64, I-83100 Avellino, Italy; valespada15@libero.it (V.S.); luigia.distasio@isa.cnr.it (L.D.S.); ferranti@unina.it (P.F.); 2 Dipartimento di Agraria, Università di Napoli “Federico II”, Parco Gussone, I-80055 Portici, Italy; addeo@unina.it

**Keywords:** red grape, berry skin, hybrid grapes, proteomics, glucosyltransferases

## Abstract

Protein expression from the berry skin of four red grape biotypes with varying hybrid character was compared at a proteome-wide level to identify the metabolic pathways underlying divergent patterns of secondary metabolites. A bottom-up shotgun proteomics approach with label-free quantification and MaxQuant-assisted computational analysis was applied. Red grapes were from (i) purebred *Vitis vinifera* (Aglianico *cv*.); (ii) *V. vinifera* (local Sciascinoso *cv*.) grafted onto an American rootstock; (iii) interspecific hybrid (*V. vinifera* × *V. labrusca*, Isabel), and (iv) uncharacterized grape genotype with hybrid lineage, producing relatively abundant anthocyanidin 3,5-*O*-diglucosides. Proteomics supported the differences between hybrids and purebred *V. vinifera* grapes, consistently with distinct phenotypic metabolite assets. Methanol *O*-anthraniloyltransferase, which catalyses the synthesis of methyl anthranilate, primarily responsible for the “foxy” odour, was exclusive of the Isabel hybrid grape. Most of the proteins with different expression profiles converged into coordinated biosynthetic networks of primary metabolism, while many possible enzymes of secondary metabolism pathways, including 5-glucosyltransferases expected for hybrid grapes, remained unassigned due to incomplete protein annotation for the *Vitis* genus. Minor differences of protein expression distinguished *V. vinifera* scion grafted onto American rootstocks from purebred *V. vinifera* skin grapes, supporting a slight influence of the rootstock on the grape metabolism.

## 1. Introduction

The grape phylloxera aphid (*Daktulosphaira vitifoliae* Fitch) was introduced unintentionally into Europe in the early 1860s through infected plant material from North America [1]. The rapid spreading of this pest decimated European vines, destroying approximately 2.5 million hectares of vineyards [2].

The wild American grapevine species that have co-evolved with phylloxera tolerate this aphid pest with varying levels of resistance. However, grapes of wild American species are unsuitable to produce any wine-like drink, and their resistant interspecific crosses with European *Vitis vinifera* (the so-called French hybrids) carry many of the defects of their American ancestors, such as low sugar content, a “foxy” odour and off-flavours, as well as poor tannin content and availability [3].

Grafting *V. vinifera* scions onto resistant rootstocks of American Vitis species (e.g., *Vitis rupestris*, *Vitis labrusca*, and *Vitis riparia* or their interspecific hybrids) allowed saving the European wine heritage, combining typical European oenological traits with the resistance against phylloxera and providing the most effective solution for overcoming the vineyard pandemic [4]. The technique of grapevine grafting is still a routine practice in Europe as well as worldwide, and it is also applied to enhancing the tolerance to environmental and abiotic stresses, such as soil limestone, salinity, stagnation, drought, and frost.

The metabolic profiles of wild American and *V. vinifera* grapes significantly diverge in many respects. For instance, unlike European grapes, some wild American species accumulate hydrolysable tannins, lignans, and the typical volatile compounds such as methyl anthranilate responsible for the “foxy” smell [5]. Due to undesired metabolites and presumably to safety issues related to a harmful content of methanol in wine, the EU regulations have prohibited the growth of American grape varieties and the direct production of wine from hybrids [6], although environmental, cost and diversification concerns are currently leading grapevine breeders to reconsider hybrid grapes for making wine.

The different phenotypic expression of metabolites reflects genotypic divergences and, consequently, distinct enzymatically regulated biosynthetic pathways. In terms of metabolites, one of the most important differences between American hybrids and *V. vinifera* red grapes concerns the qualitative and quantitative expression of anthocyanins. Red grapes from traditional *V. vinifera* “elite” varieties accumulate only 3-*O*-monoglycosylated anthocyanins, in part esterified with *p*-coumaroyl or caffeoyl moieties at the 6-*O*-glucoside position. American *Vitis* species (including *V. labrusca, V. riparia* and *V. amurensis*) display more complex anthocyanins patterns than *V. vinifera* because they express 3,5-*O*-diglycosylated anthocyanins along with their 6-*O*-esterified derivatives, in addition to relatively abundant acetylated 3-*O*-glycosyl derivatives [5,7,8]. The interspecific hybrids of red grapes (e.g., *V. vinifera* × *V. labrusca*, that is Isabel grape) express 3,5-*O*-diglycosylated anthocyanins at variable amounts [9].

The gene encoding for a site-specific 5-*O*-glycosyltransferase (5-GT) expresses different alleles between *V. vinifera* and wild American grapes, resulting in distinct phenotypic anthocyanin profiles. In most *V. vinifera* varieties, *5-GT* alleles contain loss-of-function mutations, which prevent the enzyme from being expressed [10,11]. Xing et al. provided mass spectrometric and enzymatic evidence for the occurrence of anthocyanidin 3,5-*O*-diglucosides at trace amounts in *V. vinifera* Cabernet Sauvignon berries [12]. However, these authors did not indicate whether the presence of the anthocyanidin 3,5-*O*-diglucosides should be ascribed to a low-level expression of 5-GT active variants, to some non-specific activity of 3-*O*-glycosyltransferase (3-GT) or to the possible metabolic influence of the rootstocks.

Rootstocks are known to significantly affect the physiology of grapevine, for example modifying the mineral element profile of the scion [13] and the concentration of key constituents of grape seeds [14]. In particular, the rootstock affects the metabolic signalling of abscisic acid and stilbenoids, as confirmed with transcriptomic and metabolic studies on grape leaves [15] and berry [16]. However, the role of rootstocks in determining the biosynthesis of signature metabolites of wild American grapes has been not investigated thoroughly and the effects on vine’s performance are still controversial [17]. More in general, several efforts have been made to investigate the dynamics of coordinated gene expression in grapevines at both genomic and transcriptomic levels [15,16,18,19,20,21]. In contrast, to date, relatively few proteomic studies have been conducted on grape, mostly based on the “classical” two-dimensional gel electrophoresis-based proteomics [22,23,24,25]. The recently developed proteomic technology represents a powerful tool to elucidate the regulatory networks underlying expression of metabolites in grape. Combined with other -omic technologies, proteomics was able to differentiate five *V. vinifera* grape cultivars. However, while the accumulation of metabolites corresponded to the activation of specific enzymatic pathways, the protein expression did not correlate with the transcript abundance [26]. Wild American and European grape proteomes have not been compared with dedicated studies, so far.

In a previous paper, using mass spectrometry we demonstrated that the metabolite expression profiles of berry skin from *V. vinifera* vines grafted onto American rootstocks included very low levels of anthocyanidin 3,5-*O*-diglucosides, which were missing in purebred *V. vinifera* cultivars [8]. Herein, the protein extracts from berry skin have been characterized and compared at a proteome-wide level in four different biotypes of red grapes, including: (i) a purebred *V. vinifera* (Aglianico *cv*.); (ii) *V. vinifera* grafted onto an American rootstock; (iii) an interspecific hybrid (*V. vinifera* × *V. labrusca*, Isabel), and (iiii) an uncharacterized vine with some hybrid lineage producing relatively high amounts of anthocyanidin 3,5-*O*-diglucosides. The accurate assessment of difference in protein quantitative expression among grapes with different genomic assets and growth under inhomogeneous pedoclimatic environments was outside the scope of this work. The aim of this work was to target the differences in protein expression among red grape biotypes with varying hybrid character, also addressing the possible effects of the grafting on the phenotypic expression of some key genes within the diverse groups of secondary metabolic pathways in grape berries. The main purpose of this investigation was to identify possible enzymatic pathways distinctive of the hybrid character of grapes.

## 2. Results and Discussion

**Figure 1:**
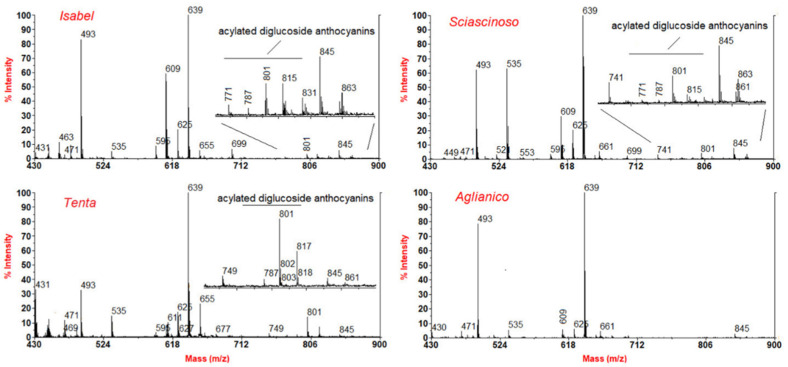
Comparative MALDI-TOF MS anthocyanin analysis of methanol extracts from grape berry skins. Acylated diglucoside anthocyanins have been monitored as metabolite markers of the hybrid traits. The relevant signals were missing in purebred *V. vinifera* (Aglianico *cv*.), while in the remaining samples their intensity varies in the order Isabel > Tenta > Sciascinoso.

In the present study, the hybrid trait of red grapes was assessed by matrix-assisted laser desorption ionization-time of flight mass spectrometry (MALDI-TOF MS) analysis of the berry skin anthocyanins (Figure 1). Acylated (i.e., *p*-coumaroyl and caffeoyl derivatives) anthocyanin 3,5-*O*-diglucosides were monitored in methanol extracts of berry skin as species-specific markers of the hybrid grapes [27]. MALDI-TOF MS is more sensitive than HPLC for detecting these compounds, which, in some cultivars, occur in scant amounts [8]. The intensity of MS signals relevant to acylated 3,5-*O*-diglucoside anthocyanins, compared with malvidin 3-*O*-(6-*O*-*p*-coumaroyl)glucoside (M^+^ = 639), yielded the most intense signal in all the cases, decreasing according to following order: Isabel > Tenta >> Sciascinoso (only in trace amounts). In contrast, 3,5-*O*-diglucoside anthocyanins were absent in *V. vinifera* Aglianico *cv.* These findings confirmed that Isabel has a hybrid nature, Aglianico is a purebred *V. vinifera* genotype and Sciascinoso grape expresses minor hybrid traits, while Tenta is presumably a (non-*V. vinifera* × *V. vinifera*) × *V. vinifera* hybrid, in agreement with previous HPLC and MS analyses [8].

Anthocyanins are biosynthesized in the skin (exocarp) cells. Grape berry skin, as a metabolic active tissue, biosynthesizes a plethora of additional compounds with several physiological functions, thus concurring critically to determine organoleptic characteristics of wine. Exocarp tissue shows higher abundance of transcripts for genes involved in flavonoid biosynthesis, pathogen resistance and cell wall modification compared with other berry and plant tissues [28]. Since we were mainly interested in the enzymatic pathways producing volatile and non-volatile metabolites and those responsible for anthocyanin accumulation that distinguish hybrid from *V. vinifera* grapes, our proteomic analysis was focused on the grape skin.

### 2.1. Protein Identification and Clustering

Overall, 1763 gene products were confidently (FDR 1%) identified in the skin of the four grape samples using a shotgun proteomic approach and identification with the Andromeda search engine. After log transform and filtering by homology, 1434 entries were identified with at least two peptides, among which 1153 were contained in at least two out three replicates.

Based on a post-hoc one-way ANOVA test, 892 proteins had significantly modified levels of expression in at least one sample and were considered for further analysis. The proteins filtered out, corresponding to proteins with comparable expression among the samples, were mainly involved in pathways of primary metabolism, such as glycolysis, gene transcription and expression, protein processing and amino acid metabolism, as assessed by STRING pathway analysis and KEGG functional classification (Supplementary material Figure S1).

**Figure 2:**
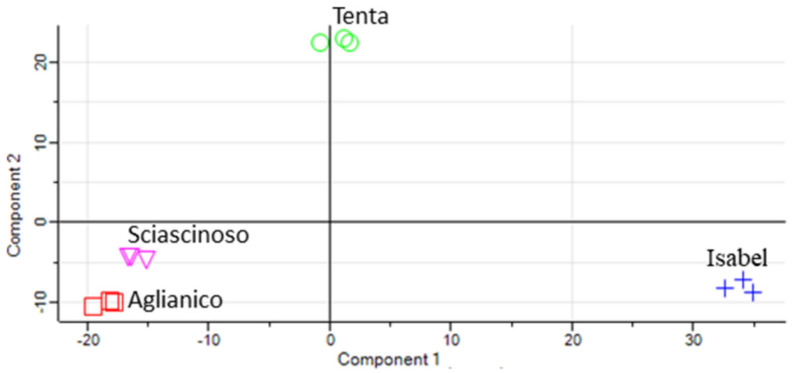
PCA score scatter plot based on proteomics of berry skin from Isabel (blue cross), Tenta (green circle), Sciascinoso (pink triangle) and Aglianico (red ring) grape biotypes. Three replicate samples for each grape variety.

A principal component analysis (PCA) was performed to establish the interdependence among the proteomes of the four grape varieties. The resulting PCA score scatter plot is illustrated in Figure 2. The PC1 and PC2 explained 51.5% and 20.6% of the total variance relevant to the entire protein set, respectively. Aglianico and Isabel samples clustered at the highest mutual distance in the scatter plot, whereas Sciascinoso was relatively close to the Aglianico sample. The proteome of Tenta grape had intermediate characteristics between those of Aglianico and Isabel, in line with the metabolic assessment [8]. The close proximity of loadings belonging to the same grape varieties, indicated a high level of reproducibility as well as homogeneity of replicate samples, also evidenced by Pearson’s correlation coefficients between biological replicates >0.95 (not shown).

**Figure 3:**
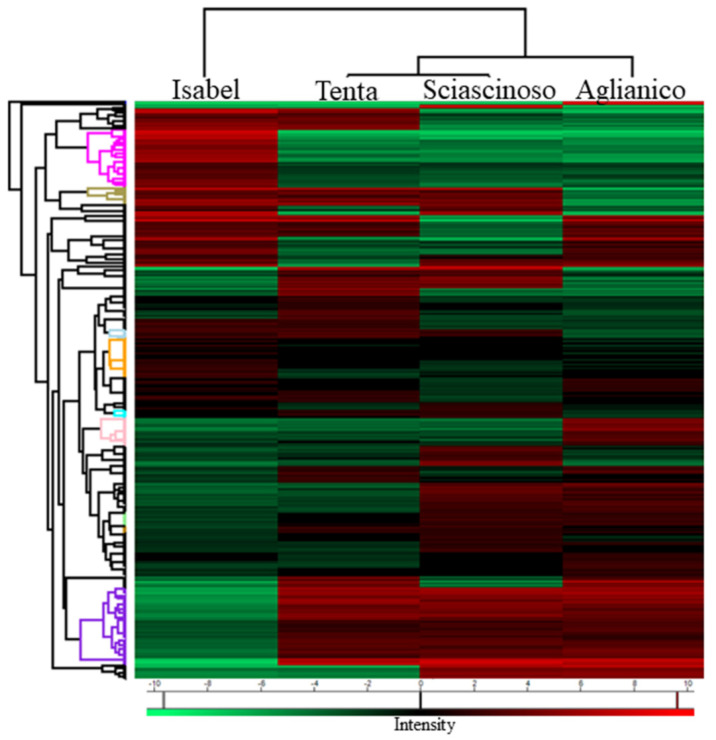
Heat map for the hierarchical clustering of gene products found to have differential expression among the four grape skin samples. Expression levels have been log-transformed and are highlighted with in red (high) or green (low) colours. Overall, 11 clusters were generated in the horizontal dendrogram, indicating different profiles of protein expression. Each row represents individual gene products.

**Figure 4:**
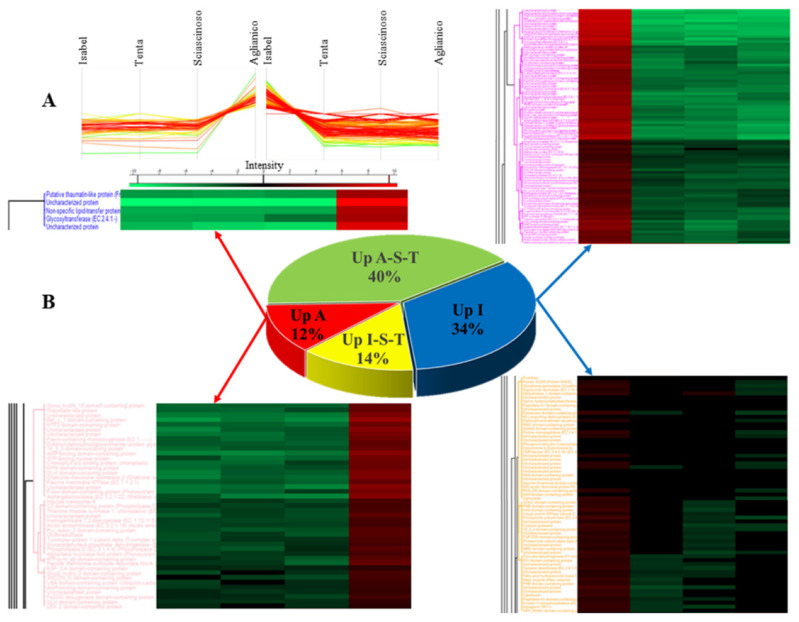
(**A**) Profile plots of four representative selected clusters (two clusters up regulated in Isabel and two cluster in Aglianico) showing distinct behaviour with respect to the four red grapes and (**B**) details of the heat maps relevant to gene products associated with the categories “Up-Aglianico” and “Up-Isabel”.

The heat map generated by hierarchical clustering analysis revealed that the four grape biotypes had significantly distinct patterns of protein expression (Figure 3). Consistently with the results of the PCA, the proteomes of Aglianico and Sciascinoso grapes clearly exhibited a common ancestry, and both had substantial phenotypic divergence from Isabel. The proteome of Tenta has intermediate traits between that of *V. vinifera* and the interspecific hybrid Isabel. From the general list of proteins identified by LC-MS/MS, 11 “main clusters” were extrapolated especially based on different profiles of protein expression between Aglianico and Isabel grapes (Figure 4).

Protein entries grouped by clusters are listed in Supplementary material Table S1. This Table also lists 19 “secondary clusters” grouping gene products differentially expressed in any of the samples or in relevant pairs of grape biotypes with a 1.5-fold change threshold level. Since pooled samples were analysed, the fold-change threshold was not set not very high to prevent loss of information [29]. However, we emphasized the “macroscopic” differential expression between biotypes. The features of the clusters are summarized in Supplementary material Table S1.

### 2.2. Interspecific Hybrid Grape (Isabel) Signature Gene Products

The cluster indicated with “−872” grouped proteins with relatively high levels of expression in the Isabel grape and negative scores in the remaining samples. In other terms, this cluster comprises gene products over—or exclusively expressed in—Isabel compared with the remaining grape biotypes. Notably, these proteins comprised methanol *O*-anthraniloyltransferase (AMAT) from *V. labrusca*, an acyltransferase that catalyzes the conversion of anthraniloyl-CoA and methanol into methyl anthranilate, that is the volatile compounds mainly responsible of the “foxy” odor characteristic of Concord (*V. labrusca*) and derived hybrid grapes. Since this enzyme should be expressed in berry outer mesocarp, but not in berry skin [30], its presence in the current dataset might suggest an incomplete mechanical separation of the two berry tissues. The absence of the unpleasant foxy odor tract in *V. vinifera* grapes, both purebred and grafted biotypes, is consistent with the lack of the biosynthetic machinery of AMAT in Aglianico. Since AMAT has a broad substrate specificity, it is expected to be a key enzyme in the biosynthetic pathway of ethyl-3-hydroxy butanoate as well, which represents an additional volatile signature of the *V. labrusca* grape [30].

*V. labrusca* glycosyltransferase (Uniprot accession Q0PI14_VITLA) belonging to the cluster −872 was one of the razor gene products exclusively expressed in Isabel grape. The expression of this glycosyltransferase has been confirmed at gene and transcript level but never demonstrated at the protein one.

Glucosyltransferases (GT) constitute a large, multi-gene family of cytosolic enzymes that intervene in the final steps of flavonoid biosynthesis. GT catalyse the transfer of glucose from uridine 5′-diphosphoglucose (UDPG) to several plant metabolites with a regioselective substrate specificity. Glycosylation has key importance in plants, as it contributes to protecting the aglycones against enzymatic oxidative degradation by polyphenol oxidases within the plant cells, also increasing their stability and reducing their turnover rate. The GT share a conserved signature motif of sequence (PSPG box) that can be used to predict their function from genes. Several studies have been carried out to classify GT genes in grape. At present, 3-GT from Concord (*V. labrusca*) grape has been cloned, and structurally and functionally characterized [31].

In contrast, isolation and characterization of *5-GT* genes or their coded enzymes have been reported only from a limited number of grape germplasms. For instance, He et al. characterized an anthocyanin 5-GT from *Vitis amurensis* (Uniprot, W8QG94_9ROSI) [32].

Q0PI14_VITLA (*V. labrusca*) corresponds to the resveratrol/hydroxycinnamic acid-*O*-GT expressed in berry mesocarp during the late stages of Concord grape ripening. Q0PI14_VITLA shares high degree of sequence homology with a *V. vinifera* quercetin-3-*O*-glucosyltransferase (Uniprot, F6H660_VITVI), which catalyses the glycosylation of stilbenoids and flavonoids rather than skin anthocyanins. On the other hand, these enzymes display broad substrate regioselectivity in vitro and might transfer glycoside groups to a set of metabolites, including non-anthocyanin flavonoids [33]. In general, the identification of a panel of possible multiple homologous gene products instead of the specific proteoforms occurring in a given proteome is a major limitation of the bottom-up shotgun proteomic approach. Thus, the specific enzyme(s) responsible of the 5-*O*-GT activity in Isabel grape should be addressed with targeted investigations. Similar challenges to identify the specific enzyme responsible of the 5-GT activity have been pointed out at the transcriptomic level [34]. Interestingly, at least ten gene products were functionally classified as glycosyltransferases by the software Blast2GO. Among these, only one protein, i.e., D7T7R5_VITVI, exclusively expressed in the Sciascinoso, was included in the metabolic pathway of grape anthocyanins, and classified as a 3-GT.

The presence of other members of the GT family could remain undetected depending on the relatively low levels of protein expressed in grape and large protein concentration dynamic range hindering proteomic analysis. In alternative, it is possible that due to large number of glycosyltransferases with broad substrate specificity and loose regioselectivity, the glycosylation reaction is accomplished, for example, by 3-GT instead of 5-GT [12]. In any case, these data demonstrate that grape protein annotation and classification are still largely incomplete. The fine regulation of the phenylpropanoid pathway still requires to be fully elucidated for *V. vinifera* and even more so for other *Vitis* species [35].

Comparing the dynamic evolution of berry skin proteome from red and white grapes, Niu et al. found that several key enzymes involved in the synthesis of secondary metabolites, including GT, accumulate before or at veraison, while decading at maturity [24]. Conversely, the last ripening stage entails an increased expression of enzymes involved in the primary metabolism, such as those participating to the glycolytic pathway [22] and those involved in mechanisms of defence against pathogens. Therefore, in our protein extracts from grapes harvested at full maturity GT might be relatively low abundance.

The individual protein entries can be visualized within their relevant clusters, as exemplified for the cluster “−872” extrapolated from the global heat map, in Supplementary material Figure S2, where gene products can be visualized along with their UniprotKB name. Both AMAT and the putative hybrid-specific GT are evidenced in Supplementary material Figure S2.

**Figure 5:**
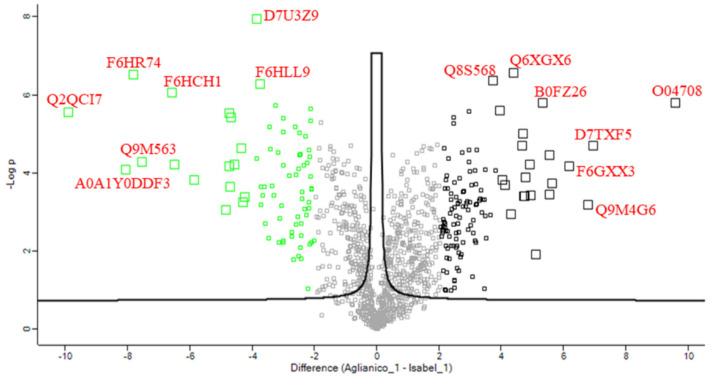
Volcano plot indicating up-regulated (**right**) and down-regulated (**left**) gene products in Aglianico vs. Isabel grapes. Differentially expressed proteins with fold-change >4 or <−4 have been labelled with their UniprotKB accession number.

The selective expression of several other gene products in Isabel is likely the consequence of different adaptative requirements of the grape, rather than the phenotypic manifestation of characteristic active metabolic pathways. This finding emerges clearly from the volcano plot of Figure 5, which evidences the gene products with high relative level of expression differentiating Isabel (right side) from Aglianico (left side). The characteristic protein most expressed were thaumatin-like (Uniprot entries O04708 and Q9M4G6), pathogenesis-related protein 4 (Uniprot D7TXF5) and lipid tranfer proteins (Uniprot entries F6GXX3 and Q2QCI7), which are defence-related enzymes. Interestingly, thaumatin-like proteins were highly expressed in Isabel, probably in relation to its higher rate of resistance against several pathogens [36]. Other gene products with high levels of expression were enzymes involved in protection from oxidative damage, such as catalase (Uniprot entry Q8S568), glutathione transferase (Uniprot entry F6HR74) and dioxygenase with oxidoreductase activity (Uniprot entry F6HCH1). The functional assignment of selected gene products by homology demonstrate that also other uncharacterized proteins were pathogenesis-related proteins, including thaumatin and cysteine-protease inhibitors (cystatins), which generally are expressed in variable amount in response to environmental stress factors. The impossibility of deriving comprehensive biosynthetic networks even from detailed proteomic analysis could depend on the incomplete functional assignment of many gene products, which yet remain “uncharacterized” (Supplementary material Figure S2).

A series of proteins expressed at intermediate levels in Isabel grape and lacking or down-regulated in the remaining samples is grouped in the cluster “−844”. Most of these proteins matches the *V. vinifera* databases, but they are functionally uncharacterized. Malic enzyme, belonging to this class, is activated during the acid-accumulating stage of berry development and might justify the high concentration of malic acid detected in hybrid grapes, although it is susceptible to enzymatic catabolism during ripening [37]. Consistently, malate dehydrogenase that is involved in the catabolism of malic acid was expressed/up-regulated in all the grape biotypes but Isabel.

In general, Isabel expresses enzymes involved in the synthesis and balance of glutathione (e.g., glutathione S-transferase, glutathione peroxidase), which physiologically concur to the redox homeostasis. Furthermore, glutathione-binding proteins and in particular glutathione S-transferase act as anthocyanin transporters, thereby affecting berry skin pigmentation [38]. Glutathione plays a critical role in preventing the development of atypical off-flavours during wine fermentation and aging as well as for the overall wine stability. Glutathione content varies according to the grape variety, and the upregulation of the relevant biosynthetic enzymes in Isabel could justify higher concentration of this potent antioxidant tripeptide in berries with increasingly wild (*V. labrusca*) genetic traits. In agreement with these hypotheses, it has been recently demonstrated that sparkling wine produced with purebred *V. labrusca* grape (Niagara variety) contains significantly higher amount of total glutathione (reduced plus oxidized form) than wine of other non-traditional *V. labrusca* × *V. vinifera* grape hybrids [39]. Interestingly, in addition to other glutathione transferases detected in other biotypes, one protein with catalogued glutathione transferase activity (UniprotKB, A5BM22_VITVI) occurred exclusively in the Sciascinoso grape variety.

**Figure 6:**
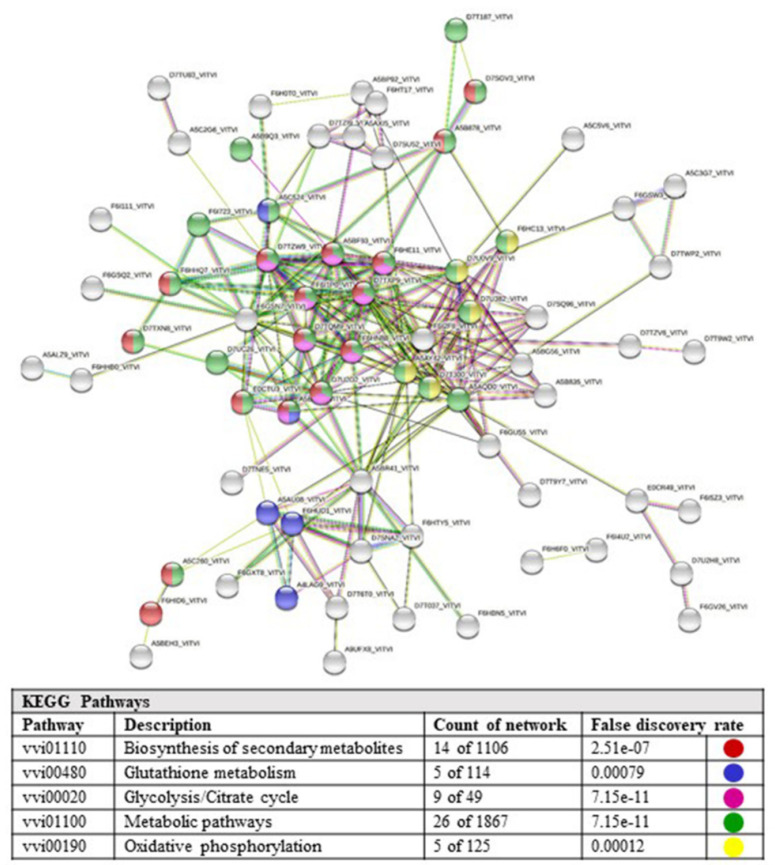
Protein–protein interaction (PPI) map generated by STRING showing the interactions of 117 proteins out of total of 133 genes up-regulated/exclusively expressed in Isabel grape, with 228 edges. The line thickness indicates the strength of data support. The coloured nodes represent five different KEGG functional groups. Proteins are labelled with the UniprotKB name.

The list of genes (131 entries) corresponding to over-/exclusively expressed proteins in Isabel grape (clusters number “−872” and “−844”) were entered into the STRING database, which mapped 131 genes into protein–protein interaction (PPI) networks containing 104 nodes and 172 edges (Figure 6). Clustering of gene products and functional classification were performed with GO and KEGG to define possible enzymatic pathways involved in the synthesis of secondary metabolites.

KEGG functional analysis showed that three nodes (3%) belong to glycolysis/glucogenesis, 16 nodes (15%) to biosynthesis of secondary metabolites and 23 (22%) to general metabolic pathway. Central hubs represent the glycolysis, oxidative phosphorylation and redox enzyme networks. It was not possible to infer exclusive biosynthetic pathways of secondary metabolism through the functional assessment of expressed or up-regulated gene products in Isabel grape, due to the scarcity of reference information relevant to individual gene products and their integration in enzymatic networks.

### 2.3. Functional Divergences among Grape Biotypes

The cluster indicated with “−873” consists of 27 gene products upregulated in Isabel, Tenta and Sciascinoso, which were not expressed or were down regulated in Aglianico grape skin. Interestingly, many among the functionally characterized proteins included in this cluster are implicated in cellular redox-dependent processes, such as the enzymes involved in the ascorbate metabolism. Ascorbate, as a potent plant antioxidant, fulfils numerous functions in different cellular components. However, unlike most other fruits, grape uses ascorbate as a precursor of oxalic and, especially, tartaric acids, as recently reviewed [37].

Genes coding for the enzymes involved in biosynthesis, recycling and catabolism of acids in grape berries are developmentally regulated and their activity varies greatly with the grape ripening stages [40]. The resulting metabolic processes lead to the accumulation of high levels of tartaric acid as a typical end-product. The enzymatic pathways controlling sugar and acid production in grapes are interconnected and extremely complex, and also influenced by a variety of environmental factors [41]. However, in general, the enzymatic apparatus is up-regulated in grape biotypes with “hybrid” character with respect to *V. vinifera* grapes, consistently with the much higher acidity developed in hybrid grapes and with a reduced biosynthesis of tartaric acid that can generate increased levels of vitamin C in *V. vinifera* berries [42]. The proteomic analysis of flesh can expectedly give more in-depth indications about the enzymes that regulate the synthesis and accumulation of acids in grapes.

Free galactose does not significantly change between hybrid and *V. vinifera* grapes. Thus, the presence of UDP-glucose 4-epimerase (Uniprot, A5AK58_VITVI) at expression levels decreasing with the hybrid character (Isabel > Tenta > Sciascinoso, absent in Aglianico) might explain the higher amounts of flavonol (quercetin, kaempferol, myricetin) galactosides, occasionally found in hybrid grapes compared to the *V. vinifera* counterpart [43]. Interestingly, UDP-glucose 4-epimerase is homologue to the so-called CASP (Casparian Strip membrane Domain Proteins)-like proteins, which are transmembrane plant enzymes involved in the polymerization of lignin.

The most plentiful cluster, namely cluster “−879” (120 entries), grouped proteins expressed in all the grape biotypes other than Isabel. In addition to enzymes of the primary metabolism, many of these proteins were ribosomal enzymes, supporting the differences in the mechanisms of protein synthesis afforded by the wild character of *V. labrusca* compared to the *V. vinifera* varieties.

The enzyme anthocyanin-*O*-methyltransferase (AOMT), belonging to this cluster, controls the level of hydroxylation and methylation of berries in grape. By this way, AOMT affects the chromatic properties of pigments, contributing to protection from photo-oxidative stress as well as to stability and reactivity of anthocyanins [44].

Proteins expressed/up-regulated exclusively in Aglianico grape and down-regulated or absent in all the remaining grape samples were grouped in the clusters “−863” (n. 43 entries) and “−797” (n. 5 entries). Among these, a few enzymes involved in the biosynthesis of secondary metabolites occurred, also including a GT. The corresponding STRING analysis did not show significant networks of secondary metabolism (not shown). Sciascinoso berry skin (cluster “−793”, n. 6 entries) contained an up-regulated anthocyanin acyltransferase (Uniprot, D7TU67_VITVI), which catalyses acylation of anthocyanins, thus enhancing their structural diversity and stability, and a glutathione transferase isoform (Uniprot, A5BM22_VITVI), likely involved in the transport of anthocyanins from endoplasmic reticulum to the vacuole [34]. Enzymes involved in similar metabolic processes are expected for the other grape samples and are probably catalogued in Table S1 as acyl-carriers, acetyltransferases or carboxypeptidase-like acyltransferases [34]. Sciascinoso grape (cluster “−862”, n. 31 entries) contained chalcone synthase, chalcone-flavonone isomerase, phenylalanine ammonia-lyase and UDP-glucose flavonoid 3-*O*-GT (fragment), which are all upstream enzymes of the flavonoid and anthocyanin biosynthetic pathways. The identification of these enzymes suggests that Sciascinoso grapes might express a variety-specific pattern of secondary metabolites. Isoforms of these enzymes were shared with Aglianico grapes. Notably, in addition to UDP-glucose flavonoid 3-*O*-GT, chalcone synthase 3 (CHS3) and downstream enzymes, such as glutathione S-transferase, have been found associated with the pink colour of grape berry skin [45]. However, a characteristic expression of these enzymes is likely related to the specific developmental stage of Sciascinoso grape since the expression of phenylalanine ammonia-lyase, chalcone flavanone isomerase and a flavanone oxidoreductase is modulated simultaneously during anthocyanin accumulation [46]. Aglianico and Sciascinoso grapes shared a relatively high number of comparably expressed gene products (clusters “−824”, “−823” and “−861” with n. 46, 17 and 22 entries, respectively), likely justifying their phenotypic similarity. The analysis of pathways with STRING did not reveal coordinated enzyme subnetworks for these enzymes. Subtle differences between Aglianico and Sciascinoso grape might stem from the intercultivar variability, at least in part due to slightly different genomic assets of the two different grape cultivars, besides the influence of the rootstock on the metabolism in Sciascinoso [26].

Since the grouping features of these secondary clusters were less neat than for main clusters, pairwise profiles of proteins with comparable degrees of expression were extrapolated with the “profiles” tool of Perseus. The enzymes that Aglianico and Sciascinoso grapes shared and lacked in Isabel and Tenta (Supplementary material Figure S3, panel A) did not appear to belong to any defined biosynthetic networks, probably due to the high number of functionally uncharacterized proteins. However, some of these enzymes are clearly involved in the metabolism of glutathione and in the synthesis of anthocyanins, such as an anthocyanidin synthase and a leucoanthocyanidin dioxygenase.

To infer possible traits of Isabel inherited by Sciascinoso, a profile expression analysis of enzymes in these two biotypes was carried out (Supplementary material Figure S3, panel B). Interestingly, in addition to cytochrome- and chlorophyll-binding proteins, several of these enzymes corresponded to hydrolases, but management of data including their similar levels of expression did not allow to deduce any biological significance associated with possible traits of the secondary metabolism.

Based on the analysis of expression profiles, Isabel and Tenta shared the expression of 29 enzymes (Supplementary material Figure S3, panel C), 4 of which were glycolytic enzymes, and at least additional 4 exhibited oxidoreductase activity. It was not possible to extrapolate enzymes potentially involved in the biosynthetic pathways of secondary metabolism common to Isabel and Tenta, also because many of the gene products remain functionally uncharacterized. However, the comparative analysis of the genetically uncharacterized Tenta grape, combined with the expression of specific metabolites, highlighted hybrid traits intermediate between Isabel and Aglianico, which are compatible with a (non-*V. vinifera* × *V. vinifera*) × *V. vinifera*) genotype.

To enlarge the functional classification of proteins from hybrid biotypes, the panel of differently expressed gene products was blasted against *Arabidopsis thaliana* using Blast2GO. However, the majority of the enzymes functionally assigned in this way belonged to pathways of primary metabolism, such as glycolysis, metabolism of dicarboxylic acids, amino acids and nucleotide, as well as citrate cycle and photosynthesis. In effects, the functional assignment through blasting with orthologous proteins of taxonomic divergent organisms, such as *A. thaliana*, may result in the underrepresentation of gene products involved in secondary metabolism pathways, which are specific to the *Vitis* genus or even species-specific [47].

The metabolic networks containing the highest number of gene products among the differential expressed proteins were visualized according to the Pathway Tools Omics Dashboard of the Plant Metabolic Network (https://pmn.plantcyc.org/ (accessed on 20 October 2021)) and are shown in Supplementary material Figure S4 [48]. The dashboard consists of multiple panels, each representing a system of cellular function (e.g., biosynthesis, degradation, energy), and a series of derived subsystems (e.g., glycolysis, photosynthesis, secondary metabolism). The large dot represents the average (mean) of all data values for gene products belonging to a given subsystem, while the small dots represent a data value for individual gene products within the subsystem. No substantial differences were apparent within the enzymes belonging to the secondary metabolism pathways, whereas significant differences appeared for proteins of the energy system, which could be induced by abiotic factors.

## 3. Materials and Methods

### 3.1. Materials

Solvents and chemicals of the highest commercially available purity were purchased from Sigma-Aldrich (St. Louis, MI, USA). Red grape samples were collected in different vineyards located in Irpinia, within the Campania, one of the regions of Southern Italy, during grape harvest 2016. Grapes varieties were genetically registered and certified by the Agronomic Inspectorate of Campania Region. Grape samples were from: (1) a purebred *Vitis vinifera* (Aglianico *cv.*) obtained from ancient grapevines grown in the wine district of Taurasi; (2) a *Vitis vinifera* scion from a cultivar locally known as Sciascinoso grafted onto an American *Vitis berlandieri* × *Vitis riparia* Kober 5BB rootstock; (3) a *Vitis vinifera* × *Vitis labrusca* interspecific hybrid (Isabel *cv*.); (4) an uncharacterized grape variety obtained from ungrafted old grapevines locally named “Tenta”, previously erroneously classified as a cognate of the red-fleshed Teinturier (*V. vinifera*) variety. Aglianico and Sciascinoso grapes sampled in this study are used to produce commercial wines. Approximately 1.5 kg of bunches for each grape variety were sampled at technical maturity, that is 20° Brix or higher assessed with a PCE-032 optical refractometer (PCE Instruments, Southampton, UK). Berries were carefully inspected to exclude visible diseases.

### 3.2. Anthocyanin Analysis

Anthocyanins from red grape skin were extracted with methanol/0.5% (*v*/*v*) 12N HCl for 6 h at room temperature. 3,5-*O*-diglucoside anthocyanins and their acylated (i.e., *p*-coumaroyl and caffeoyl) derivatives were monitored as species-specific markers of the hybrid grapes by MALDI-TOF MS. Before MS analysis, anthocyanins were purified by Zip-Tip (Millipore, Bedford, MA, USA) C18 reversed-phase pre-packed microcolumns, washing with 0.1% (*v*/*v*) trifluoroacetic acid (TFA) and eluting with 50% acetonitrile (*v*/*v*) containing 0.1% TFA.

MALDI-TOF MS analyses were carried out using a Voyager DE-Pro instrument (Perseptive Biosystem, Framingham, MA, USA) equipped with an N_2_ laser, using previously detailed conditions [8,27]. Spectra were acquired in the reflector positive ion mode (400–1200 *m*/*z* range) using sinapinic acid as the matrix, prepared by dissolving the crystalline powders (10 mg/mL) in 50% acetonitrile/0.1% TFA. Spectra were elaborated with the Data Explorer software 4.0 purchased with the instrument.

### 3.3. Purification/Extraction of Berry Grape Skin Proteins

To obtain nearly 50 g of grape skins for each variety, berries from at least five bunches were randomly detached and squished to separate flesh and seeds. After cutting the exocarp, the residual flesh was manually removed. Berry skins were accurately dried with filter paper, frozen in liquid nitrogen, finely ground with a mortar, and stored at −80 °C until use. Three replicate samples were prepared for each grape variety.

Proteins were extracted adapting the procedure by Negri et al. [22]. Briefly, aliquots (5 g) of powdered grape skin were twice homogenized with −20 °C cold acetone, and then centrifuged (5000× *g*, 30 min, 4 °C). The acetone powder was then resuspended in 20 mL of extraction buffer containing 0.7 M sucrose, 0.5 M Tris-HCl pH 8, 10 mM disodium EDTA salt, 0.4% (*v*/*v*) β-mercaptoethanol and a Halt^TM^ protease inhibitor cocktail (Pierce/Thermo Scientific, Rockford, IL, USA) under magnetic stirring in ice-cold bath for 30 min and then centrifuged at 13,000× *g* for 30 min. The supernatant was combined with an equal volume of Tris-buffered phenol (pH 8), stirred for 3 h in ice-cold bath, and finally centrifuged at 5000× *g* for 20 min at 4 °C to allow phase separation. The upper phenol phase was collected, while the bottom aqueous layer was back extracted with phenol, and the organic phases were pooled. Proteins were precipitated with five volumes of ice-cold 0.1 M ammonium acetate in methanol at −20 °C overnight. After centrifugation (10,000× *g*, 30 min, 4 °C) the protein precipitate was washed three times with cold acetone and finally air dried.

### 3.4. Reduction/Alkylation of Proteins and Tryptic Digestion

Aliquots of the protein acetonic powder (10 mg) were dissolved in 1 mL of a denaturing/reducing buffer containing 6 M guanidine HCl, 50 mM Tris, 1 mM EDTA, 10 mM DTT, pH 8.0 and incubated at 55 °C for 1 h under N_2_. After reduction, cysteine residues were alkylated with 55 mM iodoacetamide, for 40 min at room temperature in the dark. Proteins were purified by a size-exclusion chromatography spin desalting column with a 7-kDa size-exclusion limit (Pierce/Thermo Scientific) eluting with 0.5 M ammonium bicarbonate, pH 7.8, and quantified via a Bradford assay (kit from Bio-Rad, Milan, Italy). Purified proteins were sequentially digested with 1/100 sequencing grade Lys-C overnight at 37 °C, and 1/50 sequencing grade modified trypsin (enzymes from Promega, Madison, WI, USA) overnight at 37 °C. The resulting peptides were solid-phase extracted and purified from 0.3 mL of the solution with C18 Spin Columns (Thermo Scientifics, Rockford, IL, USA), by washing extensively with 0.1% (*v*/*v*) trifluoroacetic acid (TFA) and eluting with 70% acetonitrile/0.1% (*v*/*v*) TFA. Peptides were vacuum concentrated, then lyophilized and re-constituted in 0.1% formic acid for analysis.

### 3.5. Nanoflow-High Performance Liquid Chromatography-Electrospray Tandem Mass Spectrometry

Nanoflow-high performance liquid chromatography-electrospray tandem mass spectrometry (nHPLC−MS/MS) analyses were performed using an Ultimate 3000 nanoflow ultrahigh performance liquid chromatography (Dionex/Thermo Scientific, San Jose, CA, USA) coupled to a Q Exactive Orbitrap mass spectrometer (Thermo Scientific). For each sample, 2.5 μg of the peptide pool was loaded through Acclaim PepMap 100 trap columns (75 μm i.d. × 2 cm; Thermo Scientific) using a FAMOS autosampler (Thermo Scientific).

Eluent A was 0.1% formic acid (*v*/*v*) in LC–MS-grade water; eluent B was 0.1% formic acid (*v*/*v*) in 80% aqueous acetonitrile. Peptides were separated using an EASY-Spray™ PepMap C18 column (15 cm × 75 μm i.d.) with 2-μm particles and 100-Å pore size (Thermo Scientific). Peptides were separated applying a 2–50% gradient of B over 120 min after 10 min of isocratic elution at 2% B, at a constant flow rate of 300 nL/min. MS1 precursor spectra were acquired in the positive ionization mode scanning the 1600–350 *m*/*z* range with resolving power of 70,000 full width at half maximum (FWHM), an automatic gain control (AGC) target of 1 × 10^6^ ions, and maximum ion injection time of 100 ms. The spectrometer operated in full scan MS1 and data-dependent acquisition mode, selecting up to the 10 most intense ions for MS/MS fragmentation and applying an 8 s dynamic exclusion. Fragmentation spectra were obtained at a resolving power of 17,500 FWHM. Ions with charge +1 or greater than +6 were excluded from the MS/MS fragmentation. Spectra were elaborated using the software Xcalibur version 3.1 (Thermo Scientific).

### 3.6. Database Search and Protein Identification

Raw files of the nHPLC−MS/MS runs were used as the output for the protein identification using the Andromeda search engine of the MaxQuant bioinformatic suite against the *Vitis* spp. Uniprot database downloaded in July 2020. For all the searches, parameters were the following: mass tolerance value 10 ppm for the precursor and 0.02 for the fragment ions, trypsin as the proteolytic enzyme, Cys-carbamidomethylation as a static modification; Met oxidation, pyroglutamic acid at N-terminus Gln, Ser/Thr phosphorylation as variable modifications. Peptide spectrum matches (PSMs) were filtered using the target decoy database approach with an e value of 0.01 peptide-level false discovery rate (FDR), corresponding to a 99% confidence score. Technical replicates (*n* = 3) were merged and considered as a unique experiment set.

### 3.7. Bioinformatics and Functional Classification

Statistical analysis of the proteomic data was performed with Perseus v1.6.10.0. Normalized label free quantification intensity values were log_2_ transformed to render the data normally distributed. Proteins identified by site reverse, and potential contaminants were filtered prior to analysis. Entries with missing values in two out of three replicates were filtered.

Protein functional categorization according to the Bevan classification [49] and assignment to biosynthetic pathways was accomplished with dedicated analysis searching the UniprotKB and KEGG (Kyoto Encyclopedia of Genes and Genomes) pathway databases. Using the *Vitis* genus UniprotKB database, a relatively high number of proteins were annotated as putative uncharacterized proteins [50]. To enlarge the functional annotation, the differential expressed proteins were blasted against the *Arabidopsis thaliana* database using the Blast2GO software (Bioinformatics Department, CIPF, Valencia, Spain). Functional classification of genes coding for the identified protein entries was carried out also with the PANTHER classification system (release 2020) in Gene Ontology (http://geneontology.org/ (accessed on 15 September 2021)) and VitisCyc in the Plant Metabolic Network platform 15.0 release (https://plantcyc.org/ (accessed on 20 October 2020)) [47]. Potential protein-protein interaction (PPI) networks among identified genes were established using the STRING v11.0 (https://string-db.org (accessed on 15 September 2021)) open source.

## 4. Conclusions

In this study, for the first time to our knowledge, the protein expressions of berry skin by four different red grape biotypes with variable degrees of hybrid traits have been compared at proteome-wide level.

Proteomics supported several metabolic divergences between Isabel (*V. vinifera × V. labrusca*) and Aglianico (purebred *V. vinifera*) grapes on a phenotypic basis. Due to the incompleteness of the protein annotation and functional classification of grape varieties, especially of the non-*V. vinifera* biotypes [51], the majority of the classified proteins with different expression profiles belonged to coordinated biosynthetic networks of primary metabolism (glycolysis, oxidative phosphorylation, cell redox enzymatic systems, gene expression). In contrast, the connections within biosynthetic pathways of secondary metabolites were limited and fragmentary. The PCA analysis demonstrated a strict parentage between *V. vinifera* scion grafted onto an interspecific American hybrid (*V. berlandieri × V. riparia*) rootstock, namely Sciascinoso grape, and purebred *V. vinifera*, as well as a substantial divergence with Isabel. However, the proteome of Sciascinoso grape exhibited some inherited “wild” characters distinct from those of purebred *V. vinifera*, although a more detailed study of the metabolic pathways involved would be needed.

Proteomics disclosed a striking complexity of the gene regulation processes, consistent with previous genomic and transcriptomic data. The proteome profile of grape skin could represent an irreplaceable tool complementary to genomics for establishing the purity of indigenous registered varieties of *V. vinifera* grapevine. Clearly, results of this study are preliminary and emphasize the concept that proteomics has lagged behind grape transcriptomics, genomics and metabolomics. However, the issue deserves dedicated proteomics analyses extended to other vine and berry tissues, to multiple physiological stages of the life cycle and to varying grape biotypes to ultimately delineate the dynamic regulation of grape metabolism, through integration with other “-omics” in a system biology perspective.

## Data Availability

The mass spectrometry proteomics data have been deposited to the ProteomeXchange Consortium via the PRIDE [52] partner repository with the dataset identifier PXD025784. For the review purpose, data can be accessed with the following account details: Username: reviewer_pxd025784@ebi.ac.uk. Password: ztbD4t1J.

## Supplementary Materials

The following supporting information can be downloaded at: https://www.mdpi.com/article/10.3390/ijms23031051/s1.

## References

[1] Macedo M. (2011). Port Wine Landscape: Railroads, Phylloxera, and Agricultural Science. Agric. Hist..

[2] Tello J., Mammerler R., Čajić M., Forneck A. (2019). Major Outbreaks in the Nineteenth Century Shaped Grape Phylloxera Contemporary Genetic Structure in Europe. Sci. Rep..

[3] Sun Q., Gates M.J., Lavin E.H., Acree T.E., Sacks G.L. (2011). Comparison of Odor-Active Compounds in Grapes and Wines from *Vitis vinifera* and Non-Foxy American Grape Species. J. Agric. Food Chem..

[4] Gale G., Sandler M., Pindler R. (2003). Saving the vine from phylloxera: A never ending battle. Wine: A Scientific Exploration.

[5] Narduzzi L., Stanstrup J., Mattivi F. (2015). Comparing Wild American Grapes with *Vitis vinifera*: A Metabolomics Study of Grape Composition. J. Agric. Food Chem..

[6] European Community (CE) Regulation No. 479/2008 Art. 24, Par 1. https://eur-lex.europa.eu/eli/reg/2008/479/oj.

[7] Mazzuca P., Ferranti P., Picariello G., Chianese L., Addeo F. (2005). Mass spectrometry in the study of anthocyanins and their derivatives: Differentiation of *Vitis vinifera* and hybrid grapes by liquid chromatography/electrospray ionization mass spectrometry and tandem mass spectrometry. Biol. Mass Spectrom..

[8] Picariello G., Ferranti P., Garro G., Manganiello G., Chianese L., Coppola R., Addeo F. (2014). Profiling of anthocyanins for the taxonomic assessment of ancient purebred V. vinifera red grape varieties. Food Chem..

[9] Flamini R., Tomasi D. (2009). The anthocyanin content in berries of the hybrid grape cultivars Clinton and Isabella. Vitis.

[10] Jánváry L., Hoffmann T., Pfeiffer J., Hausmann L., Töpfer R., Fischer T.C., Schwab W. (2009). A Double Mutation in the Anthocyanin 5-O-Glucosyltransferase Gene Disrupts Enzymatic Activity in *Vitis vinifera* L.. J. Agric. Food Chem..

[11] Yang Y., Labate J.A., Liang Z., Cousins P., Prins B., Preece J.E., Aradhya M., Zhong G.-Y. (2014). Multiple loss-of-function 5-O-glucosyltransferase alleles revealed in *Vitis vinifera*, but not in other Vitis species. Theor. Appl. Genet..

[12] Xing R.-R., Li S.-Y., He F., Yang Z., Duan C.-Q., Li Z., Wang J., Pan Q.-H. (2015). Mass Spectrometric and Enzymatic Evidence Confirm the Existence of Anthocyanidin 3,5-O-Diglucosides in Cabernet Sauvignon (*Vitis vinifera* L.) Grape Berries. J. Agric. Food Chem..

[13] Gautier A., Cookson S.J., Lagalle L., Ollat N., Marguerit E. (2020). Influence of the three main genetic backgrounds of grapevine rootstocks on petiolar nutrient concentrations of the scion, with a focus on phosphorus. OENO One.

[14] Zhang Z., Liu H., Sun J., Yu S., He W., Li T., Baolong Z. (2020). Nontarget Metabolomics of Grape Seed Metabolites Produced by Various Scion–Rootstock Combinations. J. Am. Soc. Hortic. Sci..

[15] Chitarra W., Perrone I., Avanzato C.G., Minio A., Boccacci P., Santini D., Gilardi G., Siciliano I., Gullino M.L., Delledonne M. (2017). Grapevine Grafting: Scion Transcript Profiling and Defense-Related Metabolites Induced by Rootstocks. Front. Plant Sci..

[16] Zombardo A., Crosatti C., Bagnaresi P., Bassolino L., Reshef N., Puccioni S., Faccioli P., Tafuri A., Delledonne M., Fait A. (2020). Transcriptomic and biochemical investigations support the role of rootstock-scion interaction in grapevine berry quality. BMC Genom..

[17] Ollat N., Bordenave L., Tandonnet J., Boursiquot J., Marguerit E. (2016). Grapevine rootstocks: Origins and perspectives. Acta Hortic..

[18] Jaillon O., Aury J.M., Noel B., Policriti A., Clepet C., Casagrande A., Choisne N., Aubourg S., Vitulo N., Jubin C. (2007). The French–Italian Public Consortium for Grapevine Genome Characterization. The grapevine genome sequence suggests ancestral hexaploidization in major angiosperm phyla. Nature.

[19] Sweetman C., Wong D.C., Ford C.M., Drew D.P. (2012). Transcriptome analysis at four developmental stages of grape berry (*Vitis vinifera* cv. *Shiraz*) provides insights into regulated and coordinated gene expression. BMC Genom..

[20] Leng X., Wang P., Wang C., Zhu X., Li X., Li H., Mu Q., Li A., Liu Z., Fang J. (2017). Genome-wide identification and characterization of genes involved in carotenoid metabolic in three stages of grapevine fruit development. Sci. Rep..

[21] Zhou Y., Massonnet M., Sanjak J.S., Cantu D., Gaut B.S. (2017). Evolutionary genomics of grape (*Vitis viniferas* sp. vinifera) domestication. Proc. Natl. Acad. Sci. USA.

[22] Negri A.S., Prinsi B., Rossoni M., Failla O., Scienza A., Cocucci M., Espen L. (2008). Proteome changes in the skin of the grape cultivar Barbera among different stages of ripening. BMC Genom..

[23] Negri A.S., Prinsi B., Failla O., Scienza A., Espen L. (2015). Proteomic and metabolic traits of grape exocarp to explain different anthocyanin concentrations of the cultivars. Front. Plant Sci..

[24] Niu N., Wu B., Yang P., Li S. (2013). Comparative analysis of the dynamic proteomic profiles in berry skin between red and white grapes (*Vitis vinifera* L.) during fruit coloration. Sci. Hortic..

[25] Niu N., Cao Y., Duan W., Wu B., Li S. (2013). Proteomic analysis of grape berry skin responding to sunlight exclusion. J. Plant Physiol..

[26] Ghan R., Van Sluyter S.C., Hochberg U., Degu A., Hopper D.W., Tillet R.L., Schlauch K.A., Haynes P.A., Fait A., Cramer G.R. (2015). Five omic technologies are concordant in differentiating the biochemical characteristics of the berries of five grapevine (*Vitis vinifera* L.) cultivars. BMC Genom..

[27] Picariello G., Ferranti P., Chianese L., Addeo F. (2012). Differentiation of *Vitis vinifera* L. and Hybrid Red Grapes by Matrix-Assisted Laser Desorption/Ionization Mass Spectrometry Analysis of Berry Skin Anthocyanins. J. Agric. Food Chem..

[28] Grimplet J., Deluc L.G., Tillett R.L., Wheatley M.D., Schlauch K.A., Cramer G.R., Cushman J.C. (2007). Tissue-specific mRNA expression profiling in grape berry tissues. BMC Genom..

[29] Aguilan J.T., Kulej K., Sidoli S. (2020). Guide for protein fold change and p-value calculation for non-experts in proteomics. Mol. Omics.

[30] Wang J., De Luca V. (2005). The biosynthesis and regulation of biosynthesis of Concord grape fruit esters, including ‘foxy’ methylanthranilate. Plant J..

[31] Hall D., Yuan X.X., Murata J., De Luca V. (2011). Molecular cloning and biochemical characterization of the UDP-glucose: Flavonoid 3-O-glucosyltransferase from Concord grape (*Vitis labrusca*). Phytochemistry.

[32] He F., Chen W.-K., Yu K.-J., Ji X.-N., Duan C.-Q., Reeves M.J., Wang J. (2015). Molecular and biochemical characterization of the UDP-glucose: Anthocyanin 5-O-glucosyltransferase from *Vitis amurensis*. Phytochemistry.

[33] Hall D., De Luca V. (2007). Mesocarp localization of a bi-functional resveratrol/hydroxycinnamic acid glucosyltransferase of Concord grape (*Vitis labrusca*). Plant J..

[34] Sun L., Fan X., Zhang Y., Jiang J., Sun H., Liu C. (2016). Transcriptome analysis of genes involved in anthocyanins biosynthesis and transport in berries of black and white spine grapes (*Vitis davidii*). Hereditas.

[35] Serrano A., Espinoza C., Armijo G., Inostroza-Blancheteau C., Poblete E., Meyer-Regueiro C., Arce A., Parada F., Santibáñez C., Arce-Johnson P. (2017). Omics Approaches for Understanding Grapevine Berry Development: Regulatory Networks Associated with Endogenous Processes and Environmental Responses. Front. Plant Sci..

[36] Yan X., Qiao H., Zhang X., Guo C., Wang M., Wang Y., Wang X. (2017). Analysis of the grape (*Vitis vinifera* L.) thaumatin-like protein (TLP) gene family and demonstration that TLP29 contributes to disease resistance. Sci. Rep..

[37] Burbidge C.A., Ford C.M., Melino V.J., Wong D.C.J., Jia Y., Jenkins C.L.D., Soole K.L., Castellarin S.D., Darriet P., Rienth M. (2021). Biosynthesis and Cellular Functions of Tartaric Acid in Grapevines. Front. Plant Sci..

[38] Conn S., Curtin C., Bézier A., Franco C., Zhang W. (2008). Purification, molecular cloning, and characterization of glutathione S-transferases (GSTs) from pigmented *Vitis vinifera* L. cell suspension cultures as putative anthocyanin transport proteins. J. Exp. Bot..

[39] Sartor S., Burin V.M., Ferreira-Lima N.E., Caliari V., Bordignon-Luiz M.T. (2019). Polyphenolic Profiling, Browning, and Glutathione Content of Sparkling Wines Produced with Nontraditional Grape Varieties: Indicator of Quality During the Biological Aging. J. Food Sci..

[40] Melino V.J., Soole K.L., Ford C.M. (2009). Ascorbate metabolism and the developmental demand for tartaric and oxalic acids in ripening grape berries. BMC Plant Biol..

[41] Liu H.-F., Wu B.-H., Fan P.-G., Xu H.-Y., Li S.-H. (2006). Inheritance of sugars and acids in berries of grape (*Vitis vinifera* L.). Euphytica.

[42] Teissedre P.-L. (2018). Composition of grape and wine from resistant vines varieties. OENO One.

[43] Pedastsaar P., Vaher M., Helmja K., Kulp M., Kaljurand M., Karp K., Raal A., Karathanos V., Püssa T. (2014). Chemical composition of red wines made from hybrid grape and common grape (*Vitis vinifera* L.) cultivars. Proc. Est. Acad. Sci..

[44] Fournier-Level A., Hugueney P., Verriès C., This P., Ageorges A. (2011). Genetic mechanisms underlying the methylation level of anthocyanins in grape (*Vitis vinifera* L.). BMC Plant Biol..

[45] Ageorges A., Fernandez L., Vialet S., Merdinoglu D., Terrier N., Romieu C. (2006). Four specific isogenes of the anthocyanin metabolic pathway are systematically co-expressed with the red colour of grape berries. Plant Sci..

[46] Hiratsuka S., Onodera H., Kawai Y., Kubo T., Itoh H., Wada R. (2001). Enzyme activity changes during anthocyanin synthesis in ‘Olympia’ grape berries. Sci. Hortic..

[47] López-Hidalgo C., Guerrero-Sánchez V.M., Gómez-Gálvez I., Sánchez-Lucas R., Castillejo-Sánchez M.A., Alconada A.M.M., Valledor L., Jorrín-Novo J.V. (2018). A Multi-Omics Analysis Pipeline for the Metabolic Pathway Reconstruction in the Orphan Species Quercus ilex. Front. Plant Sci..

[48] Naithani S., Raja R., Waddell E.N., Elser J., Gouthu S., Deluc L.G., Jaiswal P. (2014). VitisCyc: A metabolic pathway knowledgebase for grapevine (*Vitis vinifera*). Front. Plant Sci..

[49] Project T.E.A.G., Bevan M., Bancroft I., Bent E., Love K., Goodman H., Dean C., Bergkamp R., Dirkse W., Van Staveren M. (1998). Analysis of 1.9 Mb of contiguous sequence from chromosome 4 of Arabidopsis thaliana. Nature.

[50] Kuang L., Chen S., Guo Y., Ma H. (2019). Quantitative Proteome Analysis Reveals Changes in the Protein Landscape During Grape Berry Development with a Focus on Vacuolar Transport Proteins. Front. Plant Sci..

[51] Massonnet M., Fasoli M., Tornielli G.B., Altieri M., Sandri M., Zuccolotto P., Paci P., Gardiman M., Zenoni S., Pezzotti M. (2017). Ripening Transcriptomic Program in Red and White Grapevine Varieties Correlates with Berry Skin Anthocyanin Accumulation. Plant Physiol..

[52] Perez-Riverol Y., Csordas A., Bai J., Bernal-Llinares M., Hewapathirana S., Kundu D.J., Inuganti A., Griss J., Mayer G., Eisenacher M. (2019). The PRIDE database and related tools and resources in 2019: Improving support for quantification data. Nucleic Acids Res..

