# Supplementary material for: Differential Protein Expression in Berry Skin from Red Grapes with Varying Hybrid Character
Source: Int J Mol Sci. 2022 Jan 19;23(3):1051. doi: 10.3390/ijms23031051 (PMC8835309; doi:10.3390/ijms23031051)
Supplement: Supplementary file 1 [file ijms-23-01051-s001.zip › ijms-1543460-supplementary.pdf]

Supporting Information

Differential protein expression in berry skin from red grapes with varying hybrid character

Valentina Spada[1], Luigia Di Stasio[1], Pasquale Ferranti[1,2], Francesco Addeo[2,#], Gianfranco Mamone[1,*], Gianluca Picariello[1,*]

[1]Istituto di Scienze dell'Alimentazione – Consiglio Nazionale delle Ricerche (CNR), Via Roma 64, I-83100 Avellino, Italy

[2]Dipartimento di Agraria, Università di Napoli "Federico II", Parco Gussone, Portici, Napoli I-80055 Italy

*** Correspondence:**

Corresponding Authors

mamone@isa.cnr.it, picariello@isa.cnr.it

[#] retired. Former affiliation indicated.

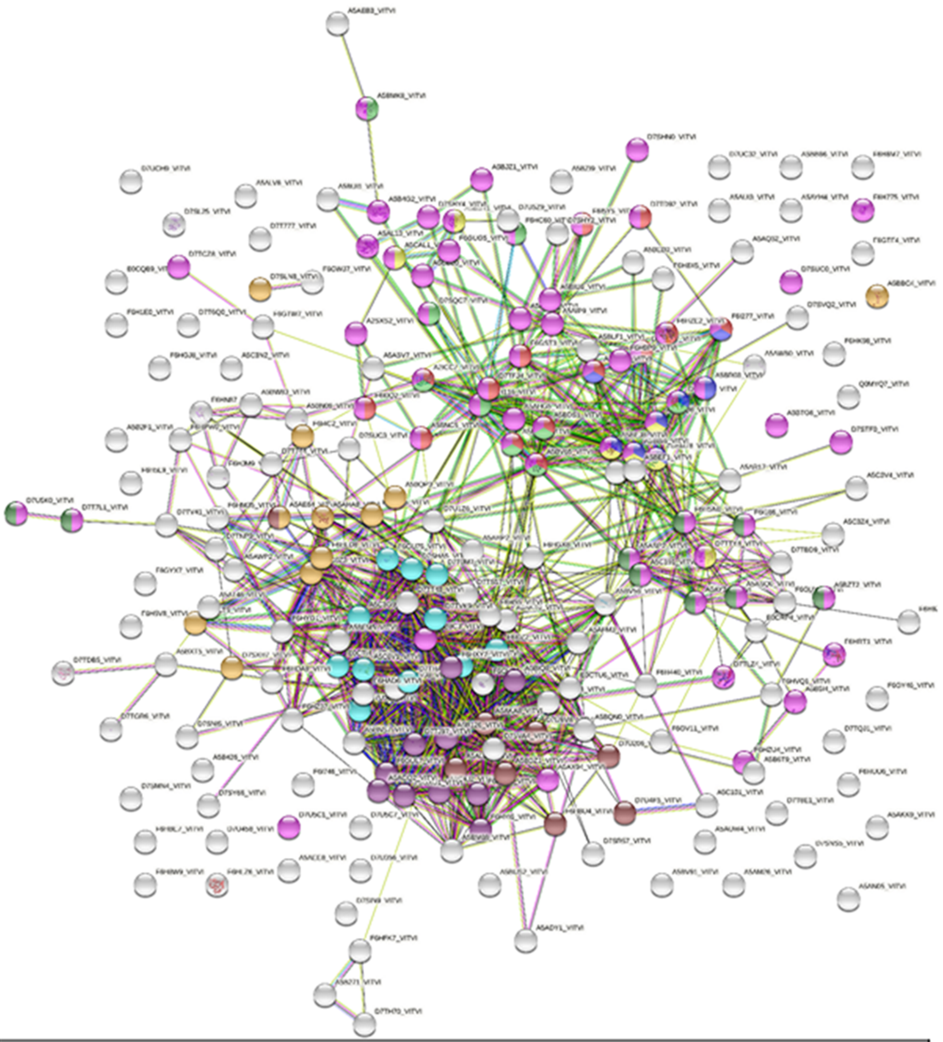

KEGG Pathways				
Pathway	Description	Count of network	False discovery rate	
vvi01230	Biosynthesis of amino acids	14 of 211	1.64e-08	
vvi00020	Citrate cycle (TCA cycle)	8 of 49	9.54e-08	
vvi00010	Glycolysis/Gluconeogenesis	9 of 119	3.28e-06	
vvi00630	Glyoxylate and dicarboxylate methabolism	7 of 69	7.45e-06	
vvi01100	Metabolic pathways	61 of 1867	1.17e-21	
vvi00190	Oxidative phosphorylation	9 of 125	3.96e-06	
vvi03050	Proteasome	11 of 48	7.94e-12	
vvi04141	Protein processing in endoplasmatic reticulum	11 of 202	3.41e-06	
vvv03010	Ribosome	10 of 257	0.00013	
vvi03013	RNA transport	8 of 146	8.90e-05	

Figure S1. PPI network complex of proteins with comparable expression levels (255 entries), containing 215 nodes and 1011 edges. Ten principal functional groups in terms of number of protein members involved are labelled according to the KEGG classification.

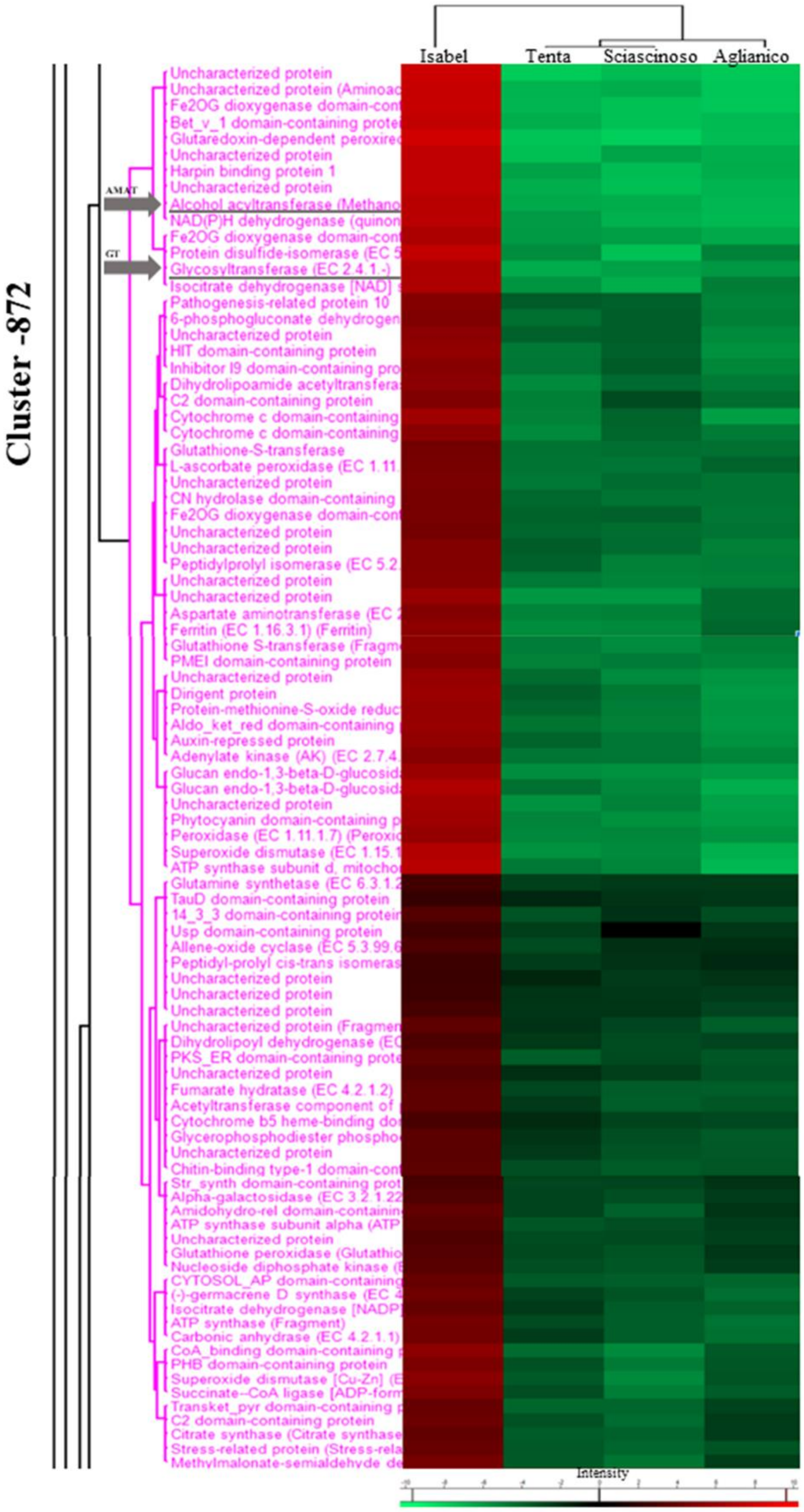

Figure S2. Details of the heat map relevant to hierarchical cluster analysis of the four samples, highlighting the clustering of gene products up-regulated in Isabel (cluster id number "-872"). Proteins indicated with an arrow are AMAT (methanol O-anthraniloyltransferase) and the glycotransferase Q0PI14 discussed in the text.

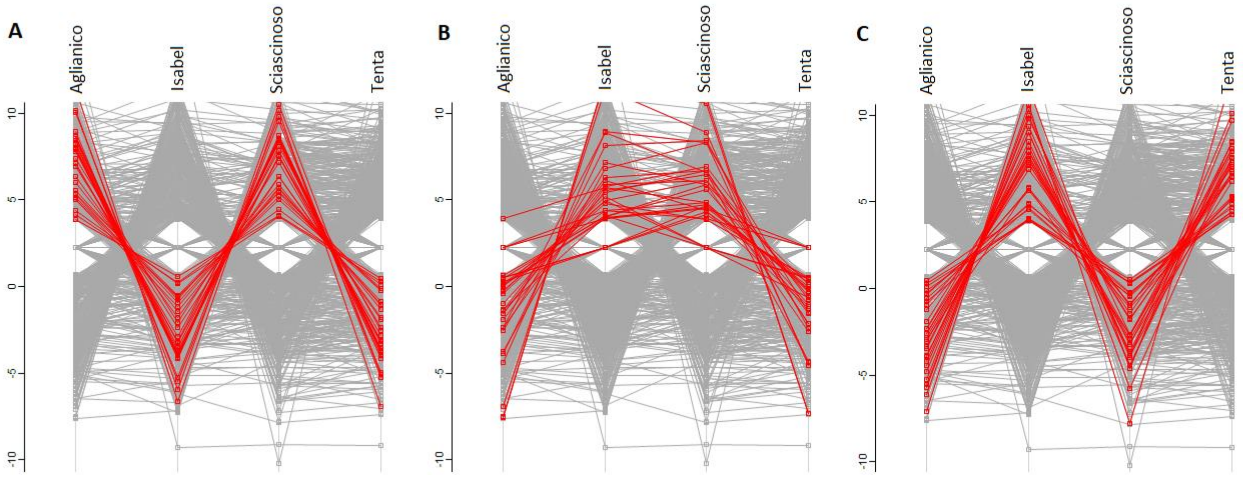

Figure S3. Profiles of proteins with comparable degree of expression between couples of grape biotypes. To assess the phenotypical similarity of purebred *V. vinifera* and the grafted *V. vinifera* scion, and to infer the hybrid character transferred from the rootstock to the scion, pairwise profiles of comparable expression levels were extrapolated for Aglianico and Sciascinoso (A) and Isabel and Sciascinoso (B) grapes, respectively. Profiles of comparable expression levels of Isabel and Tenta were extrapolated to assess the hybrid character of Tenta grape (C).

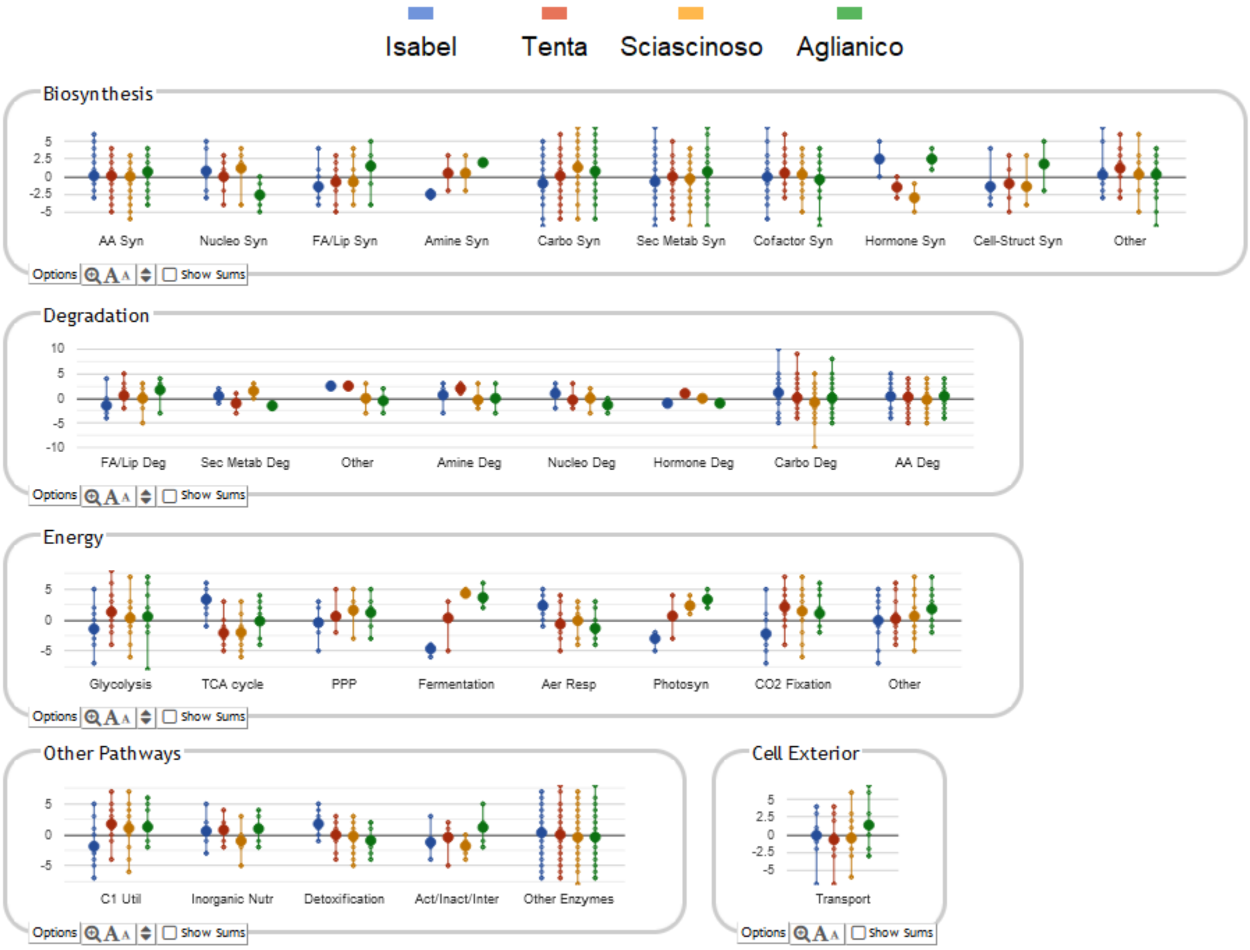

Figure S4. The most represented metabolic networks in terms of number of gene products among the differential expressed proteins were visualized according to the Pathway Tools Omics Dashboard of the Plant Metabolic Network (https://pmn.plantcyc.org/). The dashboard contains multiple panels, each representing a system of cellular function and a series of derived subsystems. The large dot represents the average (mean) of all data values for gene products belonging to a given subsystem, while the small dots represent a data value for individual gene products within the subsystem. Fluctuation of gene products belonging to given subsystems can be appreciated by the intensity (log_2) of the y-axis.

Table S1. List of 892 proteins identified in the four wine types using proteomics with differentially expression and with a fold-change threshold of 1.5. The left column indicated the cluster number of hierarchical analysis.

N/A=Not Available.

Cluster n°	Isabel (log2 fold-change)	Tenta (log2 fold-change)	Sciascinoso (log2 fold-change)	Aglianico (log2 fold-change)	Organism	Protein name	Gene name	UniProt name	Peptides	Sequence coverage [%]	Mol. weight [kDa]	Score	Intensity
**Main Clusters**													
-797	-5,80	-6,04	-5,68	6,04	Vitis vinifera	Putative thaumatin-like protein (Fragment)	Tl2	Q9M4G7_VITVI	8	42,10	20,12	13,97	1,20E+09
-797	-9,55	-9,42	-9,37	9,55	Vitis vinifera	Uncharacterized protein;VVTL1	VIT_02s0025g04330	F6HUH1_VITVI	13	50,50	23,97	323,31	1,42E+10
-797	-6,56	-6,50	-6,45	6,56	Vitis vinifera	Non-specific lipid-transfer protein	VIT_08s0058g01230	F6GXX3_VITVI	3	38,30	10,52	23,41	1,35E+09
-797	-6,22	-6,08	-4,33	6,22	Vitis labrusca x Vitis vinifera	Glycosyltransferase (EC 2.4.1.-)	VIT_00s0324g00050	A5B177_VITVI	8	23,70	54,60	39,33	5,25E+08
-797	-6,94	-7,97	-8,36	8,36	Vitis vinifera	Uncharacterized protein	VIT_14s0068g01760	A5BTT7_VITVI	8	42,50	29,39	76,27	4,63E+08
-872	7,69	-7,69	-7,10	-7,60	Vitis vinifera	Uncharacterized protein	VIT_10s0071g00810	F6HVY8_VITVI	13	37,10	47,99	54,61	1,09E+09
-872	7,45	-6,79	-6,56	-7,45	Vitis vinifera	Uncharacterized proteincy	VIT_18s0001g03230	F6H0T0_VITVI	4	13,80	50,29	8,13	2,87E+08
-872	7,52	-6,87	-7,33	-7,52	Vitis vinifera	Fe2OG dioxygenase domain-containing protein	VIT_03s0063g01290	D7TPR3_VITVI	10	40,10	39,26	127,18	1,89E+08
-872	7,32	-6,59	-7,32	-7,02	Vitis vinifera	Bet_v_1 domain-containing protein	VIT_01s0011g05150	D7T8R2_VITVI	4	40,60	15,83	16,07	5,16E+08
-872	7,87	-7,39	-7,87	-7,13	Vitis vinifera	Glutaredoxin-dependent peroxiredoxin (EC 1.11.1.25)	VIT_05s0020g02850	D7T6T0_VITVI	6	38,30	21,83	15,17	1,06E+09
-872	7,25	-7,25	-6,19	-6,60	Vitis vinifera	Uncharacterized protein	VIT_02s0025g04290	A5C9F2_VITVI	2	13,80	24,06	79,29	3,70E+08
-872	7,16	-6,14	-7,16	-6,53	Vitis vinifera	PAP_fibrillin domain-containing protein	VIT_05s0049g01360	D7SZJ5_VITVI	7	39,20	31,38	69,38	1,00E+09
-872	7,26	-6,57	-7,26	-6,81	Vitis vinifera	Uncharacterized protein	VIT_04s0008g02920	D7SU58_VITVI	4	20,50	32,02	25,44	3,68E+08
-872	6,94	-6,43	-6,52	-6,94	Vitis labrusca (Concord grape)	Alcohol acyltransferase;Methanol O-anthraniloyltransferase (EC 2.3.1.232) (Anthraniloyl-CoA:methanol acyltransferase) (Benzyl alcohol O-benzoyltransferase) (EC 2.3.1.196)	AMAT	ACMAT_VITLA	14	40,10	50,15	102,40	1,37E+09
-872	6,99	-5,86	-6,74	-6,99	Vitis vinifera	NAD(P)H dehydrogenase (quinone) (EC 1.6.5.2)	Qor	Q6YCG4_VITVI	7	51,20	21,74	140,29	2,98E+09
-872	6,42	-5,81	-6,02	-6,42	Vitis vinifera	Fe2OG dioxygenase domain-containing protein	VIT_11s0118g00370	F6HCH1_VITVI	7	19,60	36,27	23,44	3,70E+08
-872	7,29	-5,32	-7,29	-4,93	Vitis vinifera	Protein disulfide-isomerase (EC 5.3.4.1)	VIT_18s0001g02510	E0CR49_VITVI	31	70,30	55,67	323,31	1,84E+10
-872	6,50	-6,50	-6,08	-5,72	Vitis labrusca	Glycosyltransferase (EC 2.4.1.-)	VIT_03s0091g00040	Q0PI14_VITLA	9	24,00	53,83	109,65	4,16E+08
-872	6,57	-5,62	-6,57	-4,76	Vitis vinifera	Isocitrate dehydrogenase [NAD] subunit, mitochondrial	VIT_08s0040g01700	D7TQM9_VITVI	11	37,40	40,18	240,20	2,63E+09
-872	5,00	-3,50	-3,65	-5,00	Vitis hybrid cultivar	Pathogenesis-related protein 10	Tam-RP10	A9CSL9_9ROSI	8	67,10	17,13	188,63	2,22E+10
-872	4,82	-4,23	-3,58	-4,82	Vitis vinifera	6-phosphogluconate dehydrogenase, decarboxylating (EC 1.1.1.44)	VITISV_032572	A5B7A4_VITVI	22	55,40	55,29	235,55	4,76E+09
-872	5,28	-3,67	-3,54	-5,28	Vitis vinifera	Uncharacterized protein	VIT_13s0064g00970	D7T300_VITVI	12	48,60	27,53	70,04	2,15E+09
-872	5,49	-4,56	-3,66	-5,49	Vitis vinifera	HIT domain-containing protein	VITISV_029944	A5BDT4_VITVI	6	44,40	17,32	48,58	1,46E+09
-872	5,12	-4,50	-3,46	-5,12	Vitis vinifera	Inhibitor I9 domain-containing protein	VIT_18s0117g00130	A5BSE8_VITVI	4	39,80	11,40	16,27	3,98E+08
-872	5,26	-5,26	-4,04	-4,64	Vitis vinifera	Dihydrolipoamide acetyltransferase component of pyruvate dehydrogenase complex (EC 2.3.1.-)	VITISV_023192	A5BQI0_VITVI	10	26,00	51,08	30,63	1,84E+09
-872	4,84	-4,84	-2,84	-4,15	Vitis vinifera	C2 domain-containing protein	VITISV_006805	A5C8Y4_VITVI	7	42,70	30,78	121,76	9,46E+08
-872	6,00	-4,95	-3,77	-6,00	Vitis vinifera	Cytochrome c domain-containing protein	VITISV_033958	A5AQD0_VITVI	3	28,60	12,18	9,13	3,34E+08
-872	5,22	-5,22	-3,92	-4,69	Vitis vinifera	Cytochrome c domain-containing protein	VIT_09s0002g05950	D7U0V9_VITVI	6	27,80	34,77	104,13	3,59E+08
-872	4,35	-4,35	-4,19	-4,30	Vitis vinifera	Glutathione-S-transferase	*N/A*	Q84N22_VITVI	9	37,90	15,65	3,07	2,63E+08
-872	4,45	-4,38	-4,45	-3,80	Vitis pseudoreticulata (Chinese wild grapevine)	L-ascorbate peroxidase (EC 1.11.1.11)	APX	Q1AFF4_9ROSI	12	64,40	27,62	6,51	7,49E+07
-872	4,57	-4,57	-4,09	-4,41	Vitis vinifera	Uncharacterized protein	VIT_14s0060g00900	F6I4U2_VITVI	3	21,70	18,73	6,49	1,93E+08
-872	4,43	-4,00	-4,43	-4,38	Vitis vinifera	CN hydrolase domain-containing protein	VIT_06s0080g00940	F6HHD4_VITVI	3	15,60	37,63	7,61	3,62E+08
-872	4,52	-3,74	-3,65	-4,52	Vitis vinifera	Fe2OG dioxygenase domain-containing protein	VIT_01s0011g05650	F6HFE2_VITVI	3	10,40	43,77	4,51	5,60E+07
-872	4,38	-3,90	-4,16	-4,38	Vitis vinifera	Uncharacterized protein	VIT_17s0000g02140	F6GSN7_VITVI	6	5,70	141,60	10,27	2,03E+08
-872	4,85	-3,51	-4,08	-4,85	Vitis vinifera	Uncharacterized protein	VIT_07s0031g01190	D7SW22_VITVI	9	21,00	72,65	20,40	4,74E+08
-872	4,89	-3,70	-4,89	-4,72	Vitis vinifera	Peptidylprolyl isomerase (EC 5.2.1.8)	VIT_03s0017g01060	D7TU83_VITVI	4	46,40	12,00	14,04	4,78E+08

Cluster n°	Isabel (log2 fold-change)	Tenta (log2 fold-change)	Sciascinoso (log2 fold-change)	Aglianico (log2 fold-change)	Organism	Protein name	Gene name	UniProt name	Peptides	Sequence coverage [%]	Mol. weight [kDa]	Score	Intensity
-872	4,96	-4,65	-4,96	-4,89	Vitis vinifera	Uncharacterized protein	VIT_06s0004g01310	F6GUE3_VITVI	7	23,10	53,83	51,81	7,35E+08
-872	5,74	-5,74	-5,74	-4,13	Vitis vinifera	Uncharacterized protein	VIT_12s0057g01200	F6HHQ7_VITVI	19	68,20	42,34	323,31	9,24E+09
-872	4,99	-4,99	-4,89	-4,13	Vitis vinifera	Aspartate aminotransferase (EC 2.6.1.1)	VIT_12s0028g01820	E0CTU3_VITVI	7	22,70	47,30	19,34	4,47E+08
-872	5,31	-5,31	-5,24	-3,87	Vitis vinifera	Ferritin (EC 1.16.3.1)	VIT_08s0058g00440	F6GXT8_VITVI	5	24,10	29,25	9,85	2,42E+08
-872	5,30	-4,76	-5,30	-4,75	Vitis vinifera	Glutathione S-transferase (Fragment)	GST3	A4LAG9_VITVI	3	19,50	24,97	3,59	3,47E+07
-872	5,00	-4,92	-4,69	-5,00	Vitis vinifera	PMEI domain-containing protein	VIT_00s0323g00070	A5CBR6_VITVI	2	17,60	21,89	12,13	1,44E+08
-872	5,62	-4,12	-5,31	-5,62	Vitis vinifera	Uncharacterized protein	VIT_13s0067g03800	F6HC13_VITVI	5	40,00	26,91	204,90	9,76E+08
-872	5,83	-3,61	-4,55	-5,83	Vitis vinifera	Dirigent protein	VIT_06s0004g00980	F6GUJ0_VITVI	2	11,30	21,97	1,98	4,46E+07
-872	5,64	-3,91	-4,75	-5,64	Vitis vinifera	Protein-methionine-S-oxide reductase (EC 1.8.4.11)	VIT_03s0088g00730	F6HBN5_VITVI	4	25,80	21,33	5,56	3,59E+08
-872	5,73	-4,41	-4,87	-5,73	Vitis vinifera	Aldo_ket_red domain-containing protein	VIT_14s0030g02020	D7TUK1_VITVI	12	47,80	38,50	15,21	8,71E+07
-872	5,52	-3,81	-4,50	-5,52	Vitis vinifera	Auxin-repressed protein	ARP1	A5BEH3_VITVI	4	43,00	13,46	27,60	6,78E+08
-872	5,08	-4,44	-4,52	-5,08	Vitis vinifera	Adenylate kinase (AK) (EC 2.7.4.3) (ATP-AMP transphosphorylase) (ATP:AMP phosphotransferase) (Adenylate monophosphate kinase)	VIT_17s0000g01730	D7SGV3_VITVI	7	34,70	26,97	80,93	1,45E+09
-872	5,80	-5,35	-5,51	-5,80	Vitis vinifera	Glucan endo-1,3-beta-D-glucosidase (EC 3.2.1.39)	VIT_05s0077g01150	Q9M563_VITVI	10	39,70	39,48	78,57	1,04E+09
-872	6,59	-4,25	-5,12	-6,59	Vitis vinifera	Glucan endo-1,3-beta-D-glucosidase (EC 3.2.1.39)	VIT_08s0007g06040	F6HLL9_VITVI	12	63,50	36,68	323,31	2,76E+10
-872	6,09	-5,55	-4,95	-6,09	Vitis vinifera	zf-RVT domain-containing protein	VITISV_017450	A5C5J9_VITVI	4	31,50	16,44	10,82	7,07E+08
-872	5,93	-5,02	-5,49	-5,93	Vitis vinifera	Phytocyanin domain-containing protein	VIT_12s0034g01140	D7T037_VITVI	2	31,70	12,83	30,92	3,81E+09
-872	5,61	-5,20	-5,10	-5,61	Vitis vinifera	Peroxidase (EC 1.11.1.7)	VIT_06s0004g07770	D7SJL8_VITVI	12	64,20	34,06	187,46	6,25E+09
-872	6,62	-5,52	-5,26	-6,62	Vitis vinifera	Superoxide dismutase (EC 1.15.1.1)	VITISV_024204	A5BR41_VITVI	13	65,80	25,28	147,14	5,37E+09
-872	6,96	-4,59	-5,05	-6,96	Vitis vinifera	ATP synthase subunit d, mitochondrial	VIT_13s0019g03860	A5AY42_VITVI	11	61,30	19,77	90,45	3,03E+09
-872	2,50	-2,50	-2,23	-2,18	Vitis vinifera	Glutamine synthetase (EC 6.3.1.2)	VIT_17s0000g01910	F6GSQ2_VITVI	18	67,10	39,20	323,31	3,33E+09
-872	2,08	-1,54	-1,96	-2,08	Vitis vinifera	TauD domain-containing protein	VIT_09s0002g03450	F6HY97_VITVI	2	8,00	36,38	11,35	4,49E+07
-872	3,13	-3,13	-1,87	-2,94	Vitis vinifera	14_3_3 domain-containing protein	VIT_19s0014g01420	F6H2P0_VITVI	12	60,20	29,93	92,65	1,66E+09
-872	2,41	-2,41	0,00	-2,13	Vitis vinifera	Usp domain-containing protein	VIT_03s0038g04750	D7U4I8_VITVI	3	28,90	18,90	13,51	2,05E+08
-872	2,87	-2,87	-1,79	-1,72	Vitis vinifera	Allene-oxide cyclase (EC 5.3.99.6)	VITISV_032173	A5C260_VITVI	4	17,10	27,59	15,33	4,32E+08
-872	2,28	-2,28	-1,92	-1,55	Vitis vinifera	Peptidyl-prolyl cis-trans isomerase (PPIase) (EC 5.2.1.8	VITISV_015871	A5C2G6_VITVI	6	47,50	21,52	28,34	2,02E+09
-872	2,23	-1,48	-2,23	-1,97	Vitis vinifera	Uncharacterized protein	VITISV_037578	A5C5V6_VITVI	4	56,10	12,13	119,50	2,87E+09
-872	2,31	-1,99	-2,07	-2,31	Vitis vinifera	Uncharacterized protein	VITISV_019646	A5B835_VITVI	5	19,90	29,38	17,32	1,05E+09
-872	2,64	-2,06	-2,04	-2,64	Vitis vinifera	Uncharacterized protein	VIT_03s0038g02130	F6I111_VITVI	2	17,80	19,85	26,09	2,54E+08
-872	3,57	-1,94	-2,66	-3,57	Vitis vinifera	Uncharacterized protein	VIT_03s0180g00010	F6I5Z3_VITVI	6	16,20	47,38	83,21	7,42E+08
-872	2,88	-2,18	-2,88	-2,64	Vitis vinifera	Dihydrolipoyl dehydrogenase (EC 1.8.1.4)	VIT_05s0020g00380	F6HE11_VITVI	20	60,20	52,92	86,72	5,66E+09
-872	3,54	-3,54	-2,79	-3,32	Vitis vinifera	PKS_ER domain-containing protein	VIT_00s0218g00010	F6I719_VITVI	6	23,60	42,98	20,84	2,80E+08
-872	3,09	-1,79	-2,41	-3,09	Vitis vinifera	Uncharacterized protein	VIT_06s0004g01910	F6GU55_VITVI	13	44,30	42,84	23,49	7,59E+08
-872	3,56	-2,68	-3,50	-3,56	Vitis vinifera	Fumarate hydratase (EC 4.2.1.2)	VIT_07s0005g00880	D7U2D2_VITVI	7	26,70	53,53	43,10	7,90E+08
-872	3,55	-2,24	-3,55	-3,28	Vitis vinifera	Acetyltransferase component of pyruvate dehydrogenase complex (EC 2.3.1.12)	VIT_09s0002g01800	D7TZW9_VITVI	15	29,20	60,39	59,57	2,05E+09
-872	2,73	-1,65	-2,61	-2,73	Vitis vinifera	Cytochrome b5 heme-binding domain-containing protein	VIT_01s0011g00210	D7T9Y7_VITVI	2	29,90	14,79	1,20	9,27E+07
-872	3,44	-2,02	-3,01	-3,44	Vitis vinifera	Glycerophosphodiester phosphodiesterase (EC 3.1.4.46)	VIT_17s0000g07450	D7SHZ8_VITVI	8	28,40	45,37	18,99	2,92E+08
-872	3,37	-2,20	-3,21	-3,37	Vitis vinifera	Uncharacterized protein	VITISV_032127	A5ARJ3_VITVI	2	12,40	36,96	2,19	6,65E+07
-872	3,46	-3,00	-3,46	-3,24	Vitis vinifera	Glyco_hydro_19_cat domain-containing protein	VITISV_043424	A5C2S9_VITVI	2	15,40	27,93	69,46	3,16E+08
-872	2,67	-2,67	-2,66	-2,03	Vitis vinifera	Str_synth domain-containing protein	VIT_04s0210g00050	D7U6N0_VITVI	3	7,90	46,26	3,99	6,65E+07
-872	2,97	-2,57	-2,97	-2,27	Vitis vinifera	Alpha-galactosidase (EC 3.2.1.22) (Melibiase)	VITISV_000962	A5ALZ9_VITVI	6	18,10	44,37	18,91	1,36E+08
-872	3,65	-2,63	-3,65	-1,96	Vitis vinifera	Amidohydro-rel domain-containing protein	VIT_06s0009g02790	D7T187_VITVI	12	30,20	58,06	62,58	1,80E+09
-872	3,34	-3,34	-2,99	-2,33	Vitis vinifera	ATP synthase subunit alpha, mitochondrial	VIT_00s0733g00010	F6I2F8_VITVI	21	46,20	55,14	203,57	1,60E+10
-872	2,93	-2,93	-2,77	-2,52	Vitis vinifera	Uncharacterized protein	VIT_04s0023g00820	D7SPM6_VITVI	4	21,10	35,05	21,94	2,51E+08
-872	3,17	-2,76	-3,17	-2,08	Vitis vinifera	Glutathione peroxidase	VIT_05s0102g00120	A5AU08_VITVI	3	18,00	18,63	2,99	4,62E+07
-872	3,32	-2,99	-3,32	-2,30	Vitis vinifera	Nucleoside diphosphate kinase (EC 2.7.4.6)	VITISV_036798	A5B878_VITVI	8	64,90	16,31	173,69	1,16E+10
-872	3,85	-3,42	-3,61	-3,85	Vitis vinifera	CYTOSOL_AP domain-containing protein	VITISV_029979	A5C524_VITVI	16	43,10	60,74	86,66	1,34E+09
-872	4,19	-2,53	-3,17	-4,19	Vitis vinifera	(E)-beta-caryophyllene synthase (Beta-caryophyllene synthase)	VvivMATPS27	E5GAF4_VITVI	2	4,20	60,54	2,82	2,66E+07
-872	3,75	-2,24	-3,50	-3,75	Vitis vinifera	Isocitrate dehydrogenase [NADP] (EC 1.1.1.42)	VIT_04s0079g00530	A5BX54_VITVI	16	39,80	46,28	125,69	1,99E+09

Cluster n°	Isabel (log2 fold-change)	Tenta (log2 fold-change)	Sciascinoso (log2 fold-change)	Aglianico (log2 fold-change)	Organism	Protein name	Gene name	UniProt name	Peptides	Sequence coverage [%]	Mol. weight [kDa]	Score	Intensity
-872	4,37	-2,87	-3,59	-4,37	Vitis riparia (Frost grape) (Vitis vulpina)	ATP synthase (Fragment)	ATP synthase	F8U368_VITRI	11	40,80	27,68	50,38	2,30E+09
-872	4,26	-2,31	-3,56	-4,26	Vitis vinifera	Carbonic anhydrase (EC 4.2.1.1) (Carbonate dehydratase)	VIT_14s0066g01210	D7TWP2_VITVI	5	18,10	36,39	19,42	4,01E+08
-872	5,27	-4,09	-5,27	-3,37	Vitis vinifera	Succinate--CoA ligase [ADP-forming] subunit alpha, mitochondrial (EC 6.2.1.5) (Succinyl-CoA synthetase subunit alpha) (SCS-alpha)	VIT_17s0053g00120	D7TXP9_VITVI	6	31,00	34,88	34,88	8,25E+08
-872	4,63	-3,07	-4,63	-3,27	Vitis vinifera	PHB domain-containing protein	VIT_01s0011g00540	D7T9W2_VITVI	8	20,80	43,55	13,90	6,46E+08
-872	5,22	-3,40	-5,22	-3,21	Vitis vinifera	Superoxide dismutase [Cu-Zn] (EC 1.15.1.1)	VIT_06s0061g00750	D7SNA2_VITVI	5	41,50	21,71	162,75	2,47E+09
-872	4,77	-2,99	-4,77	-3,50	Vitis vinifera	Succinate--CoA ligase [ADP-forming] subunit beta, mitochondrial (EC 6.2.1.5) (Succinyl-CoA synthetase beta chain) (SCS-beta)	VIT_07s0005g03790	A5BF93_VITVI	14	41,80	45,40	172,91	3,09E+09
-872	3,91	-3,89	-3,91	-2,58	Vitis vinifera	Transket_pyr domain-containing protein	VIT_13s0019g00510	F6HN88_VITVI	31	47,00	106,54	323,31	5,24E+09
-872	4,09	-3,11	-4,09	-2,32	Vitis vinifera	C2 domain-containing protein	VIT_16s0050g00870	F6H6F0_VITVI	4	9,60	66,21	6,84	2,98E+08
-872	3,72	-3,49	-3,72	-2,33	Vitis vinifera	Citrate synthase	VITISV_022331	A5AGI7_VITVI	14	48,30	52,39	156,40	2,35E+09
-872	3,91	-3,37	-3,91	-3,19	Vitis riparia (Frost grape) (Vitis vulpina);Vitis vinifera	Stress-related protein	SRP	SRP_VITRI	4	23,00	27,61	27,12	1,70E+08
-872	4,42	-3,40	-4,42	-2,36	Vitis vinifera	Methylmalonate-semialdehyde dehydrogenase (CoA acylating) (EC 1.2.1.27)	VIT_00s0218g00070	F6I723_VITVI	9	28,80	58,06	78,71	7,48E+08
-873	7,43	6,77	6,90	-7,43	Vitis vinifera	Abhydrolase_3 domain-containing protein	VIT_03s0063g00810	F6HQD6_VITVI	9	35,10	34,86	78,60	1,18E+09
-873	6,20	6,60	5,84	-6,60	Vitis vinifera	Monodehydroascorbate reductase	*N/A*	A5JPK7_VITVI	13	45,20	47,28	12,88	3,95E+08
-873	6,53	5,20	6,03	-6,53	Vitis vinifera	DUF953 domain-containing protein	VIT_13s0064g01450	D7T2W1_VITVI	5	40,90	15,09	30,95	3,01E+08
-873	3,85	3,63	2,76	-3,85	Vitis vinifera	Uncharacterized protein	VIT_16s0050g01490	E0CUS6_VITVI	4	34,30	19,31	22,34	4,74E+08
-873	3,70	3,18	2,72	-3,70	Vitis vinifera	Aldo_ket_red domain-containing protein	VIT_05s0062g01000	D7U9L9_VITVI	13	44,60	35,01	7,10	2,28E+08
-873	3,74	3,57	2,40	-3,74	Vitis vinifera	Dehydroascorbate reductase	DHAR	A9UFY0_VITVI	6	42,00	23,69	14,44	6,34E+08
-873	4,62	3,81	2,78	-4,62	Vitis vinifera	CASP-like protein	VIT_02s0025g04210	D7TW56_VITVI	3	9,10	39,03	6,95	1,32E+08
-873	4,62	3,81	2,78	-4,62	Vitis vinifera	UDP-glucose 4-epimerase (EC 5.1.3.-)	VITISV_009281	A5AK58_VITVI	3	9,10	39,03	6,95	1,32E+08
-873	2,74	4,49	3,30	-4,49	Vitis vinifera	Methyltransferase (EC 2.1.1.-)	VIT_01s0026g02340	F6HPC4_VITVI	6	15,70	68,72	9,42	2,32E+08
-873	3,55	3,69	3,46	-3,69	Vitis vinifera	Uncharacterized proteinacy	VITISV_010455	A5BUW9_VITVI	9	44,40	23,68	131,11	4,64E+09
-873	3,44	3,69	4,17	-4,17	Vitis vinifera	Uncharacterized protein	VITISV_014635	A5BPV5_VITVI	5	23,30	33,43	8,75	2,62E+08
-873	3,41	3,01	3,95	-3,95	Vitis vinifera	L-ascorbate peroxidase (EC 1.11.1.11)	VIT_03s0038g02320	F6I106_VITVI	7	21,30	44,23	17,64	5,59E+08
-873	3,07	2,23	3,63	-3,63	Vitis vinifera	DUF3700 domain-containing protein	VIT_19s0014g03130	E0CSN8_VITVI	6	29,80	27,94	65,67	7,87E+08
-873	4,38	2,42	3,81	-4,38	Vitis vinifera	HMA domain-containing protein	VIT_13s0074g00770	D7UBY5_VITVI	4	58,10	9,00	18,29	1,04E+09
-873	4,20	2,65	3,66	-4,20	Vitis vinifera	Proteasome subunit beta (EC 3.4.25.1)	VIT_07s0151g00690	A5BYC0_VITVI	6	32,70	22,49	13,96	9,30E+08
-873	4,06	2,44	4,29	-4,29	Vitis vinifera	NTF2 domain-containing protein	VIT_01s0010g03790	A5BM29_VITVI	2	23,60	13,59	6,40	1,45E+08
-873	2,84	2,55	3,27	-3,27	Vitis vinifera	Uncharacterized protein	VIT_19s0090g00390	D7T8C3_VITVI	3	36,70	13,75	25,26	1,94E+08
-873	2,87	2,58	4,23	-4,23	Vitis vinifera	Cyanate hydratase (Cyanase) (EC 4.2.1.104) (Cyanate hydrolase) (Cyanate lyase)	CYN VITISV_023919	CYNS_VITVI	4	40,20	18,52	48,04	7,27E+08
-873	4,55	4,54	3,59	-4,55	Vitis vinifera	GST N-terminal domain-containing protein	VIT_05s0051g00320	F6HS41_VITVI	7	33,90	26,53	11,67	4,21E+08
-873	6,29	5,04	4,86	-6,29	Vitis vinifera	Small ubiquitin-related modifier (SUMO)	VIT_00s0186g00080	D7UDJ9_VITVI	3	26,70	11,29	24,57	2,23E+08
-873	5,38	3,39	3,77	-5,38	Vitis vinifera	Peptide-methionine (R)-S-oxide reductase (EC 1.8.4.12)	VIT_00s0282g00010	F6I1B3_VITVI	3	29,00	14,92	7,23	4,06E+08
-873	4,87	2,96	4,01	-4,87	Vitis vinifera	Nucleoside diphosphate kinase (EC 2.7.4.6)	VIT_02s0025g01500	A5BVN4_VITVI	2	8,90	25,56	4,40	1,81E+08
-873	4,93	4,62	5,02	-5,02	Vitis pseudoreticulata (Chinese wild grapevine)	Pathogenesis-related protein 17	*N/A*	H6WJJ6_9ROSI	7	40,70	25,37	74,72	1,20E+09
-873	6,06	5,56	3,97	-6,06	Vitis hybrid cultivar	Expansin	Tam-exps	E3WHD4_9ROSI	3	15,90	26,72	7,34	1,37E+09
-873	4,84	4,30	3,61	-4,84	Vitis vinifera	HTH cro/C1-type domain-containing protein	VIT_12s0028g02020	A5BHJ0_VITVI	2	16,20	15,77	7,43	1,41E+08
-873	4,71	5,22	3,95	-5,22	Vitis vinifera	Uncharacterized protein	VIT_19s0014g01520	F6H2P8_VITVI	7	20,60	45,18	25,77	7,43E+08
-873	5,25	3,74	3,32	-5,25	Vitis vinifera	14_3_3 domain-containing protein	VIT_07s0191g00090	A5AEH1_VITVI	14	60,40	28,78	206,92	3,25E+09
-873	6,03	4,96	4,96	-6,03	Vitis vinifera	Uncharacterized protein	VIT_17s0000g02480	D7SJA1_VITVI	3	46,20	10,36	32,92	2,33E+08
-845	2,27	3,24	2,42	-3,24	Vitis vinifera	Pectinesterase (EC 3.1.1.11)	VIT_04s0044g01000	F6I0G4_VITVI	6	13,20	60,69	19,53	3,28E+08
-845	3,17	1,89	2,01	-3,17	Vitis vinifera	Uncharacterized protein	VIT_02s0012g01250	F6HT95_VITVI	8	32,70	35,13	32,21	4,41E+08
-845	3,22	1,71	2,04	-3,22	Vitis vinifera	GST C-terminal domain-containing protein	VIT_01s0011g01890	D7T9J1_VITVI	6	17,00	45,29	60,73	7,40E+08

Cluster n°	Isabel (log2 fold-change)	Tenta (log2 fold-change)	Sciascinoso (log2 fold-change)	Aglianico (log2 fold-change)	Organism	Protein name	Gene name	UniProt name	Peptides	Sequence coverage [%]	Mol. weight [kDa]	Score	Intensity
-845	1,44	2,48	1,46	-2,48	Vitis vinifera	Tripeptidyl-peptidase II (EC 3.4.14.10)	VIT_16s0050g00490	F6H6M8_VITVI	8	6,80	151,10	12,75	3,15E+08
-845	1,92	2,93	1,75	-2,93	Vitis vinifera	Uncharacterized protein	VIT_06s0004g00470	F6GUN2_VITVI	9	33,30	36,93	163,02	4,14E+09
-845	2,15	3,21	1,78	-3,21	Vitis vinifera	Probable bifunctional methylthioribulose-1-phosphate dehydratase/enolase-phosphatase E1 1 [Includes: Methylthioribulose-1-phosphate dehydratase (MTRu-1-P dehydratase) (EC 4.2.1.109)	VIT_19s0014g02480	MTBC1_VITVI	3	10,30	57,09	38,75	2,62E+08
-845	2,07	3,11	1,40	-3,11	Vitis vinifera	Ferredoxin--NADP reductase, chloroplastic (FNR) (EC 1.18.1.2)	VIT_10s0003g04880	D7TKJ3_VITVI	2	10,10	42,34	6,80	1,19E+08
-845	1,47	2,02	2,40	-2,40	Vitis vinifera	UBC core domain-containing protein	VIT_08s0007g02580	D7TH48_VITVI	9	76,70	16,59	37,69	1,22E+09
-845	2,49	2,30	2,49	-2,49	Vitis vinifera	Uncharacterized protein	VIT_00s0153g00050	F6HJH8_VITVI	3	21,30	25,93	17,23	3,06E+08
-845	1,91	3,77	2,23	-3,77	Vitis vinifera	Uncharacterized protein	VIT_15s0046g03180	D7UC61_VITVI	4	19,40	21,54	5,63	8,36E+08
-845	2,51	1,69	0,00	-2,51	Vitis vinifera	Aspartate--tRNA ligase (EC 6.1.1.12)	VIT_04s0008g03010	F6H2W4_VITVI	12	26,50	60,86	49,48	9,03E+08
-845	2,62	2,36	0,00	-2,62	Vitis vinifera	S10_plectin domain-containing protein	VIT_16s0050g01160	F6H6C2_VITVI	7	43,90	19,96	45,67	7,02E+08
-845	1,34	2,04	0,00	-2,04	Vitis vinifera	3-methyl-2-oxobutanoate hydroxymethyltransferase (EC 2.1.2.11)	VIT_15s0046g02170	F6I650_VITVI	3	15,70	38,56	18,27	1,58E+08
-845	1,96	1,78	0,00	-1,96	Vitis vinifera	Inorganic diphosphatase (EC 3.6.1.1)	VIT_04s0023g02050	D7SPB2_VITVI	12	67,90	24,35	108,21	3,08E+09
-845	1,48	1,84	0,00	-1,84	Vitis vinifera	AB hydrolase-1 domain-containing protein	VIT_07s0005g00700	F6HZ61_VITVI	2	9,10	36,20	10,63	1,13E+08
-844	1,36	0,00	0,00	-1,36	Vitis vinifera	Protein SCAR (Protein WAVE)	VITISV_042694	A5B0E9_VITVI	19	39,80	72,77	148,09	1,57E+09
-844	1,90	0,00	0,00	-1,90	Vitis vinifera	Glutathione peroxidase	VIT_02s0025g03600	F6HUD1_VITVI	7	47,90	18,77	97,64	6,74E+09
-844	1,48	0,00	0,00	-1,48	Vitis vinifera	Superoxide dismutase (EC 1.15.1.1);Superoxide dismutase [Cu-Zn] (EC 1.15.1.1)	VIT_14s0030g00830	F6HTY5_VITVI	4	34,40	15,34	135,01	5,98E+09
-844	1,72	0,00	1,34	-1,72	Vitis vinifera	Abhydrolase_3 domain-containing protein	VIT_03s0063g00830	F6HQD8_VITVI	4	18,20	33,15	23,70	3,89E+08
-844	2,20	-1,47	0,00	-2,20	Vitis vinifera	Epimerase domain-containing protein	VIT_07s0005g04440	D7U382_VITVI	7	22,80	43,93	17,58	4,66E+08
-844	1,30	0,00	0,00	-1,30	Vitis vinifera	Diphosphomevalonate decarboxylase (EC 4.1.1.33)	VIT_13s0106g00790	D7TXN8_VITVI	9	28,70	46,64	27,51	1,11E+09
-844	1,77	0,00	0,00	-1,77	Vitis vinifera	Proline iminopeptidase (EC 3.4.11.5)	VIT_03s0088g01170	D7T3J3_VITVI	8	26,90	43,92	45,54	3,63E+08
-844	1,35	0,00	0,00	-1,35	Vitis vinifera	Uncharacterized protein	VIT_04s0008g05650	D7SUW1_VITVI	3	17,30	25,04	11,20	2,63E+08
-844	1,47	0,00	0,00	-1,47	Vitis vinifera	Uncharacterized protein	VIT_11s0016g05770	F6HHB0_VITVI	6	8,90	81,19	4,51	6,24E+07
-844	1,60	0,00	0,00	-1,60	Vitis vinifera	Uncharacterized protein	VIT_18s0001g12730	A5C3G7_VITVI	9	55,50	29,57	80,89	1,76E+09
-844	1,50	-1,50	0,00	-1,50	Vitis vinifera	Uncharacterized protein	VIT_02s0012g00760	D7TU16_VITVI	8	38,90	26,98	41,94	3,67E+08
-844	1,63	0,00	0,00	-1,63	Vitis vinifera	PKS_ER domain-containing protein	VIT_00s0301g00170	D7SSH1_VITVI	5	16,60	34,42	11,73	2,75E+08
-844	2,03	0,00	0,00	-2,03	Vitis quinquangularis	Calmodulin	CaM	B3GG02_9ROSI	8	75,80	16,85	323,31	3,66E+09
-844	1,74	0,00	-1,74	0,00	Vitis vinifera	GOLD domain-containing protein	VIT_19s0015g00700	D7UAI9_VITVI	4	32,70	24,69	34,68	4,28E+08
-844	1,77	0,00	-1,77	0,00	Vitis vinifera	PHB domain-containing protein	VIT_09s0002g01630	D7TZV6_VITVI	3	8,10	45,67	4,98	1,21E+08
-844	2,24	0,00	-2,24	0,00	Vitis vinifera	AAA domain-containing protein	VIT_09s0002g00170	D7TZI9_VITVI	12	31,90	47,68	39,71	5,53E+08
-844	1,42	0,00	-1,42	0,00	Vitis vinifera	V-type proton ATPase subunit C	VIT_14s0068g01280	D7SVJ2_VITVI	10	29,90	42,67	36,95	8,43E+08
-844	2,52	0,00	-1,63	-2,52	Vitis vinifera	Proteasome subunit beta (EC 3.4.25.1)	VIT_04s0008g02850	D7SU52_VITVI	3	15,20	24,04	25,46	1,56E+08
-844	1,48	0,00	-1,48	0,00	Vitis vinifera	Uncharacterized protein	VIT_06s0004g04930	D7SKD8_VITVI	6	19,10	52,02	16,47	4,53E+08
-844	2,26	0,00	-2,26	-1,99	Vitis vinifera	Cysteine protease	CysP	A9UFX8_VITVI	2	9,30	41,49	7,59	1,30E+08
-844	1,98	0,00	-1,73	-1,98	Vitis vinifera	14_3_3 domain-containing protein	VIT_14s0006g03230	A5C3B4_VITVI	16	72,50	29,54	285,13	4,48E+09
-844	1,54	0,00	-1,54	0,00	Vitis vinifera	Uncharacterized protein	VIT_09s0002g02600	A5BP92_VITVI	2	10,40	22,47	4,90	1,45E+08
-844	1,82	0,00	-1,82	0,00	Vitis vinifera	DUF3700 domain-containing protein	VIT_05s0020g04160	D7T745_VITVI	13	65,50	27,17	82,92	3,14E+09
-844	1,41	0,00	-1,41	0,00	Vitis vinifera	Proteasome subunit alpha type	VIT_08s0040g00410	A5AXI5_VITVI	10	43,00	27,19	78,08	1,87E+09
-844	1,76	0,00	-1,76	0,00	Vitis vinifera	Uncharacterized protein	VIT_11s0052g01030	D7SQ96_VITVI	5	32,10	29,72	12,23	8,02E+08
-844	1,40	0,00	-1,40	0,00	Vitis vinifera	MBD domain-containing protein	VIT_11s0016g05580	F6HHA3_VITVI	2	13,20	34,39	1,39	1,16E+08
-844	2,31	0,00	-2,31	-1,48	Vitis vinifera	Uncharacterized protein	VITISV_003163	A5AG99_VITVI	3	47,60	8,77	21,54	8,48E+07
-844	1,93	-1,93	-1,75	0,00	Vitis vinifera	Pyruvate dehydrogenase E1 component subunit beta (EC 1.2.4.1)	VIT_01s0146g00310	F6I1P0_VITVI	10	43,10	39,49	160,57	2,83E+09
-844	1,65	-1,65	0,00	0,00	Vitis vinifera	PCI domain-containing protein	VIT_02s0012g02660	F6HT17_VITVI	9	34,50	44,33	22,12	5,97E+08
-844	1,85	-1,85	-1,38	0,00	Vitis vinifera	Uncharacterized protein	VITISV_008170	A5BG56_VITVI	15	37,70	52,49	80,73	2,55E+09
-844	2,08	-1,70	-2,08	0,00	Vitis vinifera	Cysteine desulfurase (EC 2.8.1.7)	VITISV_036314	A5B9Q3_VITVI	13	44,50	49,86	52,40	1,11E+09
-844	1,42	-1,33	-1,42	0,00	Vitis vinifera	Uncharacterized protein	VIT_06s0004g04470	F6GV26_VITVI	20	39,30	71,34	23,72	1,25E+09
-844	1,67	-1,67	-1,38	0,00	Vitis vinifera	Fatty acid hydroperoxide lyase 1	VIT_12s0059g01060	F6HID6_VITVI	23	61,80	54,68	323,31	1,86E+09
-844	2,07	-2,07	0,00	0,00	Vitis vinifera	Malic enzyme	VIT_15s0046g03670	D7UC26_VITVI	21	45,30	66,70	99,05	1,92E+09
-844	2,07	-2,07	0,00	0,00	Vitis vinifera	Protein kinase domain-containing protein	VITISV_022074	A5BU69_VITVI	21	45,30	66,70	99,05	1,92E+09
-844	1,69	-1,69	0,00	0,00	Vitis vinifera	PHB domain-containing protein	VIT_01s0026g01640	D7TNE5_VITVI	7	25,50	41,34	11,36	5,38E+08

Cluster n°	Isabel (log2 fold-change)	Tenta (log2 fold-change)	Sciascinoso (log2 fold-change)	Aglianico (log2 fold-change)	Organism	Protein name	Gene name	UniProt name	Peptides	Sequence coverage [%]	Mol. weight [kDa]	Score	Intensity
-844	2,58	-2,58	-1,99	0,00	Vitis vinifera	Uncharacterized protein	VIT_01s0011g05610	D7T8M1_VITVI	5	23,40	22,86	13,49	4,08E+08
-844	1,95	-1,95	0,00	0,00	Vitis vinifera	Uncharacterized protein	VIT_17s0000g01240	F6GSW3_VITVI	5	41,20	26,83	12,22	4,10E+08
-844	2,10	-2,10	0,00	0,00	Vitis vinifera	Calreticulin	VIT_07s0005g01390	D7U2H8_VITVI	5	22,60	49,36	30,25	6,50E+08
-844	1,51	-1,51	-1,44	0,00	Vitis vinifera	Peptidase A1 domain-containing protein	VIT_13s0084g00820	F6HVD5_VITVI	4	12,40	39,51	12,71	2,52E+08
-844	1,85	-1,85	0,00	0,00	Vitis vinifera	Inositol-1-monophosphatase (EC 3.1.3.25)	VIT_10s0405g00030	E0CNP0_VITVI	3	12,80	28,42	12,61	1,86E+08
-844	2,10	-1,80	-2,10	0,00	Vitis quinquangularis	Aquaporin TIP1-3	*N/A*	A0A1Y0BWD7_9ROSI	2	10,40	26,14	8,53	1,15E+09
-844	1,80	-1,80	0,00	0,00	Vitis hybrid cultivar	Putative plastid lipid-associated protein (Fragment)	tamPAP-FB	A9CSJ8_9ROSI	7	50,40	25,89	82,06	1,35E+09
-825	0,00	1,65	1,79	-1,79	Vitis vinifera	Uncharacterized protein	VIT_08s0007g07530	F6HLA7_VITVI	8	50,00	26,90	62,59	4,62E+08
-825	0,00	1,49	1,43	-1,49	Vitis vinifera	UMP-CMP kinase (EC 2.7.4.14) (Deoxycytidylate kinase) (CK) (dCMP kinase) (Uridine monophosphate/cytidine monophosphate kinase) (UMP/CMP kinase) (UMP/CMPK)	VIT_15s0045g01310	D7U5T7_VITVI	7	38,10	23,53	11,61	3,27E+08
-825	0,00	1,37	1,86	-1,86	Vitis vinifera	NADH dehydrogenase [ubiquinone] 1 alpha subcomplex subunit 12	VIT_16s0039g02180	D7T7R0_VITVI	5	44,90	18,36	7,90	3,47E+08
-825	0,00	1,67	1,41	-1,67	Vitis vinifera	Transaldolase (EC 2.2.1.2)	VIT_00s0233g00020	D7T5J5_VITVI	3	8,80	43,38	4,89	1,40E+08
-825	0,00	1,35	1,53	-1,53	Vitis vinifera	6,7-dimethyl-8-ribityllumazine synthase (DMRL synthase) (EC 2.5.1.78)	VIT_05s0094g01490	D7T2N4_VITVI	4	22,10	19,19	6,43	1,99E+08
-825	0,00	1,63	1,86	-1,86	Vitis vinifera	HABP4_PAI-RBP1 domain-containing protein	VIT_15s0021g01420	D7SLZ5_VITVI	8	33,00	38,72	30,77	7,90E+08
-825	0,00	1,54	2,00	-2,00	Vitis vinifera	Uncharacterized protein	VITISV_033286	A5BUC9_VITVI	32	28,00	160,48	116,54	5,33E+08
-825	0,00	2,28	1,70	-2,28	Vitis vinifera	Uncharacterized protein	VIT_06s0004g02840	F6GU07_VITVI	5	41,00	17,79	38,99	1,82E+09
-825	0,00	0,00	2,19	-2,19	Vitis vinifera	Isopentenyl-diphosphate Delta-isomerase (EC 5.3.3.2)	*N/A*	G4XGW7_VITVI	11	44,50	33,17	7,87	1,81E+08
-825	0,00	0,00	1,92	-1,92	Vitis vinifera	Uncharacterized protein	VIT_03s0017g01690	F6HTM3_VITVI	27	31,40	125,93	48,96	2,14E+08
-825	0,00	0,00	1,51	-1,51	Vitis vinifera	Stress-response A/B barrel domain-containing protein	VIT_05s0077g00170	D7SYF3_VITVI	2	23,90	12,40	8,71	2,02E+08
-825	0,00	0,00	1,88	-1,88	Vitis vinifera	Methylenetetrahydrofolate reductase (EC 1.5.1.20)	VITISV_016119	A5ATB7_VITVI	10	22,90	66,90	40,03	6,23E+08
-825	1,33	0,00	1,84	-1,84	Vitis vinifera	NADH dehydrogenase [ubiquinone] flavoprotein 1, mitochondrial (EC 7.1.1.2)	VIT_19s0027g00780	F6H4T6_VITVI	5	12,40	52,13	19,31	4,49E+08
-863	-3,73	-3,45	-3,63	3,73	Vitis vinifera	Glyco_hydro_18 domain-containing protein	VIT_15s0046g01600	F6I685_VITVI	4	20,50	34,03	59,60	2,22E+08
-863	-3,67	-3,85	-2,75	3,85	Vitis vinifera	Uncharacterized protein	VIT_02s0025g04340	F6HUH2_VITVI	7	49,60	28,83	79,31	2,41E+08
-863	-4,65	-4,35	-4,62	4,65	Vitis vinifera	Uncharacterized protein	VIT_08s0007g08310	F6HL03_VITVI	25	42,00	87,23	323,31	1,56E+08
-863	-5,64	-4,43	-5,27	5,64	Vitis vinifera	Bet_v_1 domain-containing protein	VIT_01s0011g05180	F6HFH0_VITVI	6	57,00	17,19	30,03	6,64E+08
-863	-3,78	-3,71	-4,12	4,12	Vitis vinifera	NTF2 domain-containing protein	VIT_10s0116g00570	F6H7L1_VITVI	3	33,60	15,92	12,78	2,37E+08
-863	-5,04	-3,49	-3,08	5,04	Vitis vinifera	Uncharacterized protein	VIT_06s0061g00410	F6GWA0_VITVI	12	24,90	66,84	125,67	4,35E+08
-863	-4,45	-4,20	-3,17	4,45	Vitis vinifera	Uncharacterized protein	VIT_18s0001g14630	E0CQ92_VITVI	4	10,90	61,39	4,58	1,50E+08
-863	-3,30	-4,00	-3,40	4,00	Vitis vinifera	Flavin-containing monooxygenase (EC 1.-.-.-)	VIT_03s0038g03130	D7U4Y4_VITVI	6	16,20	58,83	24,51	1,94E+08
-863	-3,24	-3,36	-3,61	3,61	Vitis vinifera	Dolichyl-diphosphooligosaccharide--protein glycosyltransferase subunit 1	VIT_13s0019g04410	D7TM83_VITVI	7	27,30	52,59	13,87	2,97E+08
-863	-3,91	-3,10	-3,08	3,91	Vitis vinifera	14_3_3 domain-containing protein	VIT_10s0003g01240	D7TJL0_VITVI	10	47,60	32,38	33,27	5,55E+08
-863	-4,94	-5,04	-4,31	5,04	Vitis vinifera	AMP-binding domain-containing protein	VIT_12s0057g01270	D7TD95_VITVI	17	38,90	78,85	130,91	1,89E+09
-863	-2,62	-4,27	-3,69	4,27	Vitis vinifera	GTP-binding nuclear protein	VIT_09s0002g05940	F6HX89_VITVI	8	38,90	25,10	94,91	7,58E+09
-863	-4,08	-3,93	-2,36	4,08	Vitis vinifera	Chlorophyll a-b binding protein, chloroplastic	VIT_08s0007g02190	F6HKS7_VITVI	8	37,50	31,43	97,39	4,59E+09
-863	-4,05	-3,30	-2,78	4,05	Vitis vinifera	MPN domain-containing protein	VIT_07s0104g01080	F6HQ30_VITVI	8	34,50	46,06	33,86	7,50E+08
-863	-4,66	-3,26	-3,50	4,66	Vitis vinifera	DLH domain-containing protein	VIT_05s0020g02550	F6HDK7_VITVI	9	49,00	26,83	5,72	1,98E+08
-863	-4,84	-3,47	-4,02	4,84	Vitis vinifera	Chalcone-flavonone isomerase family protein	VIT_13s0067g03820	D7T475_VITVI	9	56,40	25,08	7,92	1,61E+08
-863	-3,56	-3,15	-3,75	3,75	Vitis vinifera	Plasma membrane ATPase (EC 7.1.2.1)	VITISV_027896	A5ALY9_VITVI	31	40,40	103,37	30,78	4,59E+08
-863	-4,44	-3,99	-5,06	5,06	Vitis vinifera	Uncharacterized protein	VITISV_010558	A5BL42_VITVI	5	25,70	22,01	102,09	1,67E+08
-863	-4,38	-3,58	-2,32	4,38	Vitis vinifera	Photosystem II CP43 reaction center protein (PSII 43 kDa protein) (Protein CP-43)	psbC	PSBC_VITVI	13	33,80	51,84	323,31	6,08E+09
-863	-2,98	-4,81	-5,01	5,01	Vitis vinifera	Alpha-galactosidase (EC 3.2.1.22) (Melibiase)	VITISV_043596	A5ACL5_VITVI	7	25,30	46,85	131,26	5,45E+08
-863	-2,73	-2,00	-2,82	2,82	Vitis vinifera	MFS domain-containing protein	VITISV_024656	A5BH92_VITVI	11	24,90	79,46	134,85	3,16E+09
-863	-2,02	-2,61	-2,07	2,61	Vitis vinifera	Phospholipase D (EC 3.1.4.4)	VIT_12s0035g00200	F6HK82_VITVI	7	8,80	99,15	4,28	8,80E+07
-863	-2,70	-2,21	-2,76	2,76	Vitis vinifera	Thiamine thiazole synthase 1, chloroplastic (EC 2.4.2.60) (Thiazole biosynthetic enzyme 1)	THI1-1	THI41_VITVI	2	7,40	37,51	5,12	1,37E+08
-863	-3,48	-2,31	-2,84	3,48	Vitis vinifera	Uncharacterized protein	VIT_18s0001g09990	F6GZY7_VITVI	7	22,10	51,40	223,94	2,59E+09

Cluster n°	Isabel (log2 fold-change)	Tenta (log2 fold-change)	Sciascinoso (log2 fold-change)	Aglianico (log2 fold-change)	Organism	Protein name	Gene name	UniProt name	Peptides	Sequence coverage [%]	Mol. weight [kDa]	Score	Intensity
-863	-2,44	-2,59	-2,69	2,69	Vitis vinifera	Homogentisate 1,2-dioxygenase (EC 1.13.11.5) (Homogentisate oxygenase) (Homogentisic acid oxidase) (Homogentisicase)	VIT_19s0014g01800	E0CSC9_VITVI	5	25,90	51,20	15,48	1,57E+08
-863	-2,56	-2,36	-2,44	2,56	Vitis vinifera	Class III chitinase (EC 3.2.1.14);	ChiFIII	B5M495_VITVI	2	10,40	30,84	48,03	2,28E+08
-863	-2,63	-3,22	-3,01	3,22	Vitis vinifera	Pyr_redox_2 domain-containing protein	VIT_08s0007g03610	D7TJ46_VITVI	14	48,20	47,32	156,62	1,93E+08
-863	-2,91	-2,12	-3,21	3,21	Vitis vinifera	V-ATPase 69 kDa subunit (EC 7.1.2.2) (V-type proton ATPase catalytic subunit A) (Vacuolar proton pump subunit alpha)	VIT_04s0008g02580	F6H3B6_VITVI	29	64,70	68,72	323,31	5,21E+09
-863	-2,94	-1,97	-1,90	2,94	Vitis vinifera	T-complex protein 1 subunit delta	VIT_17s0000g03040	F6GTS3_VITVI	12	26,10	57,59	33,03	8,16E+08
-863	-2,56	-2,60	-1,68	2,60	Vitis vinifera	Glyceraldehyde-3-phosphate dehydrogenase (EC 1.2.1.-)	VIT_19s0085g00600	D7T0U8_VITVI	6	23,70	39,23	24,80	1,64E+08
-863	-2,70	-3,18	-1,42	3,18	Vitis vinifera	Phospholipase D (EC 3.1.4.4)	PLD	Q09VU3_VITVI	45	73,70	91,80	323,31	3,03E+10
-863	-2,60	-2,10	-1,79	2,60	Vitis vinifera	NB-ARC domain-containing protein	VITISV_006174	A5AJ42_VITVI	15	41,90	56,12	282,65	5,22E+09
-863	-1,60	-2,25	-3,27	3,27	Vitis vinifera	Vacuolar proton pump subunit B (V-ATPase subunit B) (Vacuolar proton pump subunit B)	VIT_03s0038g02760	D7U516_VITVI	20	57,80	54,29	291,86	3,60E+09
-863	-2,06	-1,97	-3,74	3,74	Vitis vinifera	Protein-methionine-S-oxide reductase (EC 1.8.4.11)	VIT_16s0013g00620	D7U762_VITVI	7	42,20	23,12	56,33	7,18E+08
-863	-1,38	-1,65	-1,31	1,65	Vitis vinifera	EGF_CA domain-containing protein	VIT_01s0011g01050	F6HEY1_VITVI	3	4,30	70,28	2,09	1,25E+08
-863	-2,71	0,00	-1,87	2,71	Vitis vinifera	Glyco_hydro_3 domain-containing protein	VIT_13s0073g00550	F6HBH0_VITVI	5	15,70	68,60	7,00	3,02E+08
-863	0,00	-2,22	-2,65	2,65	Vitis vinifera	3-hydroxyacyl-CoA dehydrogenase (EC 1.1.1.35) (EC 4.2.1.17) (EC 5.1.2.3) (EC 5.3.3.8) (Enoyl-CoA hydratase/3-2-trans-enoyl-CoA isomerase/3-hydroxybutyryl-CoA epimerase)	VIT_05s0077g02140	F6H6Y0_VITVI	27	48,60	78,69	124,49	3,79E+09
-863	-2,30	0,00	-1,65	2,30	Vitis vinifera	Ubiquitin carboxyl-terminal hydrolase (EC 3.4.19.12)	VIT_19s0014g03920	F6H2F7_VITVI	4	8,60	90,09	6,41	1,35E+08
-863	-2,14	-1,48	-1,54	2,14	Vitis vinifera	AMP-binding domain-containing protein	VIT_02s0025g01410	D7TVF4_VITVI	13	23,30	74,27	50,86	7,94E+08
-863	-1,33	-1,96	-1,75	1,96	Vitis vinifera	Uncharacterized protein	VIT_08s0007g03620	D7TJ45_VITVI	4	58,80	11,50	12,86	1,66E+08
-863	-1,32	-1,51	-2,50	2,50	Vitis vinifera	Fe2OG dioxygenase domain-containing protein	VIT_18s0001g03430	E0CR99_VITVI	4	23,40	35,70	40,51	1,73E+08
-863	-1,77	-1,92	-1,75	1,92	Vitis vinifera	DLH domain-containing protein	VIT_12s0059g00890	F6HIC8_VITVI	9	43,20	26,28	72,92	4,77E+09
-863	-1,47	0,00	-1,66	1,66	Vitis vinifera	LEA_2 domain-containing protein	VIT_08s0007g02360	F6HKR7_VITVI	3	27,00	25,24	32,84	7,93E+07
-566	-1,65	0,00	1,65	1,41	Vitis vinifera	Catalase (EC 1.11.1.6)	VIT_04s0044g00020	F6I0K4_VITVI	23	67,90	56,97	323,31	1,53E+09
-566	-2,23	0,00	1,31	2,23	Vitis vinifera	Uncharacterized protein	VIT_07s0005g06490	F6I019_VITVI	6	31,60	23,52	9,65	1,88E+08
-566	-2,49	0,00	2,49	1,75	Vitis vinifera	Uncharacterized protein	VIT_00s0317g00130	F6HRR8_VITVI	2	17,90	14,27	25,17	3,03E+08
-566	-2,69	0,00	2,33	2,69	Vitis vinifera	Uncharacterized protein	VIT_01s0127g00590	F6HHU6_VITVI	5	14,80	65,19	34,93	8,72E+08
-566	-2,14	0,00	2,09	2,14	Vitis vinifera	UDP-glucose 6-dehydrogenase (EC 1.1.1.22)	VIT_01s0011g00690	F6HF82_VITVI	16	51,90	52,74	105,23	5,64E+08
-566	-2,14	0,00	1,31	2,14	Vitis vinifera	UDP-arabinopyranose mutase (EC 5.4.99.30)	VIT_14s0083g01100	F6GVV2_VITVI	16	67,60	40,80	73,99	1,08E+09
-566	-1,91	0,00	1,91	1,67	Vitis vinifera	Importin N-terminal domain-containing protein	VIT_16s0098g01630	E0CV68_VITVI	12	23,30	105,69	53,86	8,08E+08
-566	-1,39	0,00	1,39	1,39	Vitis vinifera	Peptidylprolyl isomerase (EC 5.2.1.8)	VIT_00s0260g00060	D7UDY0_VITVI	4	34,50	15,71	37,22	6,25E+08
-566	-2,07	0,00	1,85	2,07	Vitis vinifera	Uncharacterized protein	VIT_03s0038g00750	D7U5G4_VITVI	4	19,40	34,20	29,99	3,11E+08
-566	-2,64	0,00	2,64	2,09	Vitis vinifera	Aldedh domain-containing protein	VIT_14s0066g01550	D7TWS2_VITVI	19	46,70	58,06	150,15	1,90E+09
-566	-2,53	0,00	2,53	2,39	Vitis vinifera	Ubiquitin receptor RAD23 (DNA repair protein RAD23)	VIT_01s0011g03410	D7T959_VITVI	9	39,30	41,95	39,42	7,30E+08
-566	-2,37	0,00	2,37	2,16	Vitis vinifera	Alpha-1,4 glucan phosphorylase (EC 2.4.1.1)	VIT_01s0011g00730	F6HF78_VITVI	6	8,10	111,38	14,25	2,48E+08
-566	-2,89	0,00	2,69	2,89	Vitis vinifera	Proteasome subunit beta (EC 3.4.25.1)	VIT_06s0004g03190	D7SKV3_VITVI	5	39,60	27,57	100,47	1,14E+09
-566	-1,65	0,00	1,65	1,61	Vitis vinifera	L-ascorbate peroxidase (EC 1.11.1.11)	VIT_06s0004g03550	D7SKR5_VITVI	13	71,60	27,56	323,31	2,32E+09
-566	-2,02	0,00	2,00	2,02	Vitis vinifera	Pyr_redox_2 domain-containing protein	VIT_02s0025g00340	D7TV61_VITVI	10	27,80	53,36	40,23	8,29E+08
-566	-2,31	0,00	2,21	2,31	Vitis vinifera	CCT-beta	VIT_18s0001g00480	F6H0C8_VITVI	15	41,00	57,48	54,10	7,39E+08
-566	-2,46	0,00	2,46	1,34	Vitis vinifera	Clathrin light chain	VIT_13s0067g00550	F6HCE0_VITVI	8	36,70	34,92	46,16	6,51E+08
-566	-2,24	1,34	1,33	2,24	Vitis vinifera	PSI-F	VIT_00s0125g00280	F6H2R3_VITVI	4	29,40	24,22	28,19	7,57E+08
-566	-2,43	0,00	2,43	1,73	Vitis vinifera	ADH_zinc_N domain-containing protein	VITISV_019588	A5BYT8_VITVI	3	18,80	38,30	11,38	1,32E+08
-566	-2,21	0,00	2,21	1,47	Vitis vinifera	Fe2OG dioxygenase domain-containing protein	VIT_18s0001g14310	A5ANR7_VITVI	12	55,90	40,42	20,89	3,88E+08
-458	-2,81	1,42	2,81	0,00	Vitis vinifera	Chalcone synthase (EC 2.3.1.74)	VIT_05s0136g00260	Q8W3P6_VITVI	9	33,90	42,60	87,99	6,05E+08
-458	-2,62	1,50	2,62	0,00	Vitis vinifera	Uncharacterized protein	VIT_05s0124g00020	F6I3Q7_VITVI	7	24,40	54,63	28,19	3,33E+08
-458	-2,53	2,53	1,52	1,32	Vitis vinifera	Uncharacterized protein	VIT_11s0037g00510	F6HYK6_VITVI	8	18,30	71,40	15,42	8,63E+08
-458	-2,54	2,54	1,51	0,00	Vitis vinifera	RmlD_sub_bind domain-containing protein	VIT_02s0025g04610	F6HUI7_VITVI	4	18,20	33,67	8,71	2,08E+08
-458	-1,92	1,76	1,92	1,57	Vitis vinifera	Phosphoserine aminotransferase (EC 2.6.1.52)	VIT_00s2579g00010	F6H8F3_VITVI	5	16,00	38,42	19,24	3,72E+08
-458	-2,47	1,67	2,47	0,00	Vitis vinifera	Uncharacterized protein	VIT_18s0001g07230	F6H112_VITVI	2	20,60	20,59	3,76	9,64E+07
-458	-1,55	1,55	1,34	0,00	Vitis vinifera	Uncharacterized protein	VIT_12s0034g01870	D7SZX9_VITVI	13	51,70	38,53	323,31	1,04E+10
-458	-1,42	1,39	1,42	0,00	Vitis vinifera	HP domain-containing protein	VIT_11s0052g00670	D7SQC6_VITVI	8	11,90	108,00	27,78	4,48E+08
-458	-1,93	1,93	1,81	1,68	Vitis vinifera	Malate dehydrogenase (EC 1.1.1.37)	VITISV_032320	A5C6E2_VITVI	14	58,10	35,51	273,09	1,02E+10

Cluster n°	Isabel (log2 fold-change)	Tenta (log2 fold-change)	Sciascinoso (log2 fold-change)	Aglianico (log2 fold-change)	Organism	Protein name	Gene name	UniProt name	Peptides	Sequence coverage [%]	Mol. weight [kDa]	Score	Intensity
-458	-2,40	2,40	2,04	0,00	Vitis vinifera	GDP-mannose 4,6-dehydratase (EC 4.2.1.47)	VIT_04s0023g03170	F6GWP0_VITVI	7	22,50	41,87	22,99	3,79E+08
-458	-1,54	1,54	1,53	1,39	Vitis vinifera	40S ribosomal protein S27	VIT_01s0150g00310	D7TFK6_VITVI	2	34,90	9,61	5,22	3,07E+08
-458	-1,96	1,72	1,39	1,96	Vitis vinifera	Glycine cleavage system H protein	VITISV_038357	A5BA00_VITVI	4	48,50	17,55	26,47	9,66E+08
-879	-6,09	6,09	5,71	5,47	Vitis vinifera	Ribosomal_L23eN domain-containing protein	VIT_06s0004g04090	D7SKL7_VITVI	5	29,00	17,58	23,49	1,05E+09
-879	-5,63	5,63	5,55	4,93	Vitis vinifera	Ribosomal_L6e_N domain-containing protein	VIT_13s0064g01390	F6HB97_VITVI	10	41,60	26,05	34,07	1,06E+09
-879	-5,80	5,40	5,80	5,35	Vitis vinifera	Transketolase (EC 2.2.1.1)	VIT_15s0048g00370	F6I397_VITVI	23	48,80	78,86	323,31	1,03E+10
-879	-6,05	4,92	6,05	5,75	Vitis vinifera	DLH domain-containing protein	VITISV_041961	A5AMM1_VITVI	9	55,90	25,50	86,87	1,87E+09
-879	-5,52	5,48	5,52	5,26	Vitis vinifera	60S ribosomal protein L13	VIT_13s0047g01160	D7TF52_VITVI	8	42,00	23,83	86,84	9,49E+08
-879	-5,65	4,88	5,50	5,65	Vitis vinifera	Proteasome subunit beta (EC 3.4.25.1)	VIT_01s0137g00420	D7SME1_VITVI	2	8,40	29,50	4,56	3,59E+08
-879	-6,59	5,47	6,13	6,59	Vitis vinifera	Alpha-1,4 glucan phosphorylase (EC 2.4.1.1)	VIT_06s0004g06020	D7SK33_VITVI	20	34,80	95,49	73,33	1,60E+09
-879	-5,58	4,81	5,42	5,58	Vitis vinifera	Pyruvate dehydrogenase E1 component subunit alpha (EC 1.2.4.1)	VIT_09s0018g01940	A5B2Z7_VITVI	13	30,70	43,93	16,29	2,57E+08
-879	-5,88	5,75	5,38	5,88	Vitis vinifera	Putative aquaporin	delta-TIP	Q9FS46_VITVI	2	9,20	25,48	12,57	3,36E+09
-879	-5,62	4,86	5,62	5,43	Vitis vinifera	Cysteine synthase (EC 2.5.1.47)	VITISV_033255	A5AFH5_VITVI	7	32,00	34,37	98,48	9,97E+08
-879	-5,07	4,64	4,79	5,07	Vitis vinifera	40S ribosomal protein S3a	VIT_05s0020g03900	D7T724_VITVI	9	42,50	29,90	97,74	1,49E+09
-879	-5,17	4,08	4,55	5,17	Vitis vinifera	PKS_ER domain-containing protein	VIT_09s0002g08480	D7TZC8_VITVI	9	51,70	35,21	96,57	9,14E+08
-879	-5,15	4,49	4,83	5,15	Vitis vinifera	Plastoquinol--plastocyanin reductase (EC 7.1.1.6)	VIT_19s0014g03850	A5BX41_VITVI	4	23,20	24,08	21,16	2,93E+08
-879	-5,69	3,96	4,97	5,69	Vitis vinifera	Probable oxygen-evolving enhancer protein 2 (Fragment	psbP1	A5B1D3_VITVI	8	51,70	27,79	168,80	3,26E+09
-879	-5,49	3,79	4,56	5,49	Vitis vinifera	Uncharacterized protein	VIT_00s0207g00210	F6I229_VITVI	14	54,50	33,23	259,69	3,84E+09
-879	-5,40	4,99	4,98	5,40	Vitis vinifera	Ribosomal_L18e/L15P domain-containing protein	VIT_05s0077g02070	F6H6W6_VITVI	6	41,50	16,61	12,37	2,80E+08
-879	-5,31	5,31	4,87	4,60	Vitis vinifera	Expansin	VIT_06s0004g04860	F6GV05_VITVI	2	9,90	27,17	25,25	3,77E+08
-879	-5,66	5,66	3,53	4,65	Vitis vinifera	40S ribosomal protein S6	VIT_07s0191g00060	D7TRL3_VITVI	5	23,30	28,32	12,83	6,31E+08
-879	-5,03	4,90	4,83	5,03	Vitis vinifera	Uncharacterized protein	VIT_13s0019g02350	D7TLM6_VITVI	4	33,20	28,60	11,79	3,51E+08
-879	-5,80	5,80	3,79	4,91	Vitis vinifera	Thioredoxin-dependent peroxiredoxin (EC 1.11.1.24)	VIT_11s0016g00560	D7TCA6_VITVI	7	36,40	23,46	42,98	6,64E+08
-879	-5,65	5,65	5,15	4,47	Vitis vinifera	Ribulose bisphosphate carboxylase small chain (EC 4.1.1.39)	VIT_17s0000g03690	A5C718_VITVI	10	55,00	20,43	37,46	3,44E+09
-879	-5,39	5,29	4,81	5,39	Vitis vinifera	Ribosomal_L18e/L15P domain-containing protein	VIT_00s0225g00020	A5BKX2_VITVI	4	34,20	16,36	11,38	5,86E+08
-879	-5,36	5,18	4,08	5,36	Vitis vinifera	Tyrosinase_Cu-bd domain-containing protein	VITISV_044225	A5C172_VITVI	25	54,00	67,39	323,31	2,89E+09
-879	-5,26	5,26	4,84	4,42	Vitis vinifera	Ribulose bisphosphate carboxylase large chain (EC 4.1.1.39)	VITISV_025530	A5BIT1_VITVI	17	45,30	50,68	323,31	3,48E+09
-879	-5,26	3,11	5,26	4,52	Vitis vinifera	Aquaporin PIP22	PIP2;2	Q0MX13_VITVI	7	40,50	29,60	128,56	1,93E+09
-879	-4,84	4,03	4,84	3,54	Vitis vinifera	Uncharacterized protein	VIT_16s0039g00970	F6HEA4_VITVI	5	27,50	27,03	8,41	2,80E+08
-879	-4,63	3,83	4,63	4,11	Vitis vinifera	PHD-type domain-containing protein	VIT_07s0151g00870	F6HI46_VITVI	6	40,80	17,62	49,09	1,33E+09
-879	-4,63	3,39	4,63	3,89	Vitis vinifera	Pyruvate decarboxylase (EC 4.1.1.1)	VIT_06s0004g06900	F6GUP8_VITVI	18	44,90	62,42	134,45	3,02E+09
-879	-4,95	3,93	4,95	4,67	Vitis vinifera	Peptidyl-prolyl cis-trans isomerase (PPIase) (EC 5.2.1.8)	VIT_07s0005g02410	D7U2S0_VITVI	2	10,70	27,40	7,26	2,66E+08
-879	-4,64	3,98	4,64	4,17	Vitis vinifera	40S ribosomal protein S7	VIT_13s0019g02240	D7TLN6_VITVI	7	52,90	21,96	38,53	7,26E+08
-879	-4,84	3,50	4,84	2,82	Vitis vinifera	Acetyl-CoA carboxylase (EC 6.4.1.2)	VIT_18s0001g04980	F6H0V3_VITVI	14	9,90	242,54	30,38	6,30E+08
-879	-4,51	3,67	4,51	2,66	Vitis vinifera	Pyruvate decarboxylase (EC 4.1.1.1)	VIT_08s0217g00100	F6GY71_VITVI	12	32,50	62,27	47,36	1,26E+09
-879	-4,27	4,27	3,97	3,00	Vitis vinifera	Uncharacterized protein	VIT_09s0054g00030	F6GYQ5_VITVI	9	46,10	28,49	43,44	8,23E+08
-879	-4,26	4,10	4,26	3,96	Vitis vinifera	Uncharacterized protein	VIT_15s0021g02050	F6GV85_VITVI	5	24,90	33,48	17,05	2,40E+08
-879	-4,41	4,34	4,41	3,42	Vitis vinifera	Flavonoid 3',5'-methyltransferase (EC 2.1.1.267) (Anthocyanin-O-methyltransferase) (VvAOMT)	FAOMT	FAOMT_VITVI	6	41,70	26,44	96,42	1,56E+09
-879	-4,31	4,31	3,82	3,66	Vitis vinifera	Glycine cleavage system P protein (EC 1.4.4.2)	VIT_04s0008g01300	F6H3P2_VITVI	6	8,60	112,81	19,76	2,79E+08
-879	-3,99	3,99	3,72	3,96	Vitis vinifera	40S ribosomal protein S24	VIT_07s0151g00900	D7TDY2_VITVI	4	35,00	15,71	25,29	5,88E+08
-879	-4,54	4,54	4,03	4,07	Vitis vinifera	Ribos_L4_asso_C domain-containing protein	VIT_08s0040g03200	F6HQV3_VITVI	14	43,60	44,77	64,26	4,70E+08
-879	-4,64	4,64	3,91	3,94	Vitis vinifera	Ribos_L4_asso_C domain-containing protein	VIT_06s0004g00360	F6GUN5_VITVI	13	42,20	44,56	11,18	3,90E+08
-879	-4,55	4,54	3,84	4,55	Vitis vinifera	Ribosomal_L30 domain-containing protein	VIT_09s0054g00090	F6GYQ1_VITVI	7	35,40	28,69	27,31	4,08E+08
-879	-5,06	4,79	5,06	3,85	Vitis vinifera	S-AdoMet_synt_C domain-containing protein	VITISV_004113	A5AP54_VITVI	14	56,00	42,93	120,97	1,08E+09
-879	-4,77	4,77	4,32	4,05	Vitis vinifera	Uncharacterized protein	VIT_14s0108g00850	F6H5T8_VITVI	3	27,50	13,75	9,49	4,87E+08
-879	-4,84	4,84	4,49	3,23	Vitis vinifera	SHSP domain-containing protein	VIT_13s0019g03170	F6HNM6_VITVI	3	25,20	18,19	36,83	1,15E+09
-879	-4,59	4,38	3,76	4,59	Vitis vinifera	Importin subunit alpha	VIT_14s0060g00930	D7UA50_VITVI	14	42,30	58,59	137,12	3,52E+08
-879	-5,05	5,05	4,69	4,11	Vitis vinifera	60S ribosomal protein L36	VIT_15s0046g02690	F6I624_VITVI	3	27,30	12,50	4,42	3,48E+08
-879	-4,22	4,22	2,82	3,13	Vitis vinifera	Coatomer subunit epsilon	VIT_10s0003g05630	D7TKQ8_VITVI	3	12,50	32,00	10,50	1,52E+08
-879	-4,66	4,66	2,76	2,83	Vitis vinifera	S4 RNA-binding domain-containing protein	VIT_01s0026g02110	D7TNB0_VITVI	12	50,80	23,05	57,12	2,17E+09
-879	-4,47	4,47	3,61	3,15	Vitis vinifera	MI domain-containing protein	VIT_04s0008g06770	F6H343_VITVI	6	9,90	86,99	5,79	1,75E+08
-879	-4,53	4,53	3,99	2,79	Vitis vinifera	Uncharacterized protein	VIT_03s0038g03430	D7U4W1_VITVI	6	32,40	28,44	57,57	3,93E+08

Cluster n°	Isabel (log2 fold-change)	Tenta (log2 fold-change)	Sciascinoso (log2 fold-change)	Aglianico (log2 fold-change)	Organism	Protein name	Gene name	UniProt name	Peptides	Sequence coverage [%]	Mol. weight [kDa]	Score	Intensity
-879	-3,35	2,35	2,66	3,35	Vitis vinifera	Uncharacterized protein	VIT_09s0002g01760	F6HXZ5_VITVI	25	61,70	49,61	40,00	6,39E+08
-879	-3,30	2,54	2,59	3,30	Vitis vinifera	Elongation factor Tu	VIT_14s0066g00220	F6HUT0_VITVI	5	14,80	52,94	23,04	2,53E+08
-879	-2,44	2,00	1,33	2,44	Vitis vinifera	Fructose-bisphosphate aldolase (EC 4.1.2.13)	VIT_01s0011g04350	F6HFL6_VITVI	10	47,60	42,89	176,71	2,65E+09
-879	-2,86	2,33	2,25	2,86	Vitis vinifera	Glyco_hydro_32C domain-containing protein	VITISV_034734	A5B125_VITVI	21	42,80	60,00	323,31	8,84E+09
-879	-2,91	2,67	2,51	2,91	Vitis vinifera	Isopentenyl-diphosphate Delta-isomerase (EC 5.3.3.2)	VIT_04s0023g00600	F6GX19_VITVI	11	44,50	33,18	137,33	4,60E+08
-879	-3,20	2,50	3,00	3,20	Vitis vinifera	E1 ubiquitin-activating enzyme (EC 6.2.1.45)	VIT_06s0004g07060	F6GUM1_VITVI	9	12,90	120,69	40,26	7,28E+08
-879	-2,73	1,42	2,73	2,42	Vitis vinifera	Aldo_ket_red domain-containing protein	VIT_18s0001g09570	E0CPN2_VITVI	3	16,10	36,19	44,50	2,18E+08
-879	-3,17	1,42	1,62	3,17	Vitis vinifera	Gamma-ECS (EC 6.3.2.2) (Gamma-glutamylcysteine synthetase) (Glutamate--cysteine ligase, chloroplastic)	VIT_02s0025g03530	D7TVZ7_VITVI	3	9,50	59,48	9,53	1,60E+08
-879	-3,17	1,85	2,54	3,17	Vitis vinifera	Uncharacterized protein	VIT_02s0154g00080	D7TN11_VITVI	5	13,20	65,84	28,55	5,20E+08
-879	-2,44	1,65	2,21	2,44	Vitis vinifera	CCT-theta	VIT_08s0007g07460	D7TI60_VITVI	11	30,30	58,89	74,69	1,19E+09
-879	-3,44	1,85	3,00	3,44	Vitis vinifera	EGF_CA domain-containing protein	VITISV_015390	A5C8C9_VITVI	4	9,00	69,40	18,10	1,73E+08
-879	-3,20	1,78	2,49	3,20	Vitis vinifera	Uncharacterized protein	VIT_00s2436g00010	F6HDC6_VITVI	11	28,00	71,65	39,53	7,72E+08
-879	-3,08	2,35	1,91	3,08	Vitis vinifera	Phosphoinositide phospholipase C (EC 3.1.4.11)	VIT_13s0067g00890	D7T4P6_VITVI	9	24,80	67,77	26,89	7,00E+08
-879	-2,86	2,21	1,79	2,86	Vitis vinifera	Malate dehydrogenase [NADP], chloroplastic (EC 1.1.1.82) (NADP-MDH)	VIT_13s0019g05250	D7TM20_VITVI	7	24,00	47,71	35,13	9,13E+08
-879	-3,12	2,46	3,12	3,02	Vitis vinifera	Uncharacterized protein	VIT_00s0260g00080	F6I7A5_VITVI	4	36,10	15,60	9,06	2,67E+08
-879	-3,28	1,77	1,89	3,28	Vitis vinifera	Carboxypeptidase (EC 3.4.16.-)	VIT_13s0019g05130	F6HNB7_VITVI	8	22,40	56,89	39,53	6,34E+08
-879	-2,69	2,08	1,36	2,69	Vitis vinifera	Elongation factor 1-alpha	VIT_13s0019g03920	F6HNI6_VITVI	10	32,70	49,38	65,63	1,41E+09
-879	-3,28	1,84	3,11	3,28	Vitis vinifera	Polyphenol oxidase (EC 1.10.3.1)	PPO	A0A0F7G9W3	17	32,90	67,39	90,82	1,64E+09
-879	-2,99	2,31	2,44	2,99	Vitis vinifera	Uncharacterized protein	VIT_07s0151g01000	D7TDX6_VITVI	6	37,30	22,48	44,08	1,13E+09
-879	-2,48	2,31	2,48	2,16	Vitis vinifera	Uncharacterized protein	VIT_00s0904g00010	F6H8B4_VITVI	6	34,20	24,33	77,48	2,07E+09
-879	-2,12	2,10	2,12	1,75	Vitis vinifera	PHB domain-containing protein	VIT_17s0000g05680	F6GTA6_VITVI	5	16,90	36,22	5,87	2,86E+08
-879	-3,01	3,01	2,80	2,59	Vitis vinifera	Aldo_ket_red domain-containing protein	VIT_03s0038g00800	D7U5G1_VITVI	10	42,10	34,79	24,15	5,17E+08
-879	-2,36	2,18	2,36	1,97	Vitis vinifera	Uncharacterized protein	VIT_05s0165g00230	F6HN65_VITVI	6	48,20	22,02	54,78	3,96E+08
-879	-3,27	2,90	3,27	1,93	Vitis vinifera	Glycine hydroxymethyltransferase (EC 2.1.2.1)	VIT_00s0211g00070	D7T5B6_VITVI	13	33,20	57,21	60,20	2,69E+08
-879	-2,50	2,50	1,72	1,93	Vitis vinifera	UV-B receptor 1	UVR1	M4N7R5_VITVI	3	9,90	47,75	6,40	1,63E+08
-879	-2,80	2,48	2,80	2,41	Vitis vinifera	Ribosomal protein L15	VIT_00s0551g00020	F6HD52_VITVI	3	21,80	24,72	13,84	2,08E+08
-879	-2,91	2,91	2,61	1,56	Vitis vinifera	Uncharacterized protein	VIT_06s0004g00240	D7SLM9_VITVI	14	32,60	64,61	113,97	8,64E+08
-879	-3,20	2,78	3,20	2,28	Vitis vinifera	Ribosomal_L28e domain-containing protein	VIT_09s0002g02560	A5C7R4_VITVI	4	34,20	16,44	20,32	8,49E+08
-879	-3,11	2,70	3,11	1,98	Vitis vinifera	Cytochrome b5 heme-binding domain-containing protein	VIT_18s0001g09400	A5B9D6_VITVI	3	32,70	16,29	16,73	3,37E+08
-879	-2,29	2,29	2,27	1,56	Vitis vinifera	Polyadenylate-binding protein (PABP)	VIT_03s0063g00070	F6HQ88_VITVI	7	15,60	70,68	36,86	1,48E+08
-879	-3,58	3,58	3,05	1,93	Vitis vinifera	Isoflavone reductase-like protein 6	ifrl6	Q3KN67_VITVI	13	47,10	33,93	53,23	7,88E+08
-879	-3,58	3,58	3,05	1,93	Vitis vinifera	NmrA domain-containing protein	VIT_03s0038g04700	D7U4J3_VITVI	13	47,10	33,93	53,23	7,88E+08
-879	-3,48	3,48	3,10	2,80	Vitis vinifera	Peptidylprolyl isomerase (EC 5.2.1.8)	VIT_00s0769g00010	D7TGC8_VITVI	5	11,20	64,04	10,78	2,19E+08
-879	-3,68	3,16	3,68	2,83	Vitis vinifera	Uncharacterized protein	VIT_18s0001g00590	F6HWA0_VITVI	16	37,40	57,68	80,45	1,87E+09
-879	-3,76	3,76	3,49	2,53	Vitis vinifera	PABS domain-containing protein	VIT_01s0026g00240	A5BHF8_VITVII	3	20,50	32,58	10,52	1,85E+08
-879	-3,68	3,68	3,22	3,15	Vitis vinifera	Adenylosuccinate synthetase, chloroplastic (AMPSase) (AdSS) (EC 6.3.4.4) (IMP--aspartate ligase)	PURA	A5B050_VITVI	5	5,20	152,02	18,31	4,09E+08
-879	-3,82	3,31	3,82	2,55	Vitis vinifera	Aspartate aminotransferase (EC 2.6.1.1)	VIT_04s0008g06040	F6H3U9_VITVI	18	50,40	50,82	86,57	1,97E+09
-879	-3,47	3,07	3,47	3,17	Vitis vinifera	DLH domain-containing protein	VIT_07s0104g00410	D7TPC1_VITVI	9	57,80	25,54	17,05	5,14E+08
-879	-3,70	3,11	3,70	3,15	Vitis vinifera	Cytochrome b5 heme-binding domain-containing protein	VIT_07s0005g06290	A5ATD8_VITVI	4	42,90	14,67	21,21	4,81E+08
-879	-3,59	2,84	2,70	3,59	Vitis vinifera	Delta-1-pyrroline-5-carboxylate synthase [Includes: Glutamate 5-kinase (GK) (EC 2.7.2.11) (Gamma-glutamyl kinase)	VIT_08s0007g01060	D7THH7_VITVI	6	14,80	77,77	22,60	1,96E+08
-879	-3,59	3,59	2,59	2,80	Vitis vinifera	Aminomethyltransferase (EC 2.1.2.10) (Glycine cleavage system T protein)	VIT_10s0116g01540	F6H7I9_VITVI	10	34,10	44,31	67,67	1,38E+09
-879	-3,53	3,44	2,80	3,53	Vitis vinifera	Protein-serine/threonine phosphatase (EC 3.1.3.16)	VIT_04s0008g06720	F6H341_VITVI	5	25,70	43,18	19,61	2,09E+08
-879	-3,66	3,02	2,44	3,66	Vitis vinifera	Serine hydroxymethyltransferase (EC 2.1.2.1)	VIT_05s0029g00310	F6GWF3_VITVI	22	72,80	51,91	302,96	6,90E+09
-879	-3,64	3,64	2,41	2,69	Vitis vinifera	Ribosomal_L18e/L15P domain-containing protein	VIT_05s0077g02060	F6H6W5_VITVI	7	39,00	20,90	29,05	4,90E+08
-879	-3,39	3,39	2,40	3,16	Vitis vinifera	EMB1873 protein (Fragment)	*N/A*	A0A097PRR3_VITVI	5	19,30	41,53	19,20	4,64E+08
-879	-4,19	2,64	3,57	4,19	Vitis vinifera	ATP-dependent 6-phosphofructokinase (ATP-PFK) (Phosphofructokinase) (EC 2.7.1.11) (Phosphohexokinase)	PFK	F6I7K1_VITVI	3	7,80	51,28	5,03	1,71E+08
-879	-4,36	2,57	2,91	4,36	Vitis vinifera	Aldo_ket_red domain-containing protein	VITISV_029327	A5AD51_VITVI	9	39,50	34,78	19,26	5,65E+08

Cluster n°	Isabel (log2 fold-change)	Tenta (log2 fold-change)	Sciascinoso (log2 fold-change)	Aglianico (log2 fold-change)	Organism	Protein name	Gene name	UniProt name	Peptides	Sequence coverage [%]	Mol. weight [kDa]	Score	Intensity
-879	-3,69	3,26	3,62	3,69	Vitis vinifera	Elongation factor G, chloroplastic (cEF-G)	VIT_00s0323g00080	F6HHS2_VITVI	5	9,50	85,31	10,86	1,88E+08
-879	-4,17	3,00	3,95	4,17	Vitis vinifera	Uncharacterized protein	VIT_05s0051g00720	D7TS82_VITVI	10	35,60	48,62	81,51	1,05E+09
-879	-4,20	3,07	3,07	4,20	Vitis vinifera	Coatomer subunit gamma	VIT_03s0063g02450	D7TQ06_VITVI	20	33,40	98,62	93,96	1,51E+09
-879	-4,59	3,76	3,26	4,59	Vitis vinifera	Ferritin (EC 1.16.3.1)	VITISV_041389	A5BV73_VITVI	10	43,40	29,49	77,20	2,61E+09
-879	-3,95	3,44	3,95	3,87	Vitis vinifera	Chorismate synthase (EC 4.2.3.5)	VIT_13s0019g04190	D7TMA3_VITVI	9	32,60	47,18	28,16	4,77E+08
-879	-4,09	3,31	4,09	3,97	Vitis vinifera	UDP-glucose 6-dehydrogenase (EC 1.1.1.22)	VITISV_021451	A5BDX4_VITVI	15	45,00	52,96	46,23	6,03E+08
-879	-4,04	2,82	3,00	4,04	Vitis vinifera	Photosystem I (EC 1.97.1.12)	VITISV_019459	A5BDX5_VITVI	10	18,80	82,39	73,55	1,55E+09
-879	-4,92	3,12	3,01	4,92	Vitis vinifera	Uncharacterized protein	VITISV_011599	A5AQ45_VITVI	13	50,50	34,63	33,39	3,34E+08
-879	-3,86	2,56	3,31	3,86	Vitis vinifera	Glutamate dehydrogenase	VIT_08s0007g07400	F6HLB4_VITVI	14	45,10	52,50	94,63	2,52E+08
-879	-4,58	2,54	4,58	3,69	Vitis vinifera	Alcohol dehydrogenase 3 (EC 1.1.1.1)	ADH3	Q9FZ00_VITVI	12	50,30	41,24	40,13	1,27E+09
-879	-3,69	2,57	3,67	3,69	Vitis vinifera	Uncharacterized protein	VITISV_038729	D7SHM5_VITVI	22	37,40	89,31	19,51	4,23E+08
-879	-3,71	2,52	3,71	3,37	Vitis vinifera	NAD_binding_2 domain-containing protein	VIT_00s2081g00010	D7ST63_VITVI	7	42,60	30,83	50,50	7,15E+08
-879	-3,73	2,07	3,73	3,40	Vitis vinifera	Elongation factor 1-alpha	VIT_06s0004g03220	A5AFS1_VITVI	12	43,40	49,34	233,54	4,85E+09
-879	-3,73	2,07	3,73	3,40	Vitis vinifera	Tr-type G domain-containing protein	VIT_00s1584g00010	F6GTY8_VITVI	12	43,40	49,34	233,54	4,85E+09
-879	-3,47	2,36	3,47	3,46	Vitis vinifera	Glutamine synthetase (EC 6.3.1.2)	VIT_01s0011g02200	A5AP38_VITVI	15	67,70	39,02	186,71	3,95E+09
-879	-6,82	6,51	6,35	6,82	Vitis vinifera	Uncharacterized protein	VITISV_018238	A5AM45_VITVI	4	25,90	26,48	40,89	7,02E+08
-879	-7,30	7,30	7,16	6,77	Vitis vinifera	Methyltransferase (EC 2.1.1.-)	VITISV_002505	A5B620_VITVI	10	46,40	38,88	319,49	2,19E+10
-879	-7,29	6,44	7,29	7,23	Vitis vinifera	Ribosomal_L18_c domain-containing protein	VIT_05s0094g00870	F6HAX5_VITVI	3	11,30	34,47	5,09	5,84E+08
-879	-7,76	7,74	7,76	6,19	Vitis vinifera	Plastocyanin-like domain-containing protein	VIT_10s0116g01600	E0CVR8_VITVI	16	39,50	60,23	179,05	2,26E+09
-879	-7,83	6,38	7,83	7,17	Vitis vinifera	Phosphopyruvate hydratase (EC 4.2.1.11)	VIT_16s0022g01770	D7T227_VITVI	27	74,40	48,11	323,31	1,69E+10
-879	-7,87	6,31	7,87	6,66	Vitis vinifera	Naringenin,2-oxoglutarate 3-dioxygenase (EC 1.14.11.9) (FHT) (Flavanone-3-hydroxylase) (F3H)	F3H	FL3H_VITVI	17	67,20	40,80	226,61	1,95E+09
-879	-7,15	5,64	7,15	6,95	Vitis vinifera	Uncharacterized protein	VIT_01s0127g00560	F6HHU9_VITVI	24	53,00	64,99	323,31	3,40E+09
-879	-7,59	7,36	7,59	6,18	Vitis vinifera	Glyceraldehyde-3-phosphate dehydrogenase (EC 1.2.1.-)	VIT_14s0068g00680	A5BCS8_VITVI	9	26,20	43,16	55,59	1,27E+09
-879	-7,73	7,73	7,65	6,06	Vitis vinifera	Glyceraldehyde-3-phosphate dehydrogenase (EC 1.2.1.-)	VITISV_021576	A5AX39_VITVI	14	36,20	48,12	117,06	1,56E+09
-879	-6,59	6,07	6,59	6,46	Vitis vinifera	Ribosomal_S13_N domain-containing protein	VIT_16s0022g01590	A5B3K9_VITVI	3	23,80	17,18	8,96	4,88E+08
-842	-1,83	0,00	0,00	1,83	Vitis vinifera	PAP_fibrillin domain-containing protein	VIT_02s0012g02050	F6HT52_VITVI	2	6,50	40,77	3,54	1,58E+08
-842	-1,91	0,00	0,00	1,91	Vitis vinifera	Eukaryotic translation initiation factor 3 subunit D (eIF3d) (Eukaryotic translation initiation factor 3 subunit 7) (eIF-3-zeta)	VIT_00s0880g00020	F6HJU0_VITVI	6	21,50	64,52	25,22	3,12E+08
-842	-1,50	0,00	0,00	1,50	Vitis vinifera	Uncharacterized protein	VIT_12s0028g01260	F6H560_VITVI	5	19,70	33,42	3,27	2,20E+08
-842	-1,61	0,00	0,00	1,61	Vitis vinifera	Dolichyl-diphosphooligosaccharide--protein glycosyltransferase subunit 1	VIT_18s0122g00890	D7UDD5_VITVI	11	29,20	68,86	20,23	6,09E+08
-842	-1,35	0,00	0,00	1,35	Vitis vinifera	CAAX prenyl protease (EC 3.4.24.84)	VIT_15s0046g02040	D7UCF7_VITVI	5	15,80	48,38	6,55	7,49E+08
-842	-1,48	0,00	0,00	1,48	Vitis vinifera	Importin N-terminal domain-containing protein	VIT_03s0038g02830	D7U509_VITVI	5	11,40	99,67	18,37	1,92E+08
-842	-2,10	0,00	0,00	2,10	Vitis vinifera	Uncharacterized protein	VIT_04s0008g05330	F6H316_VITVI	4	14,30	48,35	7,75	2,00E+08
-842	-1,57	0,00	0,00	1,57	Vitis vinifera	Elongation factor 1-alpha	VIT_08s0040g02330	A5C4C2_VITVI	10	32,70	49,34	52,54	8,18E+08
-842	-1,71	0,00	0,00	1,71	Vitis vinifera	Peptidyl-prolyl cis-trans isomerase (PPIase) (EC 5.2.1.8)	VIT_14s0081g00700	A5B709_VITVI	2	12,10	18,77	5,81	3,02E+08
-842	-1,62	0,00	0,00	1,62	Vitis vinifera	Protein ROOT HAIR DEFECTIVE 3 homolog (EC 3.6.5.-) (Protein SEY1 homolog)	VITISV_010618	A5B6W0_VITVI	5	7,40	89,93	4,02	4,07E+08
-842	-2,03	0,00	0,00	2,03	Vitis vinifera	Thioredoxin	VIT_18s0001g13250	A5AML5_VITVI	5	52,60	12,82	21,02	2,36E+09
-842	-1,36	0,00	0,00	1,36	Vitis vinifera	Cytochrome f	VITISV_017654	A5ANE5_VITVI	9	40,80	31,56	50,74	7,65E+08
-842	-1,83	0,00	0,00	1,83	Vitis vinifera	V-type proton ATPase subunit	VIT_14s0108g00080	A5ACR7_VITVI	6	26,80	40,67	106,73	3,82E+08
-842	0,00	-1,39	0,00	1,39	Vitis vinifera	Uncharacterized protein	VIT_08s0056g00620	F6HMT9_VITVI	8	35,70	26,03	50,64	5,08E+08
-842	0,00	-1,73	-1,43	1,73	Vitis vinifera	Uncharacterized protein	VIT_01s0137g00180	F6GVK2_VITVI	6	23,40	35,45	8,01	3,09E+08
-842	0,00	-1,41	0,00	1,41	Vitis vinifera	Uncharacterized protein	VIT_06s0004g00500	F6GUN0_VITVI	6	8,90	123,39	13,43	1,66E+08
-842	0,00	-1,94	0,00	1,94	Vitis vinifera	Aldo_ket_red domain-containing protein	VITISV_033356	A5C8G1_VITVI	9	36,80	35,36	19,25	2,29E+08
-842	0,00	-1,38	0,00	1,38	Vitis vinifera	Uncharacterized protein	VIT_15s0048g01890	D7U7J5_VITVI	2	13,60	25,76	11,52	1,42E+08
-842	0,00	-1,62	0,00	1,62	Vitis vinifera	Uncharacterized protein	VIT_01s0150g00400	D7TFL3_VITVI	16	43,30	60,62	120,79	2,40E+09
-842	0,00	-1,72	0,00	1,72	Vitis vinifera	Uncharacterized protein	VIT_16s0022g02100	D7T1V9_VITVI	2	16,20	21,07	45,63	1,05E+08
-842	0,00	-1,93	0,00	1,93	Vitis vinifera	Uncharacterized protein	VIT_06s0009g03520	D7T153_VITVI	2	6,70	37,77	3,37	5,94E+07
-842	0,00	-1,72	0,00	1,72	Vitis vinifera	AAA domain-containing protein	VIT_04s0008g04690	D7U0U9_VITVI	8	29,00	47,10	54,41	5,52E+08
-842	0,00	-1,55	0,00	1,55	Vitis vinifera	ADH_zinc_N domain-containing protein	VIT_00s0371g00040	F6HRU5_VITVI	8	36,70	38,85	11,28	1,23E+08
-842	0,00	-2,28	0,00	2,28	Vitis vinifera	Histone domain-containing protein	VIT_07s0191g00130	F6HRP4_VITVI	3	35,30	15,41	21,88	3,13E+08

Cluster n°	Isabel (log2 fold-change)	Tenta (log2 fold-change)	Sciascinoso (log2 fold-change)	Aglianico (log2 fold-change)	Organism	Protein name	Gene name	UniProt name	Peptides	Sequence coverage [%]	Mol. weight [kDa]	Score	Intensity
-842	0,00	-1,55	0,00	1,55	Vitis pseudoreticulata (Chinese wild grapevine);Vitis vinifera	RRM domain-containing protein	VIT_03s0063g02610	F6HQM5_VITVI	4	35,20	16,33	161,72	4,44E+09
-842	0,00	-1,55	0,00	1,55	Vitis vinifera	Peptidyl-prolyl cis-trans isomerase (PPIase) (EC 5.2.1.8)	VIT_01s0146g00110	D7U5Y4_VITVI	2	18,30	19,69	15,53	1,26E+08
-842	-1,38	-2,19	0,00	2,19	Vitis vinifera	Phosphotransferase (EC 2.7.1.-)	VIT_11s0016g03070	F6HGY3_VITVI	15	52,10	51,22	78,21	1,27E+09
-842	-2,02	-1,75	0,00	2,02	Vitis vinifera	UBX domain-containing protein	VIT_02s0025g04050	F6HUF2_VITVI	8	21,50	68,32	35,14	4,17E+08
-842	-1,33	-1,92	0,00	1,92	Vitis vinifera	Proteasome subunit beta (EC 3.4.25.1)	VIT_15s0048g00900	D7U7S6_VITVI	8	58,70	24,63	48,61	9,39E+08
-842	-2,08	-2,18	0,00	2,18	Vitis vinifera	AB hydrolase-1 domain-containing protein	VITISV_042701	A5ARM5_VITVI	5	21,80	42,78	19,12	2,53E+08
-842	-2,36	-2,35	0,00	2,36	Vitis vinifera	Uncharacterized protein	VIT_11s0016g01920	D7TCM7_VITVI	26	73,40	46,88	323,31	1,67E+08
-842	-2,47	-2,47	0,00	2,47	Vitis vinifera	NAD(P)H dehydrogenase (quinone) (EC 1.6.5.2)	VIT_00s0271g00110	D7UE46_VITVI	8	56,70	21,74	7,13	2,57E+08
-842	-1,82	-1,87	0,00	1,87	Vitis vinifera	CCT-alpha (T-complex protein 1 subunit alpha)	VIT_15s0021g02230	D7SLU3_VITVI	12	30,80	59,17	56,81	6,50E+08
-842	-2,17	-1,86	0,00	2,17	Vitis vinifera	TPR_REGION domain-containing protein	VIT_08s0058g00820	D7SR32_VITVI	8	34,30	32,59	19,41	6,03E+08
-842	-1,98	-1,39	0,00	1,98	Vitis vinifera	Uncharacterized protein	VIT_08s0007g01660	F6HKX8_VITVI	5	16,40	47,74	22,37	2,93E+08
-842	-2,43	-1,70	0,00	2,43	Vitis vinifera	Coatomer subunit delta	VIT_01s0011g01570	D7T9L8_VITVI	12	28,60	58,28	50,85	1,01E+09
-842	-1,95	-1,67	0,00	1,95	Vitis vinifera	Sulfurtransferase	VIT_19s0085g00260	A5B8K7_VITVI	7	20,80	41,82	33,72	5,25E+08
**Secondary Clusters**													
-793	-6,49	-7,67	7,67	-6,24	Vitis vinifera	Glyceraldehyde-3-phosphate dehydrogenase (EC 1.2.1.-)	GAPDH	E3TBN6_VITVI	15	65,00	36,92	277,39	5,33E+09
-793	-6,93	-5,20	6,93	-6,06	Vitis vinifera	Nascent polypeptide-associated complex subunit beta	VIT_17s0053g00620	D7TXR6_VITVI	8	67,50	17,17	56,55	7,95E+08
-793	-6,33	-5,98	7,21	-7,21	Vitis amurensis;Vitis hybrid cultivar;Vitis vinifera	Glutathione transferase (EC 2.5.1.18)	VITISV_026911	A5BM22_VITVI	9	50,70	24,22	82,03	1,31E+09
-793	-6,25	-6,61	6,61	-5,87	Vitis vinifera	Bet_v_1 domain-containing protein	VIT_01s0011g05120	A5AKK0_VITVI	12	84,80	17,10	323,31	2,14E+10
-793	-5,28	-5,08	5,28	-4,96	Vitis vinifera	Anthocyanin acyltransferase	ANAT	D7TU67_VITVI	8	22,00	47,50	14,71	2,55E+08
-793	-6,05	-7,28	7,28	-5,73	Vitis vinifera	Uncharacterized protein	VIT_12s0059g02390	D7TEN0_VITVI	12	48,60	38,33	115,51	2,28E+09
-880	6,33	6,42	-5,14	-6,42	Vitis vinifera	Uncharacterized protein	VIT_07s0005g02710	F6HZG4_VITVI	11	38,30	50,63	60,24	1,18E+09
-880	6,19	4,78	-4,08	-6,19	Vitis vinifera	NAD(P)-bd_dom domain-containing protein	VIT_08s0007g00530	F6HL96_VITVI	7	30,10	36,06	51,96	1,19E+09
-880	6,64	4,31	-6,64	-6,57	Vitis vinifera	Uncharacterized protein	VIT_19s0015g02680	F6I511_VITVI	14	40,60	25,59	66,41	2,55E+09
-880	7,09	4,78	-5,16	-7,09	Vitis vinifera	ACB domain-containing protein	VIT_02s0033g01330	D7U3Z9_VITVI	4	58,20	10,24	28,74	5,45E+08
-880	7,71	6,11	-5,67	-7,71	Vitis vinifera	14_3_3 domain-containing protein;14-3-3 protein	*N/A*	C1KG73_VITVI	11	51,20	28,65	185,48	3,33E+09
-880	7,09	4,72	-5,48	-7,09	Vitis vinifera	Glutaredoxin domain-containing protein	VIT_14s0066g00960	A5B8K3_VITVI	8	95,60	11,81	166,46	3,17E+09
-880	3,77	3,60	-2,68	-3,77	Vitis vinifera	Putative ripening-related protein	grip32	Q9M4H2_VITVI	6	51,60	13,55	183,68	6,19E+09
-880	3,85	2,25	-2,11	-3,85	Vitis vinifera	Isoflavone reductase-like protein 5	ifrl5	Q3KN68_VITVI	17	78,40	33,89	157,40	3,42E+09
-880	3,66	2,39	-3,66	-2,52	Vitis vinifera	Lipocln_cytosolic_FA-bd_dom domain-containing protein	VIT_11s0206g00120	D7SSP3_VITVI	9	49,70	21,54	134,81	4,29E+09
-880	3,78	3,49	-2,04	-3,78	Vitis vinifera	Uncharacterized protein	VIT_03s0017g01720	D7TUC8_VITVI	15	49,50	58,34	221,91	2,63E+09
-880	3,99	3,47	-2,70	-3,99	Vitis vinifera	Uncharacterized protein	VIT_17s0000g03620;	D7SIZ7_VITVI	8	47,40	26,32	99,90	1,47E+09
-880	3,85	3,18	-2,18	-3,85	Vitis vinifera	3-hydroxy-3-methylglutaryl coenzyme A synthase (HMG-CoA synthase) (EC 2.3.3.10)	VIT_02s0025g04580	D7TW88_VITVI	6	14,70	51,00	12,26	7,07E+08
-880	2,92	4,31	-4,05	-4,31	Vitis cinerea var. helleri x Vitis rupestris	Putative aquaporin PIP1-3	*N/A*	*N/A*	11	48,10	30,71	323,31	5,84E+09
-880	2,14	3,93	-3,93	-2,63	Vitis vinifera	Uncharacterized protein	VITISV_022891	A5C0K8_VITVI	8	28,00	45,96	25,57	4,01E+08
-880	4,39	3,59	-3,34	-4,39	Vitis vinifera	Inorganic diphosphatase (EC 3.6.1.1)	VIT_03s0088g01290	F6HBJ7_VITVI	2	14,70	16,78	10,89	1,85E+08
-880	3,86	4,38	-2,39	-4,38	Vitis vinifera	Lactoylglutathione lyase (EC 4.4.1.5) (Glyoxalase I)	VIT_10s0116g01660	F6H7L5_VITVI	5	15,80	46,68	28,77	4,12E+08
-880	4,21	3,93	-3,07	-4,21	Vitis vinifera	GOLD domain-containing protein	VIT_14s0108g01140	F6H5V7_VITVI	3	21,00	23,72	9,29	1,66E+08
-880	4,29	4,03	-4,29	-3,43	Vitis vinifera	Lactoylglutathione lyase (EC 4.4.1.5) (Glyoxalase I)	VIT_06s0061g00460	D7SND0_VITVI	8	40,00	26,49	23,50	1,10E+09
-880	4,09	4,09	-4,09	-4,01	Vitis vinifera	Cytochrome b-c1 complex subunit Rieske, mitochondrial (EC 7.1.1.8)	VIT_01s0150g00160	D7TFJ1_VITVI	3	11,00	29,74	21,04	3,59E+08
-880	4,75	2,97	-4,40	-4,75	Vitis vinifera	Amine oxidase (EC 1.4.3.-)	VIT_00s1937g00010	F6HWY7_VITVI	5	10,50	81,72	7,96	1,18E+08
-880	4,42	3,22	-4,42	-3,77	Vitis vinifera	Pectinesterase (EC 3.1.1.11)	VIT_11s0016g00290	F6HGZ1_VITVI	6	13,80	62,85	35,91	3,88E+08
-880	4,50	3,20	-4,50	-3,15	Vitis vinifera	Proteasome subunit alpha type	VIT_01s0011g01960	D7T9I6_VITVI	7	37,90	25,58	71,55	2,52E+09

Cluster n°	Isabel (log2 fold-change)	Tenta (log2 fold-change)	Sciascinoso (log2 fold-change)	Aglianico (log2 fold-change)	Organism	Protein name	Gene name	UniProt name	Peptides	Sequence coverage [%]	Mol. weight [kDa]	Score	Intensity
-880	5,39	4,16	-3,49	-5,39	Vitis vinifera	Uncharacterized protein	VIT_01s0026g01340	F6HPH1_VITVI	4	17,70	25,63	19,32	5,23E+08
-880	4,63	3,45	-3,31	-4,63	Vitis vinifera	Calmodulin	VIT_17s0000g00580	A5BNP0_VITVI	7	51,00	16,95	145,47	8,19E+08
-880	5,08	3,70	-5,08	-5,08	Vitis vinifera	Aldehyde dehydrogenase	VITISV_044129	A5AQ13_VITVI	9	18,10	59,09	41,82	5,84E+08
-880	5,15	3,14	-5,15	-4,54	Vitis vinifera	Progesterone 5-beta-reductase (EC 1.3.99.6)	VIT_03s0091g00450	F6H675_VITVI	12	43,80	44,53	65,34	1,79E+09
-880	4,61	5,26	-5,26	-3,36	Vitis quinquangularis;Vitis vinifera	Aldedh domain-containing protein	VIT_01s0026g00220	F6HPN2_VITVI	23	59,90	58,33	323,31	3,47E+09
-880	5,53	5,13	-5,53	-5,53	Vitis vinifera	Pyruvate dehydrogenase E1 component subunit alpha (EC 1.2.4.1)	VIT_01s0026g00990	F6HPJ1_VITVI	14	33,20	43,70	40,47	2,14E+08
-880	5,55	4,61	-5,55	-5,34	Vitis vinifera	Abhydrolase_3 domain-containing protein	VIT_06s0009g00980	F6HA78_VITVI	8	41,00	35,13	28,00	5,53E+08
-880	4,65	5,05	-4,54	-5,05	Vitis vinifera	Uncharacterized protein	VIT_14s0060g02140	F6I4M7_VITVI	3	16,60	25,82	11,75	1,42E+08
-880	5,83	4,35	-4,97	-5,83	Vitis pseudoreticulata (Chinese wild grapevine);Vitis vinifera	PAP_fibrillin domain-containing protein	VIT_08s0007g06480	A5B0L5_VITVI	9	34,70	34,19	70,84	1,46E+09
-880	4,88	4,77	-4,88	-4,73	Vitis vinifera	Bet_v_1 domain-containing protein	VITISV_007417	A5B0T9_VITVI	4	33,30	17,29	15,52	3,34E+08
-880	6,91	8,27	-8,27	-6,91	Vitis vinifera	Mannan endo-1,4-beta-mannosidase (EC 3.2.1.78)	VIT_18s0001g12830	E0CQG0_VITVI	12	43,60	48,97	185,88	2,65E+09
-878	2,19	-3,18	3,18	-1,85	Vitis vinifera	Phosphoenolpyruvate carboxykinase (ATP) (EC 4.1.1.49)	VIT_00s1995g00010	F6HX00_VITVI	14	42,20	49,63	101,85	1,69E+09
-878	3,28	-3,62	3,62	-2,99	Vitis vinifera	Uncharacterized protein	VIT_00s2840g00010	F6H8G5_VITVI	9	61,40	25,62	95,31	1,84E+09
-878	2,86	-2,49	3,81	-3,81	Vitis vinifera	Probable 6-phosphogluconolactonase	VIT_16s0098g01200	E0CVA1_VITVI	10	51,40	27,98	71,17	1,58E+09
-878	2,59	-3,65	3,65	-2,70	Vitis vinifera	Uncharacterized protein	VIT_18s0001g01640	E0CR04_VITVI	14	31,80	70,88	38,32	1,14E+09
-878	4,00	-4,00	2,96	-3,99	Vitis aestivalis;Vitis vinifera	ANK_REP_REGION domain-containing protein	VIT_03s0038g04270	D7U4N2_VITVI	6	32,10	38,39	12,12	3,77E+08
-878	3,75	-3,12	4,96	-4,96	Vitis vinifera	Uncharacterized protein	VIT_07s0005g02010	D7U2N2_VITVI	3	13,60	21,84	6,50	1,22E+08
-878	1,47	-1,78	2,99	-2,99	Vitis vinifera	ATP-dependent 6-phosphofructokinase (ATP-PFK) (Phosphofructokinase) (EC 2.7.1.11) (Phosphohexokinase)	PFK	A5BPE1_VITVI	12	35,90	53,48	94,97	6,29E+07
-878	3,15	-2,93	1,73	-3,15	Vitis vinifera	Uncharacterized protein	VIT_18s0001g10780	A5B894_VITVI	6	40,90	26,72	75,08	8,64E+08
-878	2,97	-2,11	3,18	-3,18	Vitis vinifera	GST N-terminal domain-containing protein	VIT_06s0004g05680	F6GUW7_VITVI	4	17,90	38,20	32,28	5,82E+08
-878	2,69	-2,34	3,47	-3,47	Vitis vinifera	Chlorophyll a-b binding protein, chloroplastic	VIT_12s0055g01110	A5ASW8_VITVI	5	36,60	27,22	55,43	6,42E+08
-878	8,03	-7,93	6,80	-8,03	Vitis vinifera	Non-specific lipid-transfer protein	LTP1	Q2QCI7_VITVI	3	34,50	11,77	64,09	7,64E+09
-878	4,82	-3,85	5,04	-5,04	Vitis vinifera	Cysteine proteinase inhibitor	VIT_13s0084g00050	F6HV98_VITVI	6	52,20	22,57	28,76	7,00E+08
-878	5,50	-5,48	4,97	-5,50	Vitis vinifera	Usp domain-containing protein	VITISV_006398	A5AGB5_VITVI	4	31,10	18,07	11,68	6,25E+08
-878	8,12	-7,89	7,09	-8,12	Vitis vinifera	Isoflavone reductase-like protein 4	ifrl4	Q3KN69_VITVI	9	35,10	33,81	8,42	1,01E+08
-878	7,58	-5,64	5,45	-7,58	Vitis vinifera	Cytochrome b-c1 complex subunit 7	VIT_04s0008g05000	D7SUP9_VITVI	4	37,70	14,71	28,46	5,93E+08
-878	5,42	-5,42	3,52	-3,83	Vitis vinifera	PKS_ER domain-containing protein	VIT_00s0346g00110	D7TRS2_VITVI;	9	44,40	38,85	156,82	3,10E+08
-876	5,92	5,43	-5,97	5,97	Vitis vinifera	Uncharacterized protein	VIT_16s0098g01580	F6H7B3_VITVI	17	28,80	73,49	28,73	4,50E+08
-876	4,43	4,97	-6,49	6,49	Vitis vinifera	Cation_ATPase_N domain-containing protein	VIT_02s0488g00010	F6HD58_VITVI	24	30,80	104,81	74,83	9,30E+08
-876	5,62	5,43	-5,62	3,70	Vitis vinifera	Aa_trans domain-containing protein	VIT_01s0010g02640	F6HG53_VITVI	3	6,60	49,29	3,30	9,95E+07
-876	5,20	4,83	-5,20	4,08	Vitis vinifera	Uncharacterized protein	VIT_15s0048g00420	D7U7W7_VITVI	8	39,10	40,75	45,63	9,21E+08
-876	4,27	4,57	-5,61	5,61	Vitis vinifera	Peptidase_M1 domain-containing protein	VIT_02s0025g03270	D7TVX4_VITVI	12	36,50	54,72	65,27	1,21E+09
-876	5,45	3,91	-5,45	4,90	Vitis vinifera	Citrate synthase	VIT_12s0142g00610	D7TMQ2_VITVI	10	32,80	56,34	45,58	7,17E+08
-876	7,75	5,44	-7,75	5,80	Vitis vinifera	Uncharacterized protein	VIT_15s0021g02670	D7SLR4_VITVI	8	55,30	29,14	142,23	7,38E+09
-876	10,28	9,56	-10,28	8,55	Vitis vinifera	Chitin-binding type-1 domain-containing protein	VIT_05s0094g00350	D7T2C8_VITVI	2	5,40	46,37	46,59	1,65E+10
-876	4,09	3,26	-4,09	2,47	Vitis vinifera	Clathrin light chain	VIT_00s0370g00020	F6HWS4_VITVI	3	13,30	41,48	34,36	3,00E+08
-876	2,41	2,86	-2,86	2,25	Vitis vinifera	UBX domain-containing protein	VIT_02s0025g02340	F6HU62_VITVI	4	14,50	51,27	11,93	2,84E+08
-876	3,30	3,41	-3,41	1,85	Vitis vinifera	Uncharacterized protein	VIT_11s0016g04290	F6HH42_VITVI	7	27,10	49,56	30,65	7,51E+08
-876	4,09	3,13	-5,07	5,07	Vitis vinifera	DUF3700 domain-containing protein	VIT_05s0020g02770	F6HDM0_VITVI	5	23,20	29,53	60,51	5,79E+08
-876	2,27	2,11	-2,27	1,98	Megavirus vitis;Vitis vinifera	Heat shock protein 70-like protein	VIT_00s0415g00030	F6HCT7_VITVI	11	20,50	73,27	15,14	3,27E+08
-876	2,22	1,59	-2,76	2,76	Vitis vinifera	S1 motif domain-containing protein	VIT_13s0067g01710	F6HC61_VITVI	6	19,20	38,80	7,26	1,28E+08
-876	3,43	2,18	-3,43	3,42	Vitis vinifera	Str_synth domain-containing protein	VIT_06s0009g00850	F6HAA8_VITVI	6	27,40	39,40	62,66	4,69E+08
-876	3,26	2,15	-3,30	3,30	Vitis vinifera	HATPase_c domain-containing protein	VIT_18s0001g14500	E0CQ80_VITVI	28	46,10	93,22	190,63	4,40E+09
-876	3,29	3,02	-3,29	2,26	Vitis vinifera	Cysteine synthase (EC 2.5.1.47)	VIT_04s0044g00250	F6I0J6_VITVI	10	40,40	40,50	60,33	1,02E+09

Cluster n°	Isabel (log2 fold-change)	Tenta (log2 fold-change)	Sciascinoso (log2 fold-change)	Aglianico (log2 fold-change)	Organism	Protein name	Gene name	UniProt name	Peptides	Sequence coverage [%]	Mol. weight [kDa]	Score	Intensity
-876	3,67	1,94	-3,68	3,68	Vitis vinifera	Glucose-6-phosphate 1-dehydrogenase (EC 1.1.1.49)	VIT_14s0171g00490	D7UBH2_VITVI	11	22,30	59,15	24,27	6,34E+08
-876	2,90	1,87	-2,90	1,78	Vitis vinifera	Uncharacterized protein	VIT_00s0455g00010	F6GZB0_VITVI	6	53,00	16,03	18,92	3,96E+08
-876	3,06	3,15	-3,15	2,73	Vitis vinifera	Dolichyl-diphosphooligosaccharide--protein glycosyltransferase subunit 2 (Ribophorin-2)	VIT_07s0005g03640	D7U318_VITVI	18	38,80	83,05	137,45	1,57E+09
-876	2,90	4,14	-4,30	4,30	Vitis vinifera	30S ribosomal protein S3, chloroplastic	VIT_09s0002g01460	D7TZU1_VITVI	10	51,70	26,18	56,44	4,15E+08
-876	4,32	3,10	-4,32	2,81	Vitis vinifera	GST N-terminal domain-containing protein	VIT_08s0040g03100	D7TQB0_VITVI	10	45,00	27,75	90,33	5,55E+08
-876	3,31	2,54	-3,31	2,99	Vitis vinifera	Dolichyl-diphosphooligosaccharide--protein glycosyltransferase 48 kDa subunit (Oligosaccharyl transferase 48 kDa subunit)	VIT_03s0063g02100	D7TPX8_VITVI	10	29,40	48,76	51,44	1,00E+09
-876	3,87	2,41	-3,87	2,16	Vitis vinifera	RRM domain-containing protein	VIT_16s0039g01860	D7T7N4_VITVI	7	36,10	29,33	60,26	6,25E+08
-876	3,93	1,98	-3,93	3,30	Vitis vinifera	ATP citrate synthase (EC 2.3.3.8)	VIT_05s0077g00950	D7SYK8_VITVI	24	50,50	65,99	323,31	9,50E+09
-876	4,26	3,93	-4,26	2,82	Vitis vinifera	Uncharacterized protein	VIT_17s0000g04910	D7SIN7_VITVI	11	49,10	33,89	145,15	2,16E+09
-876	3,19	2,25	-3,35	3,35	Vitis vinifera	Cytochrome b5 heme-binding domain-containing protein	VIT_16s0100g01280	A5C997_VITVI	5	47,90	23,52	42,40	1,11E+09
-876	2,37	3,76	-3,76	3,39	Vitis vinifera	Uncharacterized protein	VIT_09s0002g01040	F6HXV5_VITVI	7	20,70	61,10	35,95	6,84E+08
-876	2,76	2,85	-3,93	3,93	Vitis vinifera	PCI domain-containing protein	VIT_14s0030g01670	F6HTU0_VITVI	13	41,90	47,04	78,45	1,37E+09
-876	2,97	2,62	-2,97	2,42	Vitis vinifera	UDP-glucose 4-epimerase (EC 5.1.3.-)	VIT_02s0025g01580	A5BGY1_VITVI	9	41,70	38,03	38,96	6,23E+08
-876	4,12	3,12	-4,12	2,91	Megavirus vitis;Vitis vinifera	Ubiquitin	VITISV_036195	A5B102_VITVI	6	39,10	17,72	54,41	4,84E+09
-876	3,02	1,75	-3,02	2,75	Vitis vinifera	Uncharacterized protein	VIT_03s0063g02420	A5BFL3_VITVI	9	53,90	23,04	65,49	9,96E+07
-870	3,21	-4,86	-4,91	4,91	Vitis vinifera	Phosphoenolpyruvate carboxylase (EC 4.1.1.31)	VIT_19s0014g01390	F6H2N7_VITVI	45	58,90	109,82	109,55	1,79E+09
-870	5,94	-5,94	-5,39	4,31	Vitis vinifera	Uncharacterized protein	VITISV_034151	A5AIT3_VITVI	7	20,00	62,80	149,37	1,25E+09
-870	5,46	-3,42	-5,46	4,13	Vitis vinifera	Aminotran_1_2 domain-containing protein	VIT_09s0002g05250	D7U0Q3_VITVI	18	61,90	43,63	107,65	2,22E+09
-870	5,57	-4,76	-6,91	6,91	Vitis vinifera	Phosphoglycerate kinase (EC 2.7.2.3)	VIT_19s0085g00370	A5CAF6_VITVI	24	71,80	42,42	323,31	2,98E+10
-870	6,47	-6,47	-6,23	5,40	Vitis vinifera	Peptidyl-prolyl cis-trans isomerase (PPIase) (EC 5.2.1.8)	VIT_18s0001g14400	A5AKD8_VITVI	8	67,40	17,94	82,69	1,68E+10
-870	6,57	-4,51	-6,57	4,88	Vitis vinifera	Peptidyl-prolyl cis-trans isomerase (PPIase) (EC 5.2.1.8)	VIT_03s0038g01930	A5BQN6_VITVI	8	48,80	18,31	138,36	5,05E+09
-870	2,35	-2,35	-1,58	1,46	Vitis vinifera	Dihydrolipoamide acetyltransferase component of pyruvate dehydrogenase complex (EC 2.3.1.-)	VIT_13s0074g00150	F6I5U2_VITVI	8	22,00	68,35	27,12	5,22E+08
-870	2,20	-1,71	-2,97	2,97	Vitis vinifera	HATPase_c domain-containing protein	VIT_19s0015g01090	F6I581_VITVI	33	49,40	80,85	323,31	1,21E+09
-870	1,98	-2,39	-1,39	2,39	Vitis vinifera	Uncharacterized protein	VIT_10s0003g04180	F6HLU8_VITVI	18	27,30	81,69	79,66	3,63E+09
-870	2,50	-2,50	-1,47	1,56	Vitis vinifera	Beta-glucosidase (EC 3.2.1.21)	BG1	D3YJ59_VITVI	17	51,90	55,22	76,18	2,30E+09
-870	1,35	-1,54	-1,90	1,90	Vitis vinifera	Catalase (EC 1.11.1.6)	VIT_18s0122g01320	D7UD99_VITVI	14	43,90	56,97	49,86	1,07E+09
-870	3,23	-3,23	-1,84	1,82	Vitis vinifera	Formate dehydrogenase, mitochondrial (FDH) (EC 1.17.1.9) (NAD-dependent formate dehydrogenase)	VIT_14s0066g01320	D7TWQ4_VITVI	21	73,90	42,05	216,23	8,45E+09
-870	2,27	-2,43	-3,46	3,46	Vitis vinifera	Reticulon-like protein	VIT_02s0025g00640	D7TV84_VITVI	4	22,20	28,10	18,11	8,59E+08
-870	2,38	-4,15	-2,17	4,15	Vitis vinifera	Pyruvate kinase (EC 2.7.1.40)	VIT_08s0007g04170	D7TIZ5_VITVI	18	46,50	55,96	278,87	3,45E+09
-870	2,48	-1,92	-2,48	1,89	Vitis vinifera	Succinate--CoA ligase [ADP-forming] subunit alpha, mitochondrial (EC 6.2.1.5) (Succinyl-CoA synthetase subunit alpha) (SCS-alpha)	VIT_10s0042g00950	D7TEX3_VITVI	4	20,40	34,33	2,86	1,06E+08
-870	1,68	-1,32	-2,13	2,13	Vitis vinifera	Uncharacterized protein	VIT_04s0008g06320	D7SV17_VITVI	7	34,70	32,72	18,59	4,11E+08
-870	2,99	-2,99	-1,55	1,64	Vitis vinifera	Uncharacterized protein	VIT_12s0028g03380	F6H5F0_VITVI	13	40,20	41,10	24,84	1,71E+09
-870	2,27	-2,72	-2,77	2,77	Vitis vinifera	Uncharacterized protein	VIT_08s0040g01190	F6HR64_VITVI	7	29,70	30,38	41,01	5,42E+08
-870	3,03	-3,03	-2,06	2,25	Vitis vinifera	Uncharacterized protein	VIT_03s0097g00590	D7TY71_VITVI	5	29,80	34,32	11,85	6,57E+08
-870	4,62	-2,90	-4,62	3,15	Vitis vinifera	ADF-H domain-containing protein	VIT_07s0005g03310	F6HZJ8_VITVI	5	54,50	16,55	48,00	5,20E+08
-870	4,61	-4,12	-4,61	3,69	Vitis hybrid cultivar;Vitis vinifera	Pectinesterase (EC 3.1.1.11)	TamPME2	A9CSJ7_9ROSI	9	23,90	61,65	133,33	2,48E+09
-870	5,16	-3,26	-5,16	3,84	Vitis vinifera	Aminotran_1_2 domain-containing protein	VIT_09s0002g00340	F6HXL0_VITVI	13	35,60	57,85	140,05	2,12E+09
-870	4,18	-4,18	-3,75	2,16	Vitis vinifera	Putative oxalyl-CoA decarboxylase	Oxc	Q6XGX9_VITVI	18	38,10	60,99	71,09	2,12E+09
-870	4,10	-4,10	-3,94	2,33	Vitis vinifera	Uncharacterized protein	VIT_01s0026g01650	F6HPF2_VITVI	7	29,70	29,43	95,17	2,98E+09
-870	4,59	-3,41	-4,59	3,25	Vitis vinifera	Aspartic proteinase (Fragment)	*N/A*	B5KFB6_VITVI	15	39,10	57,64	106,91	2,31E+09
-870	4,12	-3,49	-4,12	2,21	Vitis vinifera	14_3_3 domain-containing protein	VIT_18s0001g05720	E0CRL1_VITVI	13	60,30	28,74	113,07	1,43E+09
-870	4,03	-3,10	-4,03	2,36	Vitis vinifera	Succinate dehydrogenase [ubiquinone] flavoprotein subunit, mitochondrial (EC 1.3.5.1)	VIT_04s0023g01650	D7SPF1_VITVI	15	35,80	73,66	202,99	2,19E+09
-870	3,93	-3,93	-2,97	2,07	Vitis vinifera	Uncharacterized protein	VITISV_006460	A5ANH8_VITVI	20	51,30	55,63	323,31	5,92E+09
-870	4,92	-3,64	-4,92	2,84	Vitis vinifera	Uncharacterized protein	VIT_07s0129g00100	F6HST0_VITVI	12	50,40	29,29	73,72	2,82E+09

Cluster n°	Isabel (log2 fold-change)	Tenta (log2 fold-change)	Sciascinoso (log2 fold-change)	Aglianico (log2 fold-change)	Organism	Protein name	Gene name	UniProt name	Peptides	Sequence coverage [%]	Mol. weight [kDa]	Score	Intensity
-875	1,69	-2,77	1,42	2,77	Vitis vinifera	Aspartate-semialdehyde dehydrogenase (EC 1.2.1.11)	VIT_01s0011g05860	F6HFC9_VITVI	3	14,00	41,33	15,27	3,06E+08
-875	2,57	-2,57	1,78	1,91	Vitis vinifera	Ubiquitin-fold modifier 1	VIT_18s0001g03580	E0CRB0_VITVI	2	44,30	10,13	4,38	1,36E+08
-875	2,45	-2,45	1,44	2,27	Vitis vinifera	Pyrophosphate--fructose 6-phosphate 1-phosphotransferase subunit beta (PFP) (EC 2.7.1.90) (6-phosphofructokinase, pyrophosphate dependent) (PPi-PFK) (Pyrophosphate-dependent 6-phosphofructose-1-kinase)	PFP-BETA	D7TR81_VITVI	13	34,20	61,51	89,75	9,43E+08
-875	2,82	-2,82	2,12	1,37	Vitis vinifera	Beta-galactosidase (EC 3.2.1.23)	VIT_06s0004g03020	D7SKW9_VITVI	3	6,60	92,18	2,39	1,40E+08
-875	2,18	-2,18	0,00	2,18	Vitis vinifera	Uncharacterized protein	VIT_11s0016g01070	A5CBB3_VITVI	6	37,80	39,86	41,24	5,27E+08
-875	1,45	-1,49	1,49	1,38	Vitis vinifera	Fructose-bisphosphate aldolase (EC 4.1.2.13)	VIT_19s0015g01720	D7UAT6_VITVI	21	71,80	38,63	323,31	3,59E+10
-875	2,65	-2,65	1,77	2,25	Vitis vinifera	NADH:ubiquinone reductase (non-electrogenic) (EC 1.6.5.9);	VITISV_027063	A5BWF5_VITVI	7	18,20	64,98	19,99	4,40E+08
-875	2,65	-2,65	1,77	2,25	Vitis vinifera	Pyr_redox_2 domain-containing protein (Fragment)	VIT_00s2376g00010	F6HS19_VITVI	7	18,20	64,98	19,99	4,40E+08
-875	2,56	-4,03	4,03	2,16	Vitis vinifera	Glutamate decarboxylase (EC 4.1.1.15)	VIT_01s0011g06610	F6I1W0_VITVI	22	63,60	57,13	323,31	3,85E+09
-875	4,35	-4,35	4,08	3,10	Vitis vinifera	ATP citrate synthase (EC 2.3.3.8)	VIT_14s0060g00550	D7UA89_VITVI	12	43,30	46,43	67,00	9,58E+08
-875	3,27	-3,27	1,84	3,19	Vitis vinifera	Aconitate hydratase (Aconitase) (EC 4.2.1.3)	VIT_12s0059g02150	D7TEL2_VITVI	32	46,90	110,06	323,31	1,15E+10
-875	3,55	-3,55	2,60	3,53	Vitis vinifera	Pyruvate kinase (EC 2.7.1.40)	40N03_150	C5DB68_VITVI	15	45,50	55,10	133,84	1,23E+09
-875	2,42	-3,91	3,91	3,89	Vitis vinifera	Plastocyanin	VIT_18s0001g00760	E0CQV6_VITVI	3	38,70	17,01	95,97	2,77E+09
-875	3,90	-3,96	3,46	3,96	Vitis vinifera	Uncharacterized protein	VIT_17s0000g00400	F6GT37_VITVI	14	51,20	32,47	69,31	1,19E+09
-875	4,51	-4,99	4,15	4,99	Vitis vinifera	LOS4 (Fragment)	*N/A*	A0A097PLZ9_VITVI	10	34,50	57,06	35,74	7,49E+08
-875	5,60	-5,60	5,38	5,45	Vitis vinifera	Sucrose-phosphate synthase (EC 2.4.1.14)	VIT_04s0008g05730	F6H3I4_VITVI	33	38,40	118,13	311,70	5,38E+09
-875	5,34	-5,34	3,58	3,82	Vitis vinifera	Succinate dehydrogenase [ubiquinone] iron-sulfur subunit, mitochondrial (EC 1.3.5.1)	VIT_14s0219g00150	F6GX63_VITVI	7	22,20	45,32	36,16	8,53E+08
-875	7,11	-7,11	6,53	5,49	Vitis quinquangularis;Vitis vinifera	Grip22-like protein;Ripening-related protein grip22	grip22	GRI22_VITVI	4	39,50	22,80	102,37	7,88E+09
-875	5,83	-6,20	5,98	6,20	Vitis riparia (Frost grape) (Vitis vulpina);Vitis vinifera	26S proteasome regulatory ATPase subunit S10b	Rev136-3;	Q9M606_VITRI	11	43,60	44,66	92,80	8,28E+08
-882	-7,76	8,13	7,84	-8,13	Vitis vinifera	Aldedh domain-containing protein	VIT_11s0037g00070	D7U1A1_VITVI	9	22,40	53,17	40,83	4,56E+08
-882	-7,85	6,90	7,85	-5,96	Vitis vinifera	Protein kinase domain-containing protein	VITISV_024931	A5CBA7_VITVI	13	19,00	83,32	262,29	5,68E+09
-882	-7,40	7,40	5,01	-7,19	Vitis vinifera	Putative ripening-related protein	grip68	Q9M4H6_VITVI	7	89,10	15,39	264,97	4,55E+09
-882	-6,86	4,70	6,86	-5,83	Vitis vinifera	Annexin	VIT_18s0001g05530	A5BTZ8_VITVI	18	55,30	35,19	241,28	4,05E+09
-882	-5,00	4,26	6,31	-6,31	Vitis vinifera	Annexin	VITISV_030870	A5B479_VITVI	17	51,60	35,62	137,22	2,58E+09
-882	-6,54	6,54	4,46	-6,37	Vitis vinifera	Phosphomannomutase (EC 5.4.2.8)	VIT_15s0046g03520	D7UC38_VITVI	7	41,40	28,11	32,64	6,04E+08
-882	-2,44	1,47	3,03	-3,03	Vitis vinifera	FAM86 domain-containing protein	VIT_14s0128g00100	F6HYX7_VITVI	6	9,20	112,23	24,27	2,53E+08
-882	-2,65	1,39	2,65	-1,63	Vitis vinifera	GST N-terminal domain-containing protein	VITISV_021571	A5CAR3_VITVI	4	20,10	32,53	4,88	1,12E+08
-882	-2,65	2,99	2,61	-2,99	Vitis vinifera	SHSP domain-containing protein	VITISV_044042	A5BAN4_VITVI	7	20,40	53,16	104,82	2,34E+08
-882	-3,14	3,14	2,52	-1,97	Vitis vinifera	Fructose-bisphosphate aldolase (EC 4.1.2.13)	VIT_04s0023g03010	F6GWQ0_VITVI	6	19,90	42,80	62,45	1,56E+08
-882	-3,33	3,26	3,33	-1,58	Vitis vinifera	Uncharacterized protein	VIT_03s0038g02510	D7U543_VITVI	11	36,80	42,28	17,82	5,91E+08
-882	-3,77	3,77	2,33	-2,12	Vitis vinifera	RanBD1 domain-containing protein	VIT_04s0008g01410	D7STS9_VITVI	3	28,60	24,58	113,43	4,80E+08
-882	-2,74	1,83	2,74	-1,44	Vitis vinifera	Cytochrome b561 and DOMON domain-containing protein	VIT_05s0062g00850	D7U9K7_VITVI	3	12,60	43,95	6,13	2,51E+08
-882	-1,95	1,68	2,74	-2,74	Vitis vinifera	NADH-u_ox-rdase domain-containing protein	VIT_19s0090g01760	D7T881_VITVI	2	25,00	10,91	9,12	1,51E+08
-882	-2,54	1,59	2,54	-1,31	Vitis vinifera	Adenosylhomocysteinase (EC 3.3.1.1) (S-adenosyl-L-homocysteine hydrolase) (AdoHcyase)	VIT_05s0029g00330	A5C5K3_VITVI	24	64,10	53,18	321,84	3,37E+09
-882	-3,20	3,62	2,57	-3,62	Vitis vinifera	Putative ripening-related protein	grip31	Q9M4H7_VITVI	15	93,60	18,42	323,31	5,37E+10
-882	-4,80	2,84	4,80	-4,06	Vitis vinifera	Chlorophyll a-b binding protein, chloroplastic	Lhca3	Q6Y0E5_VITVI	10	60,90	29,53	40,35	7,72E+08
-882	-4,56	3,14	4,56	-4,05	Vitis rotundifolia (Muscadine grape);Vitis vinifera	Glucan endo-1,3-beta-D-glucosidase (EC 3.2.1.39)	Glub	Q9ZTK2_VITVI	15	73,00	37,50	323,31	7,71E+08
-882	-4,87	3,99	4,87	-4,69	Vitis vinifera	SHSP domain-containing protein	VIT_12s0035g01910	F6HJZ4_VITVI	6	30,00	28,39	19,46	4,53E+08
-882	-5,06	4,59	5,06	-4,82	Vitis vinifera	DRT100-like protein	VIT_13s0064g01260	F6HBA8_VITVI	7	19,40	37,95	12,63	2,93E+08
-882	-3,59	3,70	4,70	-4,70	Vitis rupestris;Vitis vinifera	Metacaspase-5	VIT_19s0085g01030	F6H9S5_VITVI	9	34,90	46,25	74,39	1,00E+09
-882	-4,67	3,53	4,67	-2,93	Vitis vinifera	Tr-type G domain-containing protein	VIT_19s0027g00760	F6H4T7_VITVI	35	50,90	95,24	323,31	8,45E+09

Cluster n°	Isabel (log2 fold-change)	Tenta (log2 fold-change)	Sciascinoso (log2 fold-change)	Aglianico (log2 fold-change)	Organism	Protein name	Gene name	UniProt name	Peptides	Sequence coverage [%]	Mol. weight [kDa]	Score	Intensity
-882	-3,85	3,85	3,15	-3,82	Vitis vinifera	Uncharacterized protein	VIT_08s0058g00860	F6GXZ3_VITVI	6	27,80	23,72	10,59	4,81E+08
-882	-4,69	4,69	4,02	-2,98	Vitis vinifera	Uncharacterized protein	VIT_13s0019g03350	D7TMH3_VITVI	7	21,10	42,53	28,27	3,61E+08
-882	-3,29	3,98	3,64	-3,98	Vitis vinifera	Adenosine kinase (EC 2.7.1.20);PfkB domain-containing protein	VIT_13s0019g04470	D7TM77_VITVI	15	63,60	36,79	266,21	5,47E+09
-882	-4,74	3,91	5,11	-5,11	Vitis riparia (Frost grape) (Vitis vulpina)	Glucan endo-1,3-beta-D-glucosidase (EC 3.2.1.39)	*N/A*	B2ZP01_VITRI	12	57,70	37,40	128,26	3,48E+08
-882	-3,89	3,80	3,89	-2,19	Vitis vinifera	Phosphoglycerate kinase (EC 2.7.2.3)	VIT_19s0085g00380	A5CAF8_VITVI	22	63,20	50,08	133,02	3,00E+09
-882	-3,39	3,61	2,88	-3,61	Vitis vinifera	Usp domain-containing protein	VIT_08s0007g01430	A5C9Q0_VITVI	9	70,70	18,24	69,48	7,80E+08
-882	-5,28	5,28	3,98	-4,52	Vitis vinifera	4-hydroxy-4-methyl-2-oxoglutarate aldolase (HMG aldolase) (EC 4.1.1.112) (EC 4.1.3.17) (Oxaloacetate decarboxylase)	VIT_04s0023g00590	F6GX20_VITVI	7	50,60	17,77	107,98	3,32E+09
-882	-4,69	4,69	4,15	-3,70	Vitis vinifera	ATPase_AAA_core domain-containing protein	VIT_06s0004g05180	D7SKB2_VITVI	9	23,70	51,88	99,38	3,57E+08
-882	-4,33	3,58	4,33	-2,59	Vitis vinifera	Uncharacterized protein	VIT_08s0007g02620	D7TH43_VITVI	6	55,60	16,19	18,20	7,32E+08
-882	-2,58	3,68	3,63	-3,68	Vitis vinifera	LRRNT_2 domain-containing protein	VITISV_017226	A5BFQ1_VITVI	4	13,20	51,04	11,83	1,10E+08
-882	-4,48	4,27	4,48	-3,40	Vitis quinquangularis;Vitis vinifera	Uncharacterized protein	VIT_04s0008g05010	F6H3T7_VITVI	10	39,90	40,33	323,31	3,96E+10
-882	-4,20	3,76	4,20	-3,36	Vitis vinifera	Fumarylacetoacetase (EC 3.7.1.2) (Fumarylacetoacetate hydrolase)	VIT_00s0187g00300	F6HRM6_VITVI	8	28,40	45,54	44,92	1,30E+09
-788	-5,73	5,90	-5,74	-5,90	Vitis rupestris x Vitis vinifera	Polygalacturonase-inhibiting protein	*N/A*	*N/A*	8	28,50	37,06	171,84	4,04E+09
-788	-4,47	4,47	-3,67	-4,08	Vitis vinifera	HP domain-containing protein	VITISV_028790	A5BX87_VITVI	6	14,40	57,66	22,04	2,98E+08
-788	-2,52	3,49	-3,35	-3,49	Vitis vinifera	AAA domain-containing protein	VITISV_041357	A5BTI7_VITVI	7	18,70	55,03	17,98	2,08E+08
-788	-2,52	3,80	-3,14	-3,80	Vitis vinifera	Uncharacterized protein	VIT_00s0245g00010	F6I748_VITVI	2	11,50	42,27	3,72	2,62E+08
-788	-2,33	4,30	-3,04	-4,30	Vitis vinifera	14_3_3 domain-containing protein;14-3-3 protein	VIT_18s0001g06330	F6H0X3_VITVI	14	63,80	29,34	225,28	2,59E+09
-788	-4,95	4,95	-3,46	-4,36	Vitis vinifera	Peroxidase (EC 1.11.1.7)	VIT_18s0072g00160	F6GY60_VITVI	7	31,60	36,49	56,48	1,10E+09
-788	-4,01	4,50	-4,47	-4,50	Grapevine virus A	Coat protein	*N/A*	*N/A*	7	53,80	17,30	79,02	1,21E+08
-788	-4,00	4,00	-3,35	-3,29	Vitis vinifera	Uncharacterized protein	VIT_18s0001g02740	E0CR63_VITVI	8	32,20	28,58	27,91	2,10E+09
-788	-2,48	3,55	-3,55	-3,49	Vitis vinifera	Alpha-mannosidase (EC 3.2.1.-)	VITISV_032907	A5AHH3_VITVI	14	16,30	112,43	53,06	8,68E+08
-788	-3,34	3,75	-3,26	-3,75	Vitis vinifera	Fatty acid hydroperoxide lyase (EC 4.1.2.-)	HPLA	D5FV11_VITVI	21	56,70	54,75	16,56	3,84E+08
-788	-4,52	5,75	-5,17	-5,75	Vitis vinifera	Bet_v_1 domain-containing protein	VITISV_025730	A5C111_VITVI	3	30,80	14,89	74,48	2,50E+08
-788	-5,41	5,43	-4,85	-5,43	Vitis hybrid cultivar;Vitis vinifera	Lipoxygenase (EC 1.13.11.-)	VITISV_027685	A5ANS7_VITVI;	33	54,40	101,67	323,31	6,11E+09
-852	0,00	1,63	-1,63	-1,48	Vitis vinifera	Pyruvate kinase (EC 2.7.1.40)	VIT_08s0007g05490	F6HLQ8_VITVI	5	14,60	53,63	5,68	7,47E+07
-852	0,00	1,67	-1,67	-1,39	Vitis vinifera	Aha1_N domain-containing protein	VIT_08s0007g06710	F6HLH5_VITVI	4	17,90	38,48	5,41	1,42E+08
-852	0,00	2,37	-2,33	-2,37	Vitis vinifera	Acetohydroxy-acid synthase small subunit	VITISV_043824	A5BKL2_VITVI	6	16,80	52,95	13,68	3,19E+08
-852	0,00	2,44	-2,44	-1,90	Vitis vinifera	Chloroplast ELIP early light-induced protein	*N/A*	D6QST4_VITVI	5	41,10	21,07	41,92	2,47E+08
-852	0,00	1,80	-1,73	-1,80	Vitis vinifera	Uncharacterized protein	VIT_01s0011g03450	F6HEX2_VITVI	2	2,30	100,95	5,87	6,38E+07
-852	0,00	1,90	-1,69	-1,90	Vitis vinifera	Uncharacterized protein	VIT_13s0019g02090	D7TLP9_VITVI	2	13,10	23,43	7,97	6,68E+07
-852	0,00	2,52	-2,21	-2,52	Vitis vinifera	Fructose-bisphosphate aldolase (EC 4.1.2.13)	VIT_03s0038g00670	A5BDH7_VITVI	5	17,60	42,95	12,04	6,30E+07
-852	0,00	2,20	-2,14	-2,20	Vitis vinifera	HATPase_c domain-containing protein;	VITISV_021022	A5AEP7_VITVI	2	4,80	90,46	3,79	6,78E+07
-852	0,00	2,28	-2,28	-1,87	Vitis labrusca (Concord grape);Vitis vinifera	Aminotran_1_2 domain-containing protein	VIT_00s0225g00130	D7SYR7_VITVI	5	17,20	52,72	21,05	9,80E+07
-852	0,00	1,53	-1,51	-1,53	Vitis vinifera	Purple acid phosphatase (EC 3.1.3.2)	VIT_11s0118g00240	F6HCG3_VITVI	3	8,40	53,20	5,16	1,09E+08
-852	-2,14	2,17	0,00	-2,17	Vitis vinifera	Uncharacterized protein	VIT_18s0164g00030	F6HVW3_VITVI	4	19,30	26,39	9,75	2,15E+08
-852	0,00	1,50	0,00	-1,50	Vitis vinifera	Eukaryotic translation initiation factor 3 subunit E (eIF3e) (Eukaryotic translation initiation factor 3 subunit 6)	VIT_00s0252g00070	F6HS75_VITVI	9	28,10	51,13	30,28	6,38E+08
-852	0,00	1,62	0,00	-1,62	Vitis vinifera	Usp domain-containing protein	VIT_01s0011g03680	F6HEW0_VITVI	3	35,30	18,22	11,40	1,59E+08
-852	-1,88	1,88	0,00	-1,50	Vitis vinifera	Usp domain-containing protein	VIT_12s0134g00430	F6GXR1_VITVI	4	24,50	27,63	36,95	5,06E+08
-852	0,00	1,75	0,00	-1,75	Vitis vinifera	Uncharacterized protein	VIT_06s0061g00270	F6GWA8_VITVI	17	42,50	61,98	140,94	1,69E+09
-852	0,00	1,54	0,00	-1,54	Vitis vinifera	EF1_GNE domain-containing protein	VIT_17s0000g01540	D7SGX1_VITVI	9	44,10	26,73	25,36	2,31E+09
-852	-1,58	1,76	0,00	-1,76	Vitis vinifera	Glycosyltransferase (EC 2.4.1.-)	VIT_03s0063g00050	A5BR79_VITVI	4	16,20	52,75	8,26	2,46E+08
-852	-2,02	2,02	0,00	-1,31	Vitis vinifera	3-oxoacyl-[acyl-carrier-protein] synthase	VIT_10s0003g03070	F6HMJ0_VITVI	4	12,00	46,93	17,68	2,75E+08
-852	0,00	1,69	0,00	-1,69	Vitis vinifera	Uncharacterized protein	VIT_04s0023g03520	F6GWM3_VITVI	2	15,80	23,42	15,23	1,90E+08

Cluster n°	Isabel (log2 fold-change)	Tenta (log2 fold-change)	Sciascinoso (log2 fold-change)	Aglianico (log2 fold-change)	Organism	Protein name	Gene name	UniProt name	Peptides	Sequence coverage [%]	Mol. weight [kDa]	Score	Intensity
-852	0,00	1,61	0,00	-1,61	Vitis vinifera	SHSP domain-containing protein	VIT_01s0010g02290	D7TAL0_VITVI	2	11,50	20,74	2,73	1,26E+08
-852	0,00	1,92	0,00	-1,92	Vitis vinifera	Hydrolase_4 domain-containing protein	VIT_13s0019g04260	F6HNG8_VITVI	3	18,20	36,15	14,96	1,26E+08
-852	0,00	1,62	0,00	-1,62	Vitis vinifera	D-fructose-1,6-bisphosphate 1-phosphohydrolase (EC 3.1.3.11)	VIT_08s0007g01570	F6HKY4_VITVI	2	6,10	44,67	5,61	8,47E+07
-852	-1,91	3,10	-1,60	-3,10	Vitis vinifera	Chloroplast chaperonin 21	cpn21	Q6B4V4_VITVI	5	32,30	26,40	18,28	4,82E+08
-852	-2,34	2,34	-2,26	-2,00	Grapevine virus A;Grapevine virus A (isolate Is 151) (GVA)	Capsid protein (CP) (Coat protein)	*N/A*	*N/A*	6	39,90	21,65	5,56	2,10E+08
-852	-2,00	2,80	-1,78	-2,80	Vitis vinifera	Uncharacterized protein	VIT_07s0129g00490	F6HSV1_VITVI	3	4,80	81,14	9,38	1,14E+08
-852	-2,62	2,89	-2,19	-2,89	Vitis vinifera	Abhydrolase_3 domain-containing protein	VIT_03s0063g00780	F6HQD3_VITVI	2	11,30	33,42	12,59	1,18E+08
-852	-2,43	3,23	-1,88	-3,23	Vitis vinifera	Ribonuclease	VIT_00s0283g00020	D7TYP5_VITVI	16	25,00	109,44	46,81	7,18E+08
-852	-2,19	3,16	-3,10	-3,16	Vitis hybrid cultivar;Vitis vinifera	Chlorophyll a-b binding protein, chloroplastic	TamCAB	A9CSG0_9ROSI	7	43,80	28,28	3,05	1,85E+08
-852	-2,80	2,80	0,00	-2,72	Vitis vinifera	HABP4_PAI-RBP1 domain-containing protein	VIT_00s0620g00020	D7TGB6_VITVI	5	22,00	39,16	29,35	4,60E+08
-852	-1,73	3,30	-3,30	-2,84	Vitis vinifera	40S ribosomal protein S25	VIT_03s0038g04560	A5BRH6_VITVI	3	33,60	12,20	29,44	1,78E+08
-852	-2,44	3,50	-3,50	-2,91	Vitis vinifera	PMR5N domain-containing protein	VITISV_007322	A5AM41_VITVI	11	50,70	41,45	16,49	3,51E+08
-852	-1,83	1,89	-1,89	-1,75	Vitis vinifera	XendoU domain-containing protein	VIT_02s0012g00180	D7TTJ6_VITVI	2	7,90	51,13	4,63	7,90E+07
-852	-2,51	2,52	-2,52	-1,96	Vitis heyneana;Vitis vinifera	Aquaporin PIP23	PIP2;3	A3FA68_VITVI	3	15,30	30,69	28,67	2,40E+08
-852	-3,76	3,76	-2,74	-2,24	Vitis vinifera	ATP synthase subunit alpha	atpA	A5BSB1_VITVI	14	37,70	55,44	158,33	4,30E+09
-852	-1,67	2,10	-2,10	-1,80	Vitis vinifera	Shikimate dehydrogenase (EC 1.1.1.25)	VIT_05s0020g02030	F6HDI5_VITVI	2	10,40	24,42	3,28	5,82E+07
-614	1,97	2,34	-2,26	-2,34	Vitis vinifera	GrpE protein homolog	VIT_08s0056g00810	F6HMV2_VITVI	4	16,60	37,19	23,47	2,49E+08
-614	3,12	1,95	-1,69	-3,12	Vitis vinifera	Phosphoglucomutase (alpha-D-glucose-1,6-bisphosphate-dependent) (EC 5.4.2.2)	VIT_01s0011g05370	F6HFF7_VITVI	25	54,40	67,93	204,01	8,49E+09
-614	1,81	2,44	-2,44	-1,61	Vitis vinifera	Coatomer subunit alpha	VIT_18s0001g13430	F6GZQ1_VITVI	3	3,60	137,00	12,75	1,00E+08
-614	2,10	1,66	-1,64	-2,10	Vitis vinifera	Protein disulfide-isomerase (EC 5.3.4.1)	VIT_06s0004g02890	F6GU04_VITVI	9	17,50	64,29	14,01	4,71E+08
-614	1,42	1,64	-1,60	-1,64	Vitis vinifera	Thioredoxin domain-containing protein	VIT_00s0238g00110	F6I7C5_VITVI	2	29,60	7,86	11,24	1,11E+08
-614	1,45	1,98	-1,44	-1,98	Vitis vinifera	Glucose-6-phosphate isomerase (EC 5.3.1.9)	VIT_18s0001g12370	E0CQ39_VITVI	11	33,10	62,65	24,22	9,01E+08
-614	2,77	2,41	-2,77	-2,63	Vitis vinifera	Cytochrome c oxidase subunit Vb	COX5b.1	K4HRN3_VITVI	7	42,60	19,53	47,72	9,74E+08
-614	1,43	3,02	-2,55	-3,02	Vitis vinifera	Plasma membrane ATPase (EC 7.1.2.1)	VIT_17s0000g05540	D7SIH5_VITVI	17	21,30	105,31	30,59	2,58E+08
-614	2,24	1,33	-2,24	-1,65	Vitis vinifera	Uncharacterized protein	VIT_17s0000g01740	D7SGV2_VITVI	5	14,60	53,51	7,16	2,35E+08
-614	2,86	2,43	-2,61	-2,86	Vitis vinifera	Thioredoxin domain-containing protein	VIT_00s0532g00030	D7TRU8_VITVI	4	23,00	14,22	7,27	2,35E+08
-614	3,35	1,88	-3,35	-2,55	Vitis vinifera	Protein disulfide-isomerase (EC 5.3.4.1)	VITISV_038267	A5C7J5_VITVI	14	47,30	39,27	307,49	5,63E+09
-614	1,36	1,77	-1,58	-1,77	Vitis vinifera	Uncharacterized protein	VIT_01s0011g00830	F6HF70_VITVI	3	33,60	14,98	11,92	1,83E+08
-614	2,95	1,69	-2,95	-2,18	Vitis vinifera	NADH-cytochrome b5 reductase (EC 1.6.2.2)	VIT_11s0016g04970	D7TBY5_VITVI	10	41,10	35,85	108,71	2,06E+09
-614	2,18	1,78	-2,08	-2,18	Vitis vinifera	Uncharacterized protein	VITISV_039575	A5B9G1_VITVI	3	3,00	124,13	10,66	1,34E+08
-614	1,87	2,13	-2,13	-1,50	Vitis vinifera	Isocitrate dehydrogenase [NADP] (EC 1.1.1.42)	VITISV_015982	A5B8K4_VITVI	3	8,40	54,94	24,24	5,15E+08
-614	2,75	2,36	-2,75	-1,52	Vitis vinifera	LEA_2 domain-containing protein	VIT_16s0039g00340	F6HE80_VITVI	4	20,80	23,08	7,23	5,92E+08
-614	1,47	2,48	-2,21	-2,48	Vitis vinifera	Xyloglucan endotransglucosylase/hydrolase (EC 2.4.1.207)	VITISV_037683	A5AQ11_VITVI	3	12,50	33,18	21,48	1,36E+08
-840	0,00	0,00	-1,80	1,80	Vitis vinifera	WD_REPEATS_REGION domain-containing protein	VIT_15s0048g00960	A5B5Z6_VITVI	5	13,70	47,69	8,89	1,65E+08
-840	1,60	0,00	-1,60	1,37	Vitis vinifera	Iso_dh domain-containing protein	VIT_03s0038g03120	F6I0W2_VITVI	7	31,50	39,98	38,32	5,94E+08
-840	0,00	0,00	-2,20	2,20	Vitis vinifera	Sarcosine oxidase (EC 1.5.3.1)	VIT_04s0069g00860	F6H9I1_VITVI	2	6,30	44,98	5,40	8,61E+07
-840	1,43	0,00	-2,02	2,02	Vitis vinifera	NAD(P)-bd_dom domain-containing protein	VIT_04s0008g07400	F6H3M3_VITVI	6	28,70	32,09	26,79	6,62E+08
-840	2,24	0,00	-2,24	1,56	Vitis vinifera	ADP-ribosyl cyclase/cyclic ADP-ribose hydrolase (EC 3.2.2.6)	VITISV_036339	A5BLG1_VITVI	8	8,80	115,66	7,45	1,26E+08
-840	2,24	0,00	-2,24	1,56	Vitis vinifera	Sucrose-phosphate synthase (EC 2.4.1.14)	VIT_18s0089g00410	F6GW11_VITVI	8	8,80	115,66	7,45	1,26E+08
-840	2,37	0,00	-2,37	2,13	Vitis vinifera	Uncharacterized protein	VIT_16s0050g00390	E0CV25_VITVI	9	29,80	56,17	89,02	4,85E+08
-840	1,61	0,00	-1,76	1,76	Vitis vinifera	Obg-like ATPase 1	VIT_10s0003g05020	D7TKK5_VITVI	13	40,10	44,42	27,36	7,67E+08
-840	1,61	0,00	-1,76	1,76	Vitis vinifera	OBG-type G domain-containing protein	VITISV_035841	A5B4W7_VITVI	13	40,10	44,42	27,36	7,67E+08
-840	1,47	0,00	-1,47	1,30	Vitis vinifera	Protein disulfide-isomerase (EC 5.3.4.1)	VIT_12s0059g01560	D7TEF8_VITVI	5	16,90	46,61	16,76	6,80E+08
-840	1,47	0,00	-1,88	1,88	Vitis vinifera	SMP-LTD domain-containing protein	VITISV_029347	A5ANF0_VITVI	2	4,80	64,11	3,13	1,12E+08
-840	0,00	0,00	-1,35	1,35	Vitis vinifera	Uncharacterized protein	VIT_05s0077g01940	D7SYA9_VITVI	4	5,10	91,05	8,83	1,48E+08
-840	0,00	0,00	-1,73	1,73	Vitis vinifera	Uncharacterized protein	VIT_04s0008g02720	D7SU41_VITVI	5	12,50	51,35	14,37	8,69E+07

Cluster n°	Isabel (log2 fold-change)	Tenta (log2 fold-change)	Sciascinoso (log2 fold-change)	Aglianico (log2 fold-change)	Organism	Protein name	Gene name	UniProt name	Peptides	Sequence coverage [%]	Mol. weight [kDa]	Score	Intensity
-840	0,00	0,00	-1,68	1,68	Vitis vinifera	Glutaredoxin domain-containing protein	VIT_11s0052g00500	D7SQE1_VITVI	3	36,00	14,70	69,08	4,06E+08
-840	0,00	0,00	-1,50	1,50	Vitis vinifera	Vesicle-fusing ATPase (EC 3.6.4.6)	VIT_19s0014g02750	E0CSK6_VITVI	13	23,70	82,98	40,44	6,29E+08
-840	0,00	0,00	-1,66	1,66	Vitis vinifera	Eukaryotic translation initiation factor 5A (eIF-5A)	VIT_11s0016g00020	A5BZP4_VITVI	6	43,10	17,47	55,86	4,76E+08
-840	0,00	1,51	-1,94	1,94	Vitis vinifera	Mitochondrial pyruvate carrier	VITISV_018795	A5BCM7_VITVI	4	14,00	45,83	9,73	1,75E+08
-840	2,36	0,00	-2,36	1,68	Vitis vinifera	DLH domain-containing protein	VIT_07s0104g00440	D7TPB8_VITVI	11	50,60	26,10	30,60	1,54E+09
-840	0,00	0,00	-1,60	1,60	Vitis vinifera	Uncharacterized protein	VIT_05s0094g01520	D7T2N7_VITVI	14	64,00	34,94	86,91	5,21E+09
-840	0,00	0,00	-1,35	1,35	Vitis vinifera	Proteasome subunit alpha type	VIT_09s0002g00980	A5ALB2_VITVI	11	60,20	27,33	216,40	3,10E+09
-840	1,63	0,00	-2,13	2,13	Vitis vinifera	Aldo_ket_red domain-containing protein	VITISV_016804	A5B3N3_VITVI	16	53,10	37,49	148,08	8,95E+07
-840	1,77	1,73	-2,07	2,07	Vitis vinifera	ABC transporter domain-containing protein	VITISV_025837	A5ADU1_VITVI	24	21,20	163,90	84,15	1,67E+09
-840	0,00	1,52	-1,77	1,77	Vitis hybrid cultivar;Vitis vinifera	Histon H3 protein (Fragment)	Tam H3	A9CSG2_9ROSI	2	28,70	15,27	7,12	4,57E+07
-840	0,00	1,60	-1,60	0,00	Vitis vinifera	Peptidase A1 domain-containing protein	VIT_00s1206g00010	F6I7L9_VITVI	4	13,50	34,60	4,93	1,57E+08
-840	1,95	2,26	-2,26	1,37	Vitis vinifera	Plasma membrane ATPase (EC 7.1.2.1)	VIT_09s0002g00130	F6HXK4_VITVI	33	41,60	104,80	323,31	3,59E+08
-840	0,00	1,84	-1,84	0,00	Vitis vinifera	Dolichyl-diphosphooligosaccharide--protein glycotransferase (EC 2.4.99.18)	VIT_17s0053g00140	F6HVR5_VITVI	3	4,00	83,37	3,88	2,20E+08
-840	0,00	1,74	-1,74	0,00	Vitis vinifera	Uncharacterized protein	VIT_00s0194g00290	F6HCW5_VITVI	7	21,90	52,52	13,74	4,21E+08
-840	0,00	1,53	-1,53	0,00	Vitis vinifera	Folate gamma-glutamyl hydrolase (EC 3.4.19.9)	VIT_19s0014g00980	F6H1W5_VITVI	2	9,10	42,29	3,60	6,49E+07
-840	1,56	2,18	-2,18	1,66	Vitis vinifera	Expansin	VIT_18s0001g01130	E0CQY0_VITVI	9	61,70	27,64	323,31	1,05E+10
-840	0,00	1,58	-1,58	0,00	Vitis vinifera	Uncharacterized protein	VIT_13s0019g04370	D7TM87_VITVI	6	15,20	66,65	5,44	3,53E+08
-840	2,78	1,61	-2,78	0,00	Vitis vinifera	Uncharacterized protein	VIT_05s0020g04900	D7T7A4_VITVI	10	32,00	47,79	41,78	1,06E+09
-840	2,02	1,54	-2,02	0,00	Vitis vinifera	VWFA domain-containing protein	VIT_04s0023g03620	D7SNX7_VITVI	8	31,90	42,84	43,30	7,26E+08
-840	1,96	1,78	-1,96	0,00	Vitis vinifera	Usp domain-containing protein	VIT_06s0004g05730	D7SK62_VITVI	6	53,00	18,02	18,31	2,41E+08
-840	1,68	1,50	-1,68	0,00	Vitis vinifera	Ubiquinol oxidase (EC 1.10.3.11)	VITISV_001908	A5BUW6_VITVI	5	22,50	36,69	23,10	4,10E+08
-840	2,22	1,84	-2,22	0,00	Vitis vinifera	SKP1-like protein	VIT_03s0038g02500	A5C7K8_VITVI	3	23,20	17,52	2,19	1,68E+08
-840	1,81	2,29	-2,29	0,00	Vitis vinifera	Proteasome subunit alpha type	VIT_09s0002g00350	F6HXL1_VITVI	10	67,10	25,98	65,89	2,30E+09
-840	0,00	1,78	-1,78	0,00	Vitis vinifera	40S ribosomal protein SA	VIT_09s0002g00610	F6HXP6_VITVI	8	30,80	33,91	135,24	2,45E+09
-840	1,52	2,08	-2,08	0,00	Vitis vinifera	Cytochrome b561 domain-containing protein	VIT_10s0003g03120	D7TK33_VITVI	6	23,00	41,13	10,70	2,65E+08
-840	0,00	1,49	-1,49	0,00	Vitis vinifera	PB1 domain-containing protein	VIT_01s0011g04140	F6HEU9_VITVI	3	9,50	58,35	4,84	1,53E+08
-840	0,00	2,05	-2,05	1,87	Vitis amurensis;Vitis vinifera	Calcium-dependent protein kinase 1d	CDPK1d	M9VTW1_9ROSI	5	13,70	52,86	9,44	3,44E+08
-828	0,00	-1,35	1,35	0,00	Vitis vinifera	Fn3_like domain-containing protein	VIT_18s0001g05180	F6H0V8_VITVI	4	9,20	84,81	6,10	1,48E+08
-828	0,00	-1,36	1,36	0,00	Vitis vinifera	Tr-type G domain-containing protein	VIT_17s0000g02860	F6GTT4_VITVI	9	24,10	57,93	25,92	6,93E+08
-828	0,00	-1,99	1,99	-1,66	Vitis vinifera	NAC-A/B domain-containing protein	VIT_13s0067g00710	D7UCZ6_VITVI	7	56,40	22,03	160,68	6,73E+08
-828	0,00	-1,55	1,55	-1,46	Vitis vinifera	Glutamate decarboxylase (EC 4.1.1.15)	VIT_04s0079g00600	D7TA34_VITVI	18	49,20	55,23	133,84	3,90E+09
-828	0,00	-1,68	1,68	-1,47	Vitis vinifera	Epimerase domain-containing protein	VIT_14s0060g00820	A5AIE0_VITVI	4	15,10	42,33	3,01	1,07E+08
-828	0,00	-1,94	1,94	0,00	Vitis vinifera	Uncharacterized protein	VIT_01s0011g04990	A5BL10_VITVI	2	25,80	10,61	4,78	1,19E+08
-828	2,07	-1,47	1,31	-2,07	Vitis vinifera	Uncharacterized protein	VIT_13s0156g00120	F6HPS4_VITVI	3	25,90	15,45	27,15	5,38E+08
-828	1,41	-1,88	1,88	-1,76	Vitis vinifera	Pectin acetylesterase (EC 3.1.1.-)	VIT_02s0087g00400	D7TFE6_VITVI	16	58,10	43,78	91,19	2,49E+09
-828	1,71	-2,10	2,10	0,00	Vitis vinifera	ATP synthase subunit gamma	VIT_17s0000g07300	D7SI12_VITVI	10	38,50	35,22	118,09	2,29E+09
-828	1,34	-2,03	2,03	0,00	Vitis vinifera	Chlorophyll a-b binding protein, chloroplastic	VIT_18s0089g01170	F6GVX0_VITVI	9	43,10	30,82	72,78	3,23E+09
-828	1,44	-2,74	2,74	-1,36	Vitis vinifera	Uncharacterized protein	VIT_00s0625g00030	F6HD75_VITVI;	7	36,20	24,08	9,68	1,54E+08
-862	-1,53	-2,30	2,30	-1,36	Vitis vinifera	UTP--glucose-1-phosphate uridylyltransferase (EC 2.7.7.9)	VIT_04s0044g00710	F6I0H8_VITVI	28	65,00	53,65	323,31	2,52E+10
-862	-1,64	-2,33	2,33	-1,50	Vitis vinifera	Beta-galactosidase (EC 3.2.1.23)	VIT_11s0016g02200	F6HGW2_VITVI	5	14,70	92,26	11,59	1,40E+08
-862	-2,76	-2,92	2,92	-1,30	Vitis vinifera	Uncharacterized protein	VIT_02s0025g02780	D7TVT2_VITVI	2	50,50	11,41	17,92	5,56E+08
-862	-2,93	-2,63	2,93	0,00	Vitis vinifera	IU_nuc_hydro domain-containing protein	VIT_08s0007g03690	D7TJ39_VITVI	3	11,40	36,85	5,33	1,59E+08
-862	-2,81	-2,62	2,81	-1,47	Vitis vinifera	Peptidyl-prolyl cis-trans isomerase (EC 5.2.1.8)	VIT_04s0023g03190	D7SP13_VITVI	3	41,50	12,90	8,95	1,72E+08
-862	-3,68	-1,93	3,68	-1,83	Vitis amurensis;Vitis vinifera	Phosphoglycerate mutase (2,3-diphosphoglycerate-independent) (EC 5.4.2.12)	PGM	E6Y5B3_9ROSI	27	70,80	61,08	323,31	1,67E+10
-862	-3,13	-2,90	3,13	-1,48	Vitis vinifera	Prohibitin	VIT_19s0085g00730	F6H9U4_VITVI	7	39,20	31,68	44,87	1,55E+08
-862	-2,91	-2,52	2,91	-2,53	Vitis vinifera	ATP-dependent Clp protease proteolytic subunit	VITISV_041995	A5BHP1_VITVI	2	10,00	31,52	1,91	1,42E+08

Cluster n°	Isabel (log2 fold-change)	Tenta (log2 fold-change)	Sciascinoso (log2 fold-change)	Aglianico (log2 fold-change)	Organism	Protein name	Gene name	UniProt name	Peptides	Sequence coverage [%]	Mol. weight [kDa]	Score	Intensity
-862	-1,85	-2,26	2,26	-1,47	Vitis vinifera	Chlorophyll a-b binding protein, chloroplastic	VIT_12s0057g00630	A5ASG6_VITVI	9	63,40	28,47	90,96	2,99E+09
-862	-2,75	-2,34	2,75	-1,65	Vitis vinifera	SHSP domain-containing protein	VIT_16s0022g00510	A5AFX4_VITVI	3	31,70	23,74	15,99	1,19E+08
-862	-2,58	-2,85	2,85	-1,52	Vitis vinifera	Cysteine proteinase inhibitor	VIT_06s0009g02230	A5ANX3_VITVI	2	26,70	11,22	7,68	1,73E+08
-862	-2,04	-1,49	2,41	-2,41	Vitis vinifera	Lipase_GDSL domain-containing protein	VIT_14s0066g00250	F6HUT2_VITVI	5	15,50	42,74	13,48	1,47E+08
-862	-1,67	0,00	2,04	-2,04	Vitis vinifera	Aldo_ket_red domain-containing protein	VIT_05s0051g00650;VITISV_003033	A5BAI3_VITVI;F6HS56_VITVI	8	29,00	36,59	46,67	1,08E+09
-862	-2,27	-1,59	2,53	-2,53	Vitis sp. ;Vitis vinifera	Phosphoribulokinase (EC 2.7.1.19)	VIT_02s0109g00080	A5BE19_VITVI	4	12,50	45,22	7,94	8,29E+07
-862	-1,39	-1,64	1,84	-1,84	Vitis vinifera	SHSP domain-containing protein	VIT_04s0008g01590	F6H3R0_VITVI	5	47,10	17,29	4,92	1,04E+08
-862	-1,88	-1,40	1,88	-1,62	Vitis vinifera	Chlorophyll a-b binding protein, chloroplastic	VITISV_031291	A5BEB1_VITVI	5	39,80	28,82	20,08	9,13E+07
-862	-1,55	0,00	2,68	-2,68	Vitis vinifera	SHSP domain-containing protein	VIT_18s0089g01270	F6GVW4_VITVI	2	13,40	21,13	12,12	1,18E+08
-862	-2,42	-1,60	2,42	-1,38	Vitis vinifera	Tubulin alpha chain	VIT_06s0004g00480	F6GUN1_VITVI	15	52,80	49,67	112,49	1,14E+09
-862	-2,82	0,00	2,82	-2,10	Vitis vinifera	Formyltetrahydrofolate synthetase (EC 6.3.4.3)	VIT_06s0004g01160	F6GUF4_VITVI	14	35,80	67,98	44,03	9,39E+08
-862	-3,03	-1,51	3,03	-2,09	Vitis labrusca x Vitis vinifera;Vitis vinifera	Clp R domain-containing protein	VITISV_012280	A5BT43_VITVI	5	8,40	100,68	19,35	1,85E+08
-862	-2,03	-1,32	2,03	-1,99	Vitis vinifera	Glycosyltransferase (EC 2.4.1.-)	VIT_16s0039g02230	D7T7R5_VITVI	10	28,10	50,03	7,56	6,85E+07
-862	-2,03	-1,32	2,03	-1,99	Vitis vinifera	UDP-glucose:flavonoid 3-O-glucosyltransferase (Fragment)	UFGT	F8U265_VITVI	10	28,10	50,03	7,56	6,85E+07
-862	-2,54	-2,81	3,84	-3,84	Vitis vinifera	Uncharacterized protein	VIT_18s0001g09460	E0CP56_VITVI	2	30,70	9,59	18,59	1,42E+08
-862	-3,00	-2,97	3,77	-3,77	Vitis vinifera	Tyrosinase_Cu-bd domain-containing protein	VIT_10s0116g00560	D7T5P6_VITVI	10	30,40	47,67	58,79	4,57E+08
-862	-2,12	-2,90	3,58	-3,58	Vitis vinifera	ATP-synt_DE_N domain-containing protein	VIT_15s0021g00920	D7SM33_VITVI	3	32,00	21,80	24,00	4,57E+08
-862	-3,98	-3,23	3,98	-3,50	Vitis vinifera	Peroxidase (EC 1.11.1.7)	VITISV_022439	A5BJV9_VITVI	4	24,80	30,85	40,09	2,45E+08
-862	-2,80	-2,86	3,66	-3,66	Vitis hybrid cultivar;Vitis pseudoreticulata (Chinese wild grapevine);Vitis vinifera	Pathogenesis-related protein 1	PR1-2	D2XZ58_9ROSI	4	60,00	17,34	14,84	1,86E+08
-862	-4,84	-3,13	4,84	-4,24	Vitis vinifera	Chalcone-flavonone isomerase family protein	VITISV_012581	A5BMU2_VITVI	8	54,60	22,91	209,94	2,94E+09
-862	-5,06	-4,78	5,06	-4,50	Vitis amurensis	Dihydroflavonol 4-reductase (DFR) (EC 1.1.1.219) (Dihydrokaempferol 4-reductase) (Flavanone 4-reductase) (FNR) (EC 1.1.1.234)	DFR	C0KY92_9ROSI	8	34,40	35,49	20,89	5,51E+08
-862	-4,32	-2,64	4,32	-4,20	Vitis pseudoreticulata (Chinese wild grapevine);Vitis vinifera	Cyclase	*N/A*	Q2I313_9ROSI	8	42,80	29,87	317,27	1,65E+10
-862	-3,91	-4,91	4,91	-3,80	Vitis amurensis;Vitis vinifera	Phenylalanine ammonia-lyase (EC 4.3.1.24)	PAL	PALY_VITVI	2	15,80	24,46	25,56	1,18E+08
-862	-4,07	-2,82	4,07	-3,45	Vitis labrusca x Vitis vinifera;Vitis vinifera	Malic enzyme/NADP-dependent malic enzyme (NADP-ME) (EC 1.1.1.40)	VVME2	O24550_VITVI	30	80,90	65,28	323,31	1,43E+10
-843	-2,90	1,64	-1,42	2,90	Vitis vinifera	Phosphopyruvate hydratase (EC 4.2.1.11)	VIT_08s0007g03960	F6HKH3_VITVI	21	65,60	47,06	323,31	1,90E+10
-843	-2,93	2,65	-1,59	2,93	Vitis vinifera	Uncharacterized protein	VIT_00s0211g00060	F6HCS7_VITVI	11	17,60	107,54	23,27	5,04E+08
-843	-1,80	1,72	0,00	1,80	Vitis vinifera	Nudix hydrolase domain-containing protein	VIT_09s0018g01600	F6HBY3_VITVI	4	5,90	88,43	3,03	1,76E+08
-843	-2,20	2,20	0,00	2,17	Vitis vinifera	UBA domain-containing protein	VITISV_014852	A5AYA9_VITVI	8	33,20	45,08	36,82	5,78E+08
-843	-1,51	1,67	-2,03	2,03	Vitis vinifera	Uncharacterized protein	VIT_01s0146g00070	D7U5Y1_VITVI	3	17,90	30,86	12,45	2,38E+08
-843	-2,19	1,74	-1,55	2,19	Vitis vinifera	AMP-binding domain-containing protein	VIT_14s0128g00720	D7U228_VITVI	4	9,20	76,89	6,62	2,07E+08
-843	-2,44	1,47	0,00	2,44	Vitis vinifera	Thioredoxin domain-containing protein	VIT_18s0075g00210	D7SRI7_VITVI	4	13,10	44,47	4,79	1,63E+08
-843	-1,64	1,64	0,00	1,46	Vitis vinifera	Uncharacterized protein	VIT_18s0001g00470	E0CQT6_VITVI	5	12,60	60,14	10,25	2,90E+08
-843	-2,24	1,57	-3,16	3,16	Vitis vinifera	Uncharacterized protein	VIT_17s0000g05650	A5C347_VITVI	2	13,00	20,56	7,83	1,13E+08
-843	-2,69	2,66	0,00	2,69	Vitis vinifera	PEP carboxylase (Fragment)	GPEPC	Q8S569_VITVI	46	57,60	110,32	319,04	1,78E+09

Cluster n°	Isabel (log2 fold-change)	Tenta (log2 fold-change)	Sciascinoso (log2 fold-change)	Aglianico (log2 fold-change)	Organism	Protein name	Gene name	UniProt name	Peptides	Sequence coverage [%]	Mol. weight [kDa]	Score	Intensity
-843	-3,14	2,52	-2,24	3,14	Vitis vinifera	Histone H4	VITISV_023523	A5B4J3_VITVI;A5BEW0_VITVI	4	40,80	11,41	23,01	1,58E+09
-843	-2,06	2,06	0,00	0,00	Vitis vinifera	9,10[9',10']carotenoid cleavage dioxygenase	CCD1	Q3T4H1_VITVI	21	51,30	61,15	138,40	2,85E+08
-843	-2,93	2,93	-1,71	1,35	Vitis vinifera	Importin N-terminal domain-containing protein	VIT_05s0062g01180	F6I4A5_VITVI	7	9,50	118,91	22,43	2,67E+08
-843	-1,33	1,50	-1,50	0,00	Vitis vinifera	Ribosomal_S7 domain-containing protein	VIT_08s0007g07220	F6HLE8_VITVI	5	32,30	25,89	111,43	7,96E+08
-843	-2,40	2,55	-2,55	1,42	Vitis vinifera	Lipocln_cytosolic_FA-bd_dom domain-containing protein	VIT_19s0177g00320	F6H9B7_VITVI	2	6,00	37,00	4,87	4,89E+07
-843	-2,01	2,01	-1,39	0,00	Vitis vinifera	Glutathione transferase (EC 2.5.1.18)	VIT_07s0005g00010	F6HPY5_VITVI	4	23,40	23,63	10,09	1,04E+08
-843	-1,39	1,39	0,00	0,00	Vitis vinifera	SHSP domain-containing protein	VIT_09s0002g06790	D7U122_VITVI	3	12,90	26,31	8,39	1,49E+08
-843	-2,00	2,00	0,00	0,00	Vitis vinifera	Inorganic diphosphatase (EC 3.6.1.1)	VIT_02s0025g02980	D7TVU8_VITVI	10	65,30	24,59	64,85	5,19E+08
-843	-1,44	1,44	0,00	0,00	Vitis vinifera	Tubulin alpha chain	VIT_08s0007g07260	D7TI76_VITVI	5	17,90	49,30	54,80	1,14E+09
-843	-2,32	2,32	0,00	0,00	Vitis vinifera	Uncharacterized protein	VIT_14s0060g02310	A5CBL0_VITVI	3	25,80	14,01	8,59	2,65E+08
-843	-1,67	1,67	0,00	0,00	Vitis vinifera	Uncharacterized protein	VIT_12s0142g00180	F6HNZ3_VITVI	3	15,30	37,78	5,51	1,70E+08
-843	-2,03	2,03	-1,44	1,33	Vitis vinifera	Alanine--glyoxylate aminotransferase (EC 2.6.1.44) (EC 2.6.1.51)	VIT_06s0009g03740	F6HA09_VITVI	6	20,00	44,34	4,39	2,68E+08
-843	-2,15	2,15	-1,85	0,00	Vitis vinifera	Uncharacterized protein	VIT_08s0040g02420	F6HQZ2_VITVI	3	35,20	15,70	8,31	2,23E+08
-843	-1,86	1,86	0,00	0,00	Vitis vinifera	Inorganic diphosphatase (EC 3.6.1.1)	VIT_15s0048g02390	F6I2Z6_VITVI	7	45,50	25,63	46,05	2,30E+08
-843	-1,85	2,42	-2,42	0,00	Vitis vinifera	Uncharacterized protein	VIT_08s0007g04520	F6HKE5_VITVI	2	13,10	39,90	13,11	1,38E+08
-843	-1,96	1,96	0,00	0,00	Vitis vinifera	Epimerase domain-containing protein	VITISV_002160	A5AIL5_VITVI	2	11,60	31,58	3,51	1,38E+08
-843	-1,96	1,96	0,00	0,00	Vitis vinifera	NAD(P)-bd_dom domain-containing protein	VIT_02s0012g01900	D7TTS6_VITVI	2	11,60	31,58	3,51	1,38E+08
-843	-1,84	1,84	-1,44	0,00	Vitis vinifera	PSI subunit V	VIT_04s0023g00410	A5AW35_VITVI	2	13,60	23,30	2,41	1,19E+08
-824	-3,84	-3,46	3,33	3,84	Vitis vinifera	Uncharacterized protein	VIT_15s0046g03570	F6I6H7_VITVI	7	23,20	32,20	14,70	2,32E+08
-824	-4,06	-2,61	2,09	4,06	Vitis vinifera	L-ascorbate peroxidase (EC 1.11.1.11)	VITISV_009208	A5AK21_VITVI	13	41,90	46,88	93,65	2,31E+09
-824	-3,41	-3,06	2,60	3,41	Vitis vinifera	Malic enzyme	VIT_15s0021g00500	D7SM51_VITVI	15	36,20	69,34	110,03	1,46E+09
-824	-3,37	-3,34	3,37	3,34	Vitis vinifera	Uncharacterized protein	VIT_02s0012g00710	D7TTF7_VITVI	2	14,50	20,01	43,62	6,33E+08
-824	-2,66	-3,69	2,03	3,69	Vitis vinifera	Photosystem II protein D1 (PSII D1 protein) (EC 1.10.3.9)	VIT_13s0019g02630	F6HNS1_VITVI	6	19,30	38,89	95,14	3,85E+09
-824	-2,97	-3,75	3,75	3,27	Vitis vinifera	Clp R domain-containing protein	VIT_04s0008g05870	D7SUY2_VITVI	7	11,50	98,89	13,87	2,19E+08
-824	-3,79	-2,36	3,79	2,13	Vitis vinifera	5-methyltetrahydropteroyltriglutamate--homocysteine S-methyltransferase (EC 2.1.1.14)	VITISV_018473	A5C7K7_VITVI	31	47,60	84,99	245,40	6,40E+09
-824	-3,64	-2,16	3,64	2,35	Vitis vinifera	Uncharacterized protein	VITISV_011171	A5BGC2_VITVI	2	29,10	17,10	7,82	3,41E+08
-824	-3,67	-2,17	3,67	2,65	Vitis vinifera	Phosphoinositide phospholipase C (EC 3.1.4.11)	VIT_08s0105g00300	D7SY09_VITVI	7	21,20	62,73	9,53	3,25E+08
-824	-3,34	-1,56	3,34	1,89	Vitis vinifera	3-hydroxyacyl-CoA dehydrogenase (EC 1.1.1.35) (EC 4.2.1.17) (EC 5.1.2.3) (EC 5.3.3.8) (Enoyl-CoA hydratase/3-2-trans-enoyl-CoA isomerase/3-hydroxybutyryl-CoA epimerase)	VIT_11s0016g03690;	D7TBL4_VITVI	4	9,70	78,37	15,06	2,56E+08
-824	-3,61	-1,83	3,61	2,43	Vitis amurensis;Vitis hybrid cultivar;Vitis labrusca x Vitis vinifera;Vitis rotundifolia (Muscadine grape);Vitis vinifera	Chalcone synthase (EC 2.3.1.74)	CHS	A2ICC5_VITVI	9	39,90	42,92	22,27	5,65E+08
-824	-4,04	-2,50	4,04	2,11	Vitis vinifera	Cytochrome P450 CYP76Y1 (EC 1.14.14.1)	VIT_02s0012g02810	A5C7X2_VITVI	7	22,40	55,74	59,56	4,19E+08
-824	-3,35	-2,25	3,35	2,83	Vitis vinifera	Uncharacterized protein	VIT_00s0454g00040	F6I2D0_VITVI	8	16,10	75,52	24,55	6,11E+08
-824	-3,47	-3,06	3,47	2,71	Vitis pseudoreticulata (Chinese wild grapevine);Vitis vinifera	Epimerase domain-containing protein	VIT_06s0061g01120	D7SN69_VITVI	8	27,60	43,57	45,83	1,47E+08
-824	-3,47	-3,06	3,47	2,71	Vitis pseudoreticulata (Chinese wild grapevine);Vitis vinifera	UDP-D-apiose/UDP-D-xylose synthase	*N/A*	Q2LAM6_9ROSI	8	27,60	43,57	45,83	1,47E+08
-824	-3,91	-2,79	3,91	3,47	Vitis vinifera	Profilin	VIT_04s0008g00490	A5AQ89_VITVI	3	36,60	14,06	28,89	4,33E+08
-824	-3,63	-1,88	3,47	3,63	Vitis vinifera	Diadenosine tetraphosphate synthetase (EC 6.1.1.14)	VIT_01s0150g00150	F6HJ70_VITVI	20	30,40	76,33	78,63	1,55E+09

Cluster n°	Isabel (log2 fold-change)	Tenta (log2 fold-change)	Sciascinoso (log2 fold-change)	Aglianico (log2 fold-change)	Organism	Protein name	Gene name	UniProt name	Peptides	Sequence coverage [%]	Mol. weight [kDa]	Score	Intensity
-824	-2,56	-2,83	2,83	1,64	Vitis vinifera	Thaumatin-like protein	*N/A*	A3QRB4_VITVI	8	35,60	23,86	149,02	3,41E+08
-824	-3,15	-2,64	3,15	2,54	Vitis vinifera	Uncharacterized protein	VIT_14s0060g00690	F6I4V3_VITVI	9	57,30	21,18	124,44	3,01E+08
-824	-2,53	-3,09	3,09	1,78	Vitis vinifera	Tubulin beta chain	VIT_18s0001g14360	F6H1L2_VITVI	17	56,70	50,25	182,93	2,00E+08
-824	-2,17	-2,86	2,86	2,07	Vitis vinifera	Prolyl-tRNA synthetase (EC 6.1.1.15)	VIT_07s0005g02620	D7U2U2_VITVI	5	15,30	58,72	13,95	1,27E+08
-824	-3,07	-2,54	3,07	2,43	Vitis vinifera	Glutaredoxin-dependent peroxiredoxin (EC 1.11.1.25)	VITISV_023716	A5ARL2_VITVI	10	80,20	17,26	272,29	7,20E+09
-824	-2,47	-2,96	2,96	2,69	Vitis vinifera	ATP-dependent 6-phosphofructokinase (ATP-PFK) (Phosphofructokinase) (EC 2.7.1.11) (Phosphohexokinase)	PFK	D7TBD7_VITVI	7	22,80	53,94	8,77	1,73E+08
-824	-3,05	-2,11	3,05	2,30	Vitis vinifera	Acetate--CoA ligase (EC 6.2.1.1)	VIT_14s0068g00640	D7SVD4_VITVI	3	7,80	84,77	31,99	1,83E+08
-824	-2,84	-2,77	2,84	2,27	Vitis vinifera	6-phosphogluconate dehydrogenase, decarboxylating (EC 1.1.1.44)	VIT_02s0025g00900	F6HUQ8_VITVI	13	36,40	53,90	93,92	1,11E+09
-824	-2,45	-2,22	2,45	1,60	Vitis vinifera	Acetohydroxy-acid reductoisomerase (Alpha-keto-beta-hydroxylacyl reductoisomerase)	VITISV_003232	A5ARF9_VITVI	9	25,50	63,49	56,34	1,84E+09
-824	-2,45	-2,22	2,45	1,60	Vitis vinifera	Ketol-acid reductoisomerase (EC 1.1.1.86) (Acetohydroxy-acid reductoisomerase) (Alpha-keto-beta-hydroxylacyl reductoisomerase)	VIT_12s0028g02340	A5AGN5_VITVI	9	25,50	63,49	56,34	1,84E+09
-824	-2,45	-2,22	2,45	1,60	Vitis vinifera	KARI N-terminal Rossmann domain-containing protein	VIT_10s0003g01180	F6HM93_VITVI	9	25,50	63,49	56,34	1,84E+09
-824	-3,09	-2,21	3,09	1,79	Vitis vinifera	Clathrin heavy chain	VIT_13s0067g01290	A5ACP0_VITVI	41	33,80	192,97	2,86	2,07E+08
-824	-3,27	-2,45	3,27	2,05	Vitis vinifera	Uncharacterized protein	VIT_19s0014g01570	A5ADJ5_VITVI	3	12,00	45,42	36,00	1,74E+08
-824	-3,43	-2,27	2,64	3,43	Vitis vinifera	Adenosylhomocysteinase (EC 3.3.1.1)	VIT_17s0000g09840	F6GTM7_VITVI	22	60,20	53,08	101,79	1,10E+09
-824	-2,99	-1,44	2,62	2,99	Vitis vinifera	Ubiquitin carboxyl-terminal hydrolase (EC 3.4.19.12)	VIT_09s0002g02500	D7U032_VITVI	5	17,30	54,05	10,87	3,76E+08
-824	-2,91	-2,80	2,12	2,91	Vitis vinifera	TRASH domain-containing protein	VIT_16s0100g00650	F6HP56_VITVI	2	14,70	18,54	12,92	1,54E+08
-824	-3,25	-2,38	2,74	3,25	Vitis vinifera	Uncharacterized protein	VIT_05s0049g01790	F6HZQ0_VITVI	4	36,40	15,03	11,91	2,02E+08
-824	-3,10	-1,88	2,39	3,10	Vitis vinifera	WD_REPEATS_REGION domain-containing protein	VIT_17s0000g02750	A5BV59_VITVI	14	52,60	36,04	202,08	3,20E+09
-824	-3,22	-2,05	2,05	3,22	Vitis vinifera	Uncharacterized protein	VIT_01s0011g00560	A5B3K2_VITVI	8	60,00	20,92	107,67	5,24E+09
-824	-3,23	-2,33	2,12	3,23	Vitis vinifera	Coatomer subunit beta (Beta-coat protein)	VIT_09s0018g02080	F6HBV4_VITVI	10	15,60	105,92	44,58	7,97E+08
-824	-3,09	-2,71	2,78	3,09	Vitis vinifera	Xylulose kinase (EC 2.7.1.17)	VIT_18s0001g08260	E0CNV3_VITVI	5	17,10	60,96	7,95	2,76E+08
-824	-2,42	-1,98	2,42	2,27	Vitis vinifera	Pyrophosphate--fructose 6-phosphate 1-phosphotransferase subunit alpha (PFP) (6-phosphofructokinase, pyrophosphate dependent) (PPi-PFK) (Pyrophosphate-dependent 6-phosphofructose-1-kinase)	PFP-ALPHA	F6I6W5_VITVI	21	44,30	67,34	159,28	2,66E+09
-824	-2,64	-1,56	2,50	2,64	Vitis vinifera	Uncharacterized protein	VIT_18s0041g00430	F6I407_VITVI	25	49,80	67,75	259,30	6,54E+09
-824	-1,31	-2,55	2,32	2,55	Vitis vinifera	N-acetyltransferase domain-containing protein	VIT_13s0019g04540	F6HNE8_VITVI	4	19,80	24,93	8,87	1,99E+08
-824	-2,51	-2,51	1,37	2,51	Vitis vinifera	Pyruvate, phosphate dikinase (EC 2.7.9.1)	VIT_05s0020g02310	F6HDJ6_VITVI	5	12,40	110,54	25,40	3,54E+08
-824	-2,16	-2,67	2,26	2,67	Vitis vinifera	Aspartate aminotransferase (EC 2.6.1.1)	VIT_11s0016g03720	D7TBL7_VITVI	22	70,30	45,12	259,35	2,89E+09
-824	-2,28	-2,57	2,57	2,54	Vitis vinifera	Beta-galactosidase (EC 3.2.1.23)	VIT_07s0031g02480	D7SWF1_VITVI	4	7,60	94,94	3,90	1,50E+08
-824	-2,65	-2,72	1,83	2,72	Vitis vinifera	EGF_CA domain-containing protein	VIT_03s0091g00740	D7SXR1_VITVI	4	10,90	65,62	8,72	1,19E+08
-824	-1,50	-2,51	1,75	2,51	Vitis vinifera	NAD(P)H-hydrate epimerase (EC 5.1.99.6) (NAD(P)HX epimerase)	VIT_18s0001g07100	E0CRS5_VITVI	20	5,40	58,89	2,17	1,32E+08
-824	-1,50	-2,51	1,75	2,51	Vitis vinifera	Pyridoxal 5'-phosphate synthase (EC 1.4.3.5)	VITISV_002641	A5AZ07_VITVI	2	5,40	58,89	2,17	1,32E+08
-824	-2,71	-1,98	1,80	2,71	Vitis vinifera	Pectinesterase (EC 3.1.1.11)	VITISV_003251	A5AUK1_VITVI	2	4,50	58,01	13,12	1,63E+08
-824	-2,37	-2,05	2,26	2,37	Vitis amurensis;Vitis vinifera	Flavonoid 3' hydroxylase	VvF3'h1	Q3C214_VITVI	9	26,50	56,03	41,43	4,86E+08
-824	-2,65	-2,08	2,65	2,51	Vitis vinifera	UBC core domain-containing protein	VIT_06s0004g08200	D7SJH9_VITVI	2	25,70	16,55	5,05	5,22E+08
-470	-1,68	0,00	1,68	0,00	Vitis vinifera	Putative pectin methylesterase inhibitor (Putative ripening-related protein)	grip28	Q9M4H8_VITVI	8	59,60	21,13	323,31	5,95E+09
-470	-1,38	0,00	1,38	0,00	Vitis vinifera	SHSP domain-containing protein	VIT_04s0008g01520	F6H3Q3_VITVI	5	42,90	17,58	17,97	9,52E+07
-470	-1,66	0,00	1,66	0,00	Vitis vinifera	LRRNT_2 domain-containing protein	VIT_06s0004g01750	F6GU89_VITVI	4	14,80	39,27	5,62	5,25E+07
-470	-1,34	0,00	1,34	0,00	Vitis vinifera	Protein kinase domain-containing protein	VIT_05s0124g00360	D7U8V6_VITVI	4	17,20	44,28	7,76	1,21E+08
-470	-1,66	0,00	1,66	-1,64	Vitis vinifera	Glutathione transferase (EC 2.5.1.18)	VIT_07s0104g01800	D7TP00_VITVI	4	26,70	24,64	15,11	1,33E+08
-470	-2,34	0,00	2,34	-1,37	Vitis vinifera	Triosephosphate isomerase	VIT_13s0019g01090	D7TLU7_VITVI	11	66,90	27,13	7,48	8,08E+07
-470	-1,75	0,00	1,75	0,00	Vitis vinifera	Aldo_ket_red domain-containing protein	VITISV_013444	A5BFL9_VITVI	7	30,30	35,60	19,20	2,85E+08
-470	-1,55	0,00	1,55	-1,31	Vitis vinifera	Cytochrome b5 heme-binding domain-containing protein	VIT_04s0008g06870	A5BA15_VITVI	2	31,30	10,85	29,55	8,28E+07
-470	-1,90	0,00	1,90	0,00	Vitis vinifera	12-oxophytodienoate reductase 3 protein (EC 1.3.1.42)	OPR3	D7TCG0_VITVI	3	17,00	40,29	16,66	1,61E+08
-470	-1,70	0,00	1,70	0,00	Vitis sp. ;Vitis vinifera	ATP synthase subunit beta (EC 7.1.2.2)	VIT_01s0011g04490	F6HFL0_VITVI	27	71,30	59,62	323,31	1,11E+08
-470	-1,79	0,00	1,79	0,00	Vitis vinifera	N-acetyl-glutamate semialdehyde dehydrogenase (EC 1.2.1.38)	VIT_03s0063g01110	F6HQF0_VITVI	4	17,60	36,05	6,41	9,55E+07

Cluster n°	Isabel (log2 fold-change)	Tenta (log2 fold-change)	Sciascinoso (log2 fold-change)	Aglianico (log2 fold-change)	Organism	Protein name	Gene name	UniProt name	Peptides	Sequence coverage [%]	Mol. weight [kDa]	Score	Intensity
-470	-1,53	0,00	1,53	-1,37	Vitis vinifera	Chlorophyll a-b binding protein, chloroplastic	Lhca1	Q6XGX8_VITVI	2	53,10	10,75	1,61	9,72E+07
-470	-2,19	0,00	2,19	0,00	Vitis vinifera	SAM_MPBQ_MSBQ_MT domain-containing protein	VIT_00s0179g00300	F6HBJ2_VITVI	3	12,90	38,41	28,22	8,43E+07
-823	-1,35	-2,12	2,12	1,55	Vitis vinifera	Usp domain-containing protein	VITISV_015456	A5BWI4_VITVI	3	24,90	36,03	42,99	3,83E+08
-823	-1,68	-1,76	1,76	1,56	Vitis vinifera	Peptidylprolyl isomerase (EC 5.2.1.8)	VIT_14s0066g01410	D7TWR2_VITVI	3	13,80	49,18	5,46	1,21E+08
-823	-1,39	-1,49	1,49	1,32	Vitis vinifera	Uncharacterized protein	VIT_11s0118g00590	D7T4Z4_VITVI	2	8,30	61,18	56,11	3,59E+08
-823	-1,81	-2,56	2,56	1,63	Vitis vinifera	Eukaryotic translation initiation factor 6 (eIF-6)	EIF6	A5BR49_VITVI	4	29,00	26,49	16,65	4,71E+08
-823	-1,72	-1,62	1,43	1,72	Vitis labrusca x Vitis vinifera;Vitis vinifera	Anthocyanidin synthase (EC 1.14.11.19)	ANS	A2ICC9_VITVI	15	55,50	40,19	220,71	4,73E+08
-823	-1,72	-1,62	1,43	1,72	Vitis labrusca x Vitis vinifera;Vitis vinifera	Leucoanthocyanidin dioxygenase (LDOX) (Leucocyanidin oxygenase) (EC 1.14.20.4) (Leucoanthocyanidin hydroxylase)	KyLDOX1	Q8LP73_9ROSI	15	55,50	40,19	220,71	4,73E+08
-823	0,00	-2,24	2,24	1,50	Vitis vinifera	Profilin	VIT_09s0002g03250	A5ASF9_VITVI	3	36,60	14,23	3,65	6,45E+07
-823	-1,38	-1,53	1,53	0,00	Vitis vinifera	eIF2B_5 domain-containing protein	VIT_06s0004g08180	F6GU78_VITVI	3	13,80	30,35	3,19	9,74E+07
-823	-1,65	-1,41	1,65	0,00	Vitis vinifera	Proteasome subunit beta (EC 3.4.25.1)	VIT_18s0001g02260	E0CR38_VITVI	4	39,20	22,92	121,92	9,78E+08
-823	-1,82	-1,44	1,82	0,00	Vitis vinifera	Lactamase_B domain-containing protein	VIT_13s0067g00180	D7T4T3_VITVI	3	13,20	28,67	4,82	1,16E+08
-823	-1,82	-1,94	1,94	0,00	Vitis vinifera	Aconitate hydratase (Aconitase) (EC 4.2.1.3)	VIT_00s0264g00030	D7T1R6_VITVI	19	32,90	100,45	39,09	6,82E+08
-823	-1,82	-1,96	1,96	0,00	Vitis vinifera	Clathrin heavy chain	VIT_06s0004g06860	D7SJV3_VITVI	47	41,00	192,95	224,17	5,73E+08
-823	-1,66	-2,19	2,19	0,00	Vitis vinifera	Uncharacterized protein	VIT_16s0050g00080	A5C962_VITVI	5	32,70	21,76	17,13	2,16E+08
-823	-1,90	-2,25	2,25	0,00	Vitis vinifera	Uncharacterized protein	VIT_06s0004g04440	F6GV28_VITVI	4	21,70	27,33	10,52	1,27E+08
-823	-1,83	-2,24	2,24	0,00	Vitis vinifera	Thioredoxin-dependent peroxiredoxin (EC 1.11.1.24)	VIT_08s0007g02490	D7TH54_VITVI	11	47,60	30,17	174,43	3,15E+09
-823	-2,19	-2,10	2,19	0,00	Vitis vinifera	40S ribosomal protein S7	VIT_17s0000g03530	F6GTN2_VITVI	6	44,00	22,11	35,15	5,46E+08
-823	-1,85	-1,45	1,85	0,00	Vitis vinifera	Expansin	VIT_01s0026g02620	A5AIN5_VITVI	3	19,50	26,20	31,02	6,91E+08
-823	-1,78	-1,77	1,78	0,00	Vitis vinifera	Peroxin-14	VIT_17s0000g08410	D7SHS0_VITVI	3	12,00	57,31	3,58	1,35E+08
-777	-4,48	4,48	-4,30	2,72	Vitis vinifera	Triosephosphate isomerase	VIT_03s0038g01780	F6I134_VITVI	10	38,30	34,68	49,12	1,26E+09
-777	-3,54	2,08	-2,93	3,54	Vitis vinifera	Thioredoxin reductase (EC 1.8.1.9)	VIT_04s0044g01750	F6I0C1_VITVI	12	45,60	39,67	118,80	1,87E+09
-777	-3,72	3,72	-2,32	2,28	Vitis vinifera	Carboxypeptidase (EC 3.4.16.-)	VIT_07s0141g00520	F6HWI4_VITVI	4	10,10	55,84	49,12	3,31E+08
-777	-2,89	2,74	-3,37	3,37	Vitis vinifera	Uncharacterized protein	VIT_13s0019g01430	F6HNX5_VITVI	30	57,80	71,17	323,31	1,01E+10
-777	-3,91	3,20	-2,24	3,91	Vitis vinifera	Bet_v_1 domain-containing protein	VIT_01s0011g05170	F6HFH1_VITVI	3	20,50	17,08	14,32	3,91E+08
-777	-6,11	6,11	-6,09	5,34	Vitis vinifera	Bet_v_1 domain-containing protein	VIT_05s0077g01670	F6H6V1_VITVI	5	30,00	22,96	20,37	3,14E+08
-777	-4,01	3,28	-4,99	4,99	Vitis vinifera	Bet_v_1 domain-containing protein	VIT_05s0077g01540	F6H6U5_VITVI	11	78,60	17,27	78,66	7,02E+08
-777	-7,51	5,13	-5,36	7,51	Vitis vinifera	Glucose acyltransferase 2	VIT_03s0091g01290	F6H684_VITVI	12	36,10	54,93	56,01	1,72E+09
-777	-3,43	3,43	-3,31	2,45	Vitis vinifera	Uncharacterized protein	VIT_14s0068g01370	F6H446_VITVI	2	16,40	20,32	11,03	3,19E+08
-777	-4,61	4,61	-2,61	2,89	Vitis vinifera	Uncharacterized protein	VITISV_006707	A5AT21_VITVI	17	32,20	75,63	144,35	2,42E+09
-777	-5,29	4,57	-6,58	6,58	Vitis vinifera	Uncharacterized protein	VIT_02s0025g03280	D7TVX5_VITVI	22	72,30	55,41	192,50	3,26E+09
-777	-5,39	3,48	-5,00	5,39	Vitis vinifera	Xylose isomerase (EC 5.3.1.5)	VIT_11s0052g01710	D7SQ37_VITVI	22	53,20	58,76	143,38	3,72E+09
-777	-4,59	4,60	-4,60	4,34	Vitis pseudoreticulata (Chinese wild grapevine);Vitis vinifera	Class IV chitinase	Chi4D	B0FZ26_9ROSI	6	46,00	27,31	323,31	2,57E+09
-777	-3,84	4,20	-4,20	4,11	Vitis vinifera	L-ascorbate peroxidase (EC 1.11.1.11)	VITISV_005618	A5BKT3_VITVI	8	45,50	27,99	35,91	5,02E+08
-777	-3,32	3,32	-2,52	2,59	Vitis vinifera	60S ribosomal protein L18a	VIT_19s0135g00100	D7SX32_VITVI	7	39,30	21,26	10,10	4,31E+08
-777	-4,61	3,90	-3,50	4,61	Vitis labrusca x Vitis vinifera;Vitis vinifera	Polyphenol_oxidase	PPO2	A0A024FS61_9ROSI	7	21,90	67,12	70,45	9,39E+08
-777	-4,65	4,65	-3,30	3,84	Vitis vinifera	AAA domain-containing protein	VITISV_000418	A5AER7_VITVI	11	22,20	74,32	33,83	5,23E+08
-861	-6,19	-6,22	6,22	5,48	Vitis vinifera	Catalase (EC 1.11.1.6)	GCat	Q8S568_VITVI	23	65,00	56,98	37,48	1,20E+09
-861	-7,34	-7,56	6,47	7,56	Vitis vinifera	Mitochondrial protein YMF19 (EC 7.1.2.2)	VIT_00s0246g00220	F6HML1_VITVI	4	32,70	18,09	30,40	4,49E+08
-861	-6,77	-6,07	6,77	6,50	Vitis vinifera	NAD(P)-bd_dom domain-containing protein	VIT_13s0019g00840	A5BIN1_VITVI	15	63,20	38,79	298,03	3,33E+09
-861	-6,38	-5,96	5,98	6,38	Vitis vinifera	Uncharacterized protein	VIT_04s0044g01110	A5C0I8_VITVI	18	74,50	41,12	239,77	1,08E+09
-861	-4,24	-4,59	4,59	3,98	Vitis vinifera	Usp domain-containing protein	VIT_05s0077g01070	F6H727_VITVI	4	30,90	18,06	14,38	4,94E+08
-861	-4,55	-3,83	4,32	4,55	Vitis vinifera	Uncharacterized protein	VIT_02s0012g00100	F6GVD3_VITVI	2	26,10	10,27	17,82	1,51E+08
-861	-4,84	-2,86	4,10	4,84	Vitis vinifera	Uncharacterized protein	VIT_06s0061g00340	F6GWA3_VITVI	31	55,50	89,59	323,31	2,72E+09

Cluster n°	Isabel (log2 fold-change)	Tenta (log2 fold-change)	Sciascinoso (log2 fold-change)	Aglianico (log2 fold-change)	Organism	Protein name	Gene name	UniProt name	Peptides	Sequence coverage [%]	Mol. weight [kDa]	Score	Intensity
-861	-3,57	-4,20	4,01	4,20	Vitis vinifera	Xyloglucan endotransglucosylase/hydrolase (EC 2.4.1.207)	VIT_06s0061g00550	D7SNC1_VITVI	7	47,40	32,70	105,95	3,46E+08
-861	-4,76	-3,96	4,76	4,74	Vitis vinifera	Epimerase domain-containing protein	VIT_14s0030g02180	F6HTR2_VITVI	16	54,80	42,54	137,46	2,61E+08
-861	-3,60	-4,40	4,40	3,74	Vitis vinifera	Uncharacterized protein	VIT_02s0025g04250	F6HUG3_VITVI	4	18,20	24,24	44,73	2,64E+08
-861	-4,93	-4,44	4,30	4,93	Vitis vinifera	Uncharacterized protein	VIT_03s0088g00260	D7T3Q1_VITVI	8	24,50	53,14	59,60	1,22E+09
-861	-4,98	-4,88	3,46	4,98	Vitis riparia (Frost grape) (Vitis vulpina);Vitis vinifera	Mannan endo-1,4-beta-mannosidase (EC 3.2.1.78)	VIT_19s0014g02880	F6H255_VITVI	3	22,60	24,57	10,89	1,83E+08
-861	-5,31	-4,91	5,31	5,24	Vitis vinifera	Putative photosystem I reaction center subunit N (Fragment)	PsaN	Q6XGX6_VITVI	3	38,80	9,80	21,27	1,09E+09
-861	-6,12	-5,60	4,56	6,12	Vitis vinifera	Carboxypeptidase (EC 3.4.16.-)	VITISV_020424	A5AIW7_VITVI	11	37,20	53,20	77,20	1,49E+09
-861	-5,14	-4,99	5,14	5,14	Vitis heyneana;Vitis vinifera	Aquaporin PIP1;3 (Fragment)"	PIP1;3	A3FA65_VITVI	10	48,10	30,71	8,03	5,27E+08
-861	-5,26	-5,16	5,09	5,26	Vitis vinifera	Ribosomal_L7Ae domain-containing protein	VIT_18s0001g04760	F6H0V0_VITVI	2	25,00	12,31	4,88	2,90E+08
-861	-5,06	-4,48	4,32	5,06	Vitis vinifera	Ribosomal protein	VIT_08s0007g03340	F6HKK9_VITVI	6	21,70	32,53	14,26	4,56E+08
-861	-5,18	-4,97	5,18	4,69	Vitis vinifera	SCP domain-containing protein	VIT_03s0088g00710	F6HBN7_VITVI	3	39,10	17,53	19,12	3,47E+08
-861	-5,16	-4,80	5,16	4,88	Vitis pseudoreticulata (Chinese wild grapevine);Vitis vinifera	Barwin domain-containing protein	VIT_14s0081g00030	D7TXF5_VITVI	5	79,00	15,11	323,31	5,87E+09
-861	-5,06	-4,93	5,06	4,67	Vitis hybrid cultivar;Vitis vinifera	Putative thaumatin-like protein	Tam-TLP	A9CSP0_9ROSI	13	50,50	24,05	155,09	3,54E+09
-861	-5,24	-5,30	5,30	4,63	Vitis vinifera	Uncharacterized protein	VIT_02s0025g04230	A5B4P9_VITVI	5	21,30	24,14	10,46	2,23E+09
-861	-4,49	-4,95	4,95	3,97	Vitis vinifera	Uncharacterized protein	VIT_03s0017g01210	D7TU92_VITVI	3	12,80	30,01	22,80	2,08E+08
